# A diversified parts list for mammalian genome engineering and molecular recording

**DOI:** 10.1038/s41587-025-02896-2

**Published:** 2025-11-11

**Authors:** Troy A. McDiarmid, Megan L. Taylor, Wei Chen, Florence M. Chardon, Junhong Choi, Hanna Liao, Xiaoyi Li, Haedong Kim, Jean-Benoît Lalanne, Tony Li, Jenny F. Naathans, Beth K. Martin, Jordan Knuth, Alessandro L.V. Coradini, Jesse M. Gray, Sudarshan Pinglay, Jay Shendure

**Affiliations:** 1 Department of Genome Sciences, University of Washington, Seattle, WA, USA; 2 Seattle Hub for Synthetic Biology, Seattle, WA, USA; 3 Developmental Biology Program, Memorial Sloan Kettering Cancera Center, New York, NY, USA; 4 Brotman Baty Institute for Precision Medicine, Seattle, WA, USA; 5 Howard Hughes Medical Institute, Seattle, WA, USA; 6 Allen Discovery Center for Cell Lineage Tracing, Seattle, WA, USA

## Abstract

A standardized ‘parts list’ of sequences for genetic engineering of microbes has been indispensable to progress in synthetic biology, but few analogous parts exist for mammalian systems. Here, we design libraries of extant, ancestral, mutagenized or miniaturized variants of Pol III promoters and guide RNA (gRNA) scaffolds and quantify their ability to mediate precise edits to the mammalian genome via multiplex prime editing. We identify thousands of parts that reproducibly drive a range of editing activities in human and mouse cell lines, including hundreds with greater activity than commonly used sequences. Saturation mutagenesis screens identify tolerated sequence variants that further enhance sequence diversity. In an application to molecular recording, we design a ‘ten key’ array that, in mammalian cells, achieves balanced activity of prime editing gRNAs as predicted by the activity of the component parts. The data reported here will aid the design of synthetic loci encoding arrays of gRNAs exhibiting predictable, differentiated levels of activity for applications in multiplexed perturbation, biological recorders and complex genetic circuits.

A central goal of synthetic biology is the design, synthesis and deployment of complex genetic circuits that measure and/or manipulate biological systems^1–8^. The components used in such circuits are often described as a “parts list”, wherein each “part” behaves and interacts with other exogenous parts (or endogenous factors) in a predictable manner, analogous to the parts lists of other engineering disciplines, *e.g.* the resistors, capacitors and inductors of electrical circuits^9,10^. Until recently, most work in this space has focused on designing and characterizing parts for bacteria or yeast, *i.e.* organisms that are routinely engineered for various goals. However, there is a growing demand for genetic parts that function predictably in mammalian systems as well.

Presently, the number of such parts that are functionally validated and characterized for mammalian genome engineering remains limited. For example, to drive guide RNA (gRNA) expression for CRISPR applications, the field overwhelmingly relies on a handful of endogenous human Pol III promoters (usually U6, sometimes H1 or 7SK), and for gRNA scaffolds, on a handful of designs derived from *S. pyogenes*^5,11–15^. Validation and quantitative assessment of larger sets of promoters and/or scaffolds would enable the levels of genome editing to be programmed during construct design. The development of such a parts list also has the potential to identify sequences with greater activity than the standard components.

For a subset of goals, a mammalian genome engineering parts lists would ideally be non-repetitive or minimally repetitive at a sequence level, as has been achieved for analogous bacterial parts lists^16,17^. For example, we and others have envisioned multiplex cell lineage recorders that rely on many instances of Pol III promoters, gRNAs and target sites, ideally encoded at a single locus to facilitate the generation of distributable “recorder cell lines” and “recorder mice”^18–22^. However, such parts, typically encoded as DNA, are often unstable if repetitive, *i.e.* if the same subsequence appears repeatedly in different parts that are used across the same *cis-*encoded circuit. The challenges associated with repetitive subsequences manifest at nearly every step, but are most problematic during synthesis and assembly^23–26^. For example, although yeast-based assembly can now be used to construct entirely synthetic loci that are over 100 kilobases^27–30^, the homologous recombination (HR) mechanisms that enable yeast-based assembly also corrupt the process if the same subsequence appears repeatedly. Consequently, the same part cannot easily be used more than once in a yeast-assembled, single-locus, mammalian-deployed genetic circuit.

In this study, we sought to address this by first designing diverse libraries of Pol III promoters and gRNA scaffolds, and then quantifying their activity with a multiplex prime editing-based functional assay. Through these experiments, we validate and characterize thousands of sequence-diverse parts capable of driving genome editing in human and mouse cancer and stem cell lines. Both Pol III promoter and gRNA scaffold variants exhibited highly reproducible activities spanning several orders of magnitude, including parts that are more compact and/or more active than the most widely used sequences. Finally, we demonstrate how these diversified promoters and gRNA scaffolds can be leveraged to design multi-component synthetic loci that are easily assembled in yeast. Specifically, we design and assemble a single locus, 10-key diversified molecular recording array^31^, and demonstrate that its tandemly arranged parts function as predicted in mammalian cells.

## Results

### Design, synthesis and functional characterization of diversified U6 promoters

**Figure 1 |:**
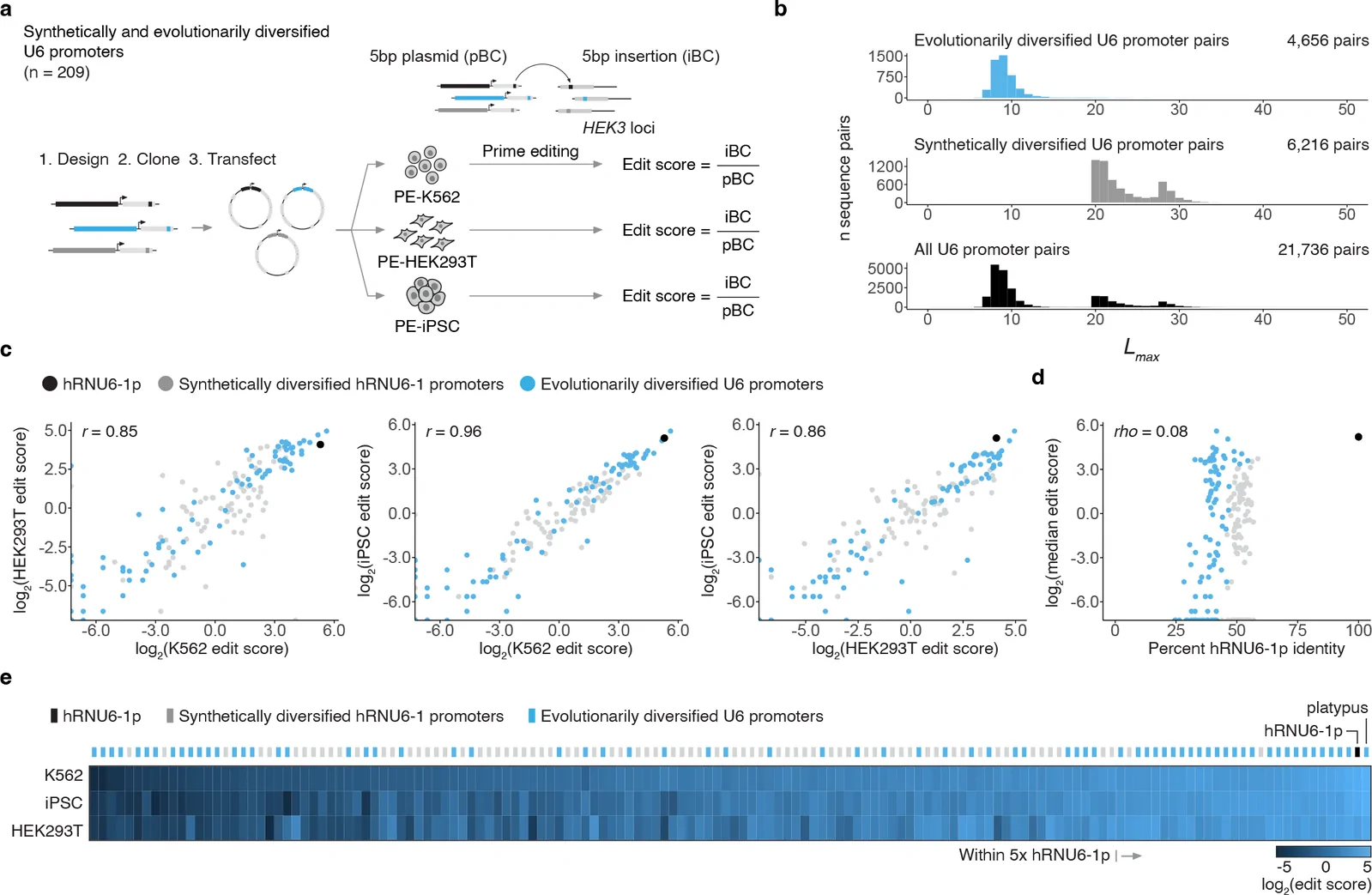
Multiplex functional characterization of synthetically and evolutionarily diversified U6 promoters in human cells. **a)** Synthetically and evolutionarily diversified U6 promoters were tested in three human cellular contexts with a multiplex prime editing functional assay. Edit scores were defined as the frequency of an insertional barcode (iBC) at the genomic target site divided by the frequency of the same barcode in the plasmid library (pBC). **b)** *L*_*max*_ distributions quantifying the maximal shared repeat length between all possible pairs of sequences for the evolutionarily diversified U6 promoter library (n = 97; 4,656 pairs), the synthetically diversified hRNU6–1p library (n = 112; 6,216 pairs) and the combined set (n = 209; 21,736 pairs) in the same orientation. See Supplementary Fig. 1b for *L*_*max*_ distributions for reverse complement comparisons. **c)** Pairwise comparison of log-transformed edit scores between cellular contexts. Pearson correlations, calculated on barcode-normalized edit scores prior to log transformation, are shown. **d)** Sequence identity with hRNU6–1p (*x-*axis) is not predictive of functional activity of synthetically or evolutionarily diversified U6 promoters. Spearman correlation is shown. **e)** Edit scores of 146 functional diversified U6 promoters ordered left-to-right by ascending median edit score across three human cellular contexts.

To date, only a handful of Pol III promoters have been characterized for genome engineering in mammalian cells^11,15,32^. To identify sequence- and activity-diversified promoters, we piloted two complementary approaches to design ~200 diversified Pol III U6 promoters (~100 via evolutionary diversification and ~100 via synthetic diversification) (Fig. 1a). To quantify and ensure sequence diversity, we developed an algorithm that calculates the length and identity of the longest shared repeat between every possible pair of sequences in either orientation, termed *L*_*max*_ (Supplementary Fig. 1a)^16^. For compatibility with contemporary protocols for large-scale assembly of synthetic DNA in yeast, our goal was to identify a set of sequences that satisfied the constraint of *L*_*max*_ < 40^27,29^.

For evolutionary diversification, we selected 89 diverse orthologs of human U6 promoters with putative transcriptional activity^33^ from across vertebrate species, the canonical human RNU6–1 promoter that is widely used in mammalian RNAi and gRNA delivery vectors^11,34–37^, 4 mammalian promoters designed for a 3-gRNA array lentiviral Perturb-seq vector^11,34–37^, and finally 3 additional human U6 promoters that were sufficiently divergent from the human RNU6–1 promoter^33^, that as a set satisfied *L*_*max*_ < 40 (n = 97; promoter length range: 249–600 bp, mean length = 475 bp). For synthetic diversification, we used the human RNU6–1 promoter as a starting template, and shuffled nucleotides located in between known core transcription factor binding sites (TFBS), and in a subset of cases, introducing putatively tolerated SNVs into core TFBSs as well as random 3-bp spacers between core TFBS, again ensuring that as a set these satisfied *L*_*max*_ < 40 (n = 112; promoter length range: 249–252 bp, mean length = 250 bp). Applying the *L*_*max*_ algorithm to the combined set of 209 diversified Pol III U6 promoters by checking all 21,736 possible pairwise combinations, we found they continue to satisfy *L*_*max*_ < 40 (Fig. 1b; Supplementary Fig. 1b; Methods; Table S1).

We then sought to perform a multiplex experiment that quantified the relative activity of these Pol III promoters. For this, we cloned the promoters upstream of a pegRNA designed to install a 5 bp insertional barcode at the *HEK3* locus in the human genome, with a strategy that linked each Pol III promoter to a specific barcode (Fig. 1a)^38,39^. In the experiments described below, we quantify the functional activity of a given promoter as the frequency of its insertional barcode at the genomic target site (iBC) normalized by the frequency of the same barcode in the plasmid library encoding the promoter-pegRNA combinations (pBC). We refer to this ratio as the edit score, analogous to regulatory element activity scores of massively parallel reporter assays (Fig. 1a)^39,40^.

To account for the possibility that the barcodes themselves influence pegRNA abundance and/or prime editing efficiency^31^, we also measured the RNA abundance and insertion efficiency of every possible 5N insertion (n = 1,024 barcodes) when driven by the standard human RNU6–1 promoter (Supplementary Fig. 2; Supplementary Fig. 3; Table S2). The biases associated with transcription and editing are overwhelmingly uncorrelated, with the exception of 7 5N variants that contain a “TTTT” polyT Pol III termination sequence, and thus exhibit consistently severe depletion in both transcription and editing data (Supplementary Fig. 3c–e). To correct for barcode bias, we use the relative editing rates of the 5N barcodes from this experiment, which reflect the combined consequences of transcription bias and editing bias, to further normalize the edit scores calculated for individual promoters or scaffolds.

We introduced this library of Pol III promoter-driven pegRNAs to human K562 cells, HEK293T cells, or iPSCs that had been engineered to stably express a prime editor^38,41^. Both synthetically and evolutionarily diversified U6 promoters drove genome editing at the *HEK3* locus at a broad range of levels (Fig. 1c–e; Supplementary Fig. 4; Table S1). Edit scores were reasonably well correlated between technical replicates (*r* = 0.47–0.96) and cellular contexts *(r* = 0.85–0.96; Fig. 1c; Supplementary Fig. 4). Of note, evolutionarily diversified U6 promoters displayed greater variance in activity levels than synthetically diversified alternatives, consistent with their greater sequence divergence from the human RNU6–1 promoter (Fig. 1d; Supplementary Fig. 5). The canonical human RNU6–1 promoter was consistently among the most active promoters, modestly outperformed by only a U6 promoter of *Ornithorhynchus anatinus*, the duck-billed platypus (1.2–1.8-fold; Fig. 1e; Table S1).

Altogether, we identified 146/209 (70%) promoters that drove editing in all 3 cellular contexts (Fig. 1e). There were 70 promoters displaying edit scores > 1 across all contexts, which corresponds to activity within about 50-fold of the standard human RNU6–1 promoter (Table S1). Among these, there were 28 whose activity fell within 5-fold of the standard human RNU6–1p in all three contexts, including all three other human U6 promoters tested^33^, 2/4 promoters previously tested by Adamson et al.^11^, and 23 newly characterized promoters (21 evolutionary diversified, 2 synthetically diversified) (Fig. 1e; Table S1). Four of these 23 highly functional, newly characterized U6 promoters ranked higher than previously characterized non-human RNU6–1p orthologs, specifically those of the common snapping turtle (*Chelydra serpentina*), the one-humped camel (*Camelus dromedarius*), the domestic muscovy duck (*Cairina moschata domestica*), and finally the aforementioned platypus (Fig. 1e; Table S1).

We sought to validate these results using two strategies. First, we identified a subset of the diversified U6 promoters representing a broad range of activity levels in the primary screen and then re-cloned and independently tested them in a monoclonal PEmax-iPSC line (n = 50 diversified U6 promoters together with the standard human RNU6–1p). Results for this validation set correlated strongly with results from the primary screen (*r* = 0.93; Supplementary Fig. 6a). Second, we simultaneously measured transcription scores and edit scores for all 209 diversified promoters using targeted RNA-seq of pegRNA transcripts and our multiplex prime editing functional assay, respectively (Supplementary Fig. 6b). The resulting data was reproducible across transfection replicates (all *r* > 0.97 for edit scores; all *r* > 0.82 for transcription scores). Furthermore, edit scores correlated well with both edit scores from the primary screen (*r* = 0.87) and transcription scores from the validation screen (*r* = 0.83) (Supplementary Fig. 6c–h; Table S3).

Together with the primary screen, these validation experiments confirm synthetically and evolutionarily diversified U6 promoters from across species are functional in human cells and reproducibly exhibit a broad range of activities in driving genome editing. Although both strategies yielded functional promoters with activities within 5-fold of that of human RNU61-p, the vast majority of this highly active subset were evolutionarily diversified. While human RNU6–1p was consistently among the top performers in human cells, there were a few U6 promoters from extant species that exhibited comparable activity in human cells, despite extensive sequence divergence.

### Design, synthesis and functional characterization of diversified pegRNA scaffolds

**Figure 2 |:**
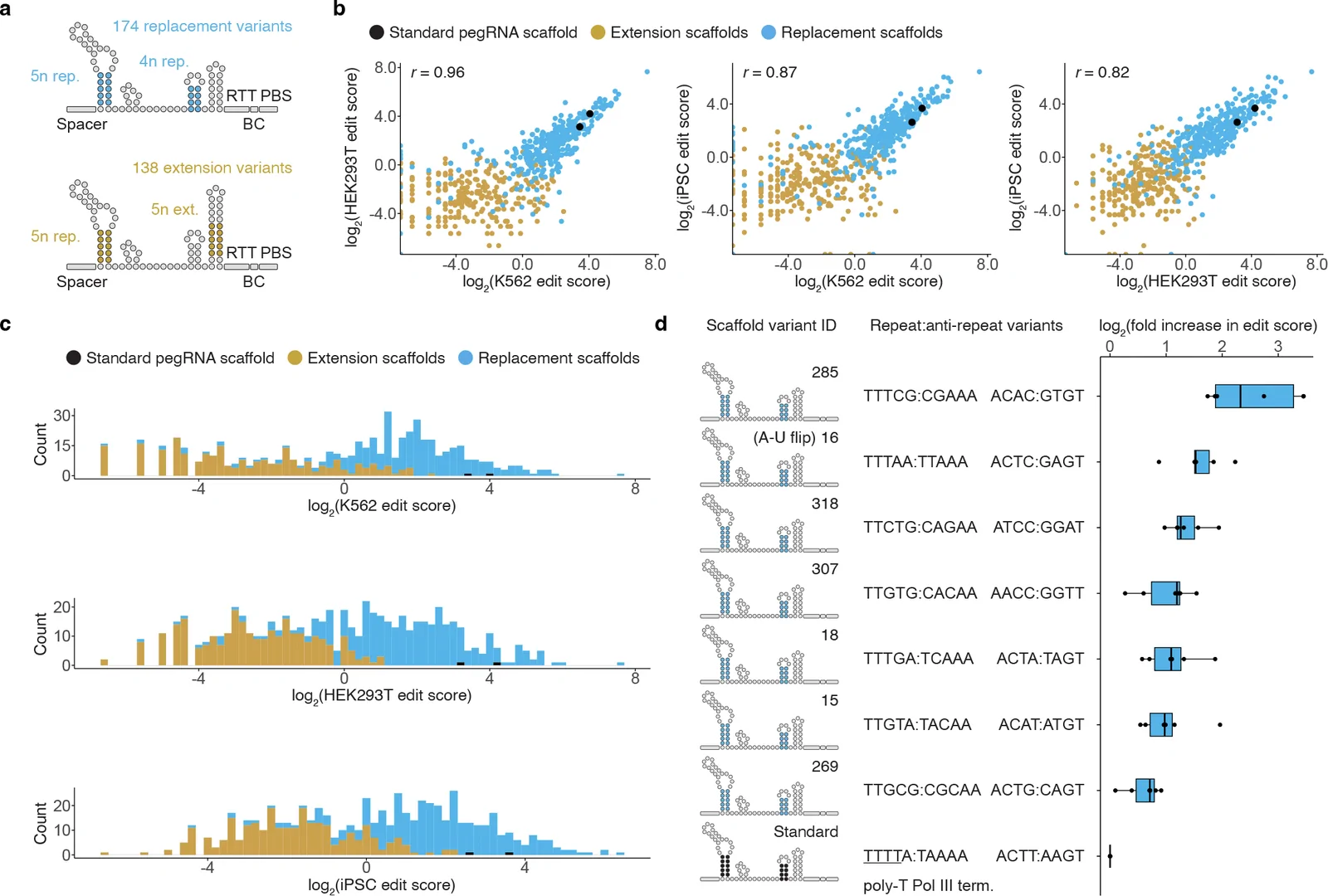
Multiplex functional characterization of diversified pegRNA scaffolds in human cells. **a)** Diversified pegRNA scaffold designs. Complementary R:AR sequences were introduced at specific locations, producing either replacement (top) or extension (bottom) variants of the conventional pegRNA scaffold. **b)** Pairwise comparison of log-transformed edit scores between cellular contexts. Pearson correlation coefficients, calculated on barcode-normalized edit scores prior to log transformation, are listed. **c)** Replacement scaffolds tended to have higher edit scores than extension scaffolds. **d)** Diversified pegRNA scaffolds that eliminated a Pol III termination sequence consistently exhibited higher edit scores than the standard scaffold. Boxes represent the 25th and 75th percentiles, box centre line represents the median. Whiskers extend from hinge to 1.5 times the interquartile range. (n = 3 transfection replicates for each of two separate libraries each with a different iBC per scaffold, these 6 edit scores for each scaffold are shown). Primer Binding Site (PBS), Reverse Transcription Template (RTT).

Diversifying gRNA scaffolds is considerably more challenging than diversifying Pol III promoters due to extensive constraints on gRNA secondary structure^12,16,42–44^. We designed libraries of diversified pegRNA scaffolds to satisfy *L*_*max*_ < 40 using two approaches. First, we introduced putatively secondary structure-retaining 5N and 4N replacements to repeat:anti-repeat (R:AR) regions (“replacement designs”). Second, we introduced 5N insertions to regions predicted to tolerate insertions based on pegRNA secondary structure, along with R:AR 5N replacements (“extension designs”). Altogether, we designed 174 replacement and 138 extension pegRNA scaffolds, and then specific versions of these to install a 5 bp insertional barcode (iBC) at the human *HEK3* locus (Fig. 2a; Supplementary Fig. 7).

We synthesized and cloned these 312 pegRNA scaffold variants downstream of human RNU6–1p, each driving a specific iBC, and introduced them to human K562 cells, HEK293T cells, or iPSCs that stably expressed a prime editor^38,41^. Because the impact of the iBC sequence on pegRNA secondary structure and insertion efficiency can be difficult to predict^39,45^, we also synthesized and cloned each pegRNA scaffold with an alternate iBC in a second library, which was tested independently. After sequencing 5 bp insertional barcodes at the *HEK3* locus, we quantified the edit score for each scaffold-iBC pair and normalized these for differential iBC efficiencies as above (Table S4). Results correlated reasonably well across cellular contexts (*r* = 0.82–0.96; Fig. 2b; Supplementary Fig. 8) as well as across independent iBC sets (*r* = 0.58–0.75; Supplementary Fig. 9). Overall, replacement designs markedly outperformed insertion designs (13- to 37-fold higher median edit score across cellular contexts; Fig. 2c).

Altogether, we identified 272/312 (87%) pegRNA scaffolds that drove editing with both iBCs across all cellular contexts (Table S4). Among these, 58 functioned within 5-fold of the standard pegRNA scaffold with both iBCs across all cellular contexts, including seven that outperformed the standard pegRNA scaffold (Fig. 2d; Table S4). These seven included a scaffold with a previously described A-U flip design that swaps nucleotides in the first R:AR region to remove a polythymidine Pol III termination sequence (“TTTTA:TAAAA”>”TTTAA:TTAAA”), previously reported to improve function by reducing premature termination of Pol III transcription^13,46^. The remaining six scaffolds that outperformed the standard pegRNA each maintain the first two “TT” nucleotides in the first R:AR sequence while introducing variants that disrupt the Pol III termination sequence through means other than the A-U flip (Fig. 2d). Taken together, these results identify dozens of sequence-diversified pegRNA scaffolds that are similarly active to the conventional scaffold in human cells, and confirm two strategies to diversify (pe)gRNA scaffolds while maintaining or improving their function: namely introducing complementary R:AR variants and/or removing Pol III termination sequences.

### Saturation mutagenesis and functional assessment of a miniaturized U6p-pegRNA cassette

The diversified parts described thus far were designed to satisfy *L*_*max*_ < 40, a practical requirement for yeast-based assembly of large constructs^27,29^. Smaller subsets of parts can be selected from these libraries to further increase diversity. However, more comprehensive knowledge regarding which variants can be introduced to a Pol III promoter and/or gRNA scaffold while retaining functionality would enable design of further diversified parts to satisfy even more stringent *L*_*max*_ requirements. To this end, we conducted saturation mutagenesis and functional assessment of a U6p-pegRNA cassette.

**Figure 3 |:**
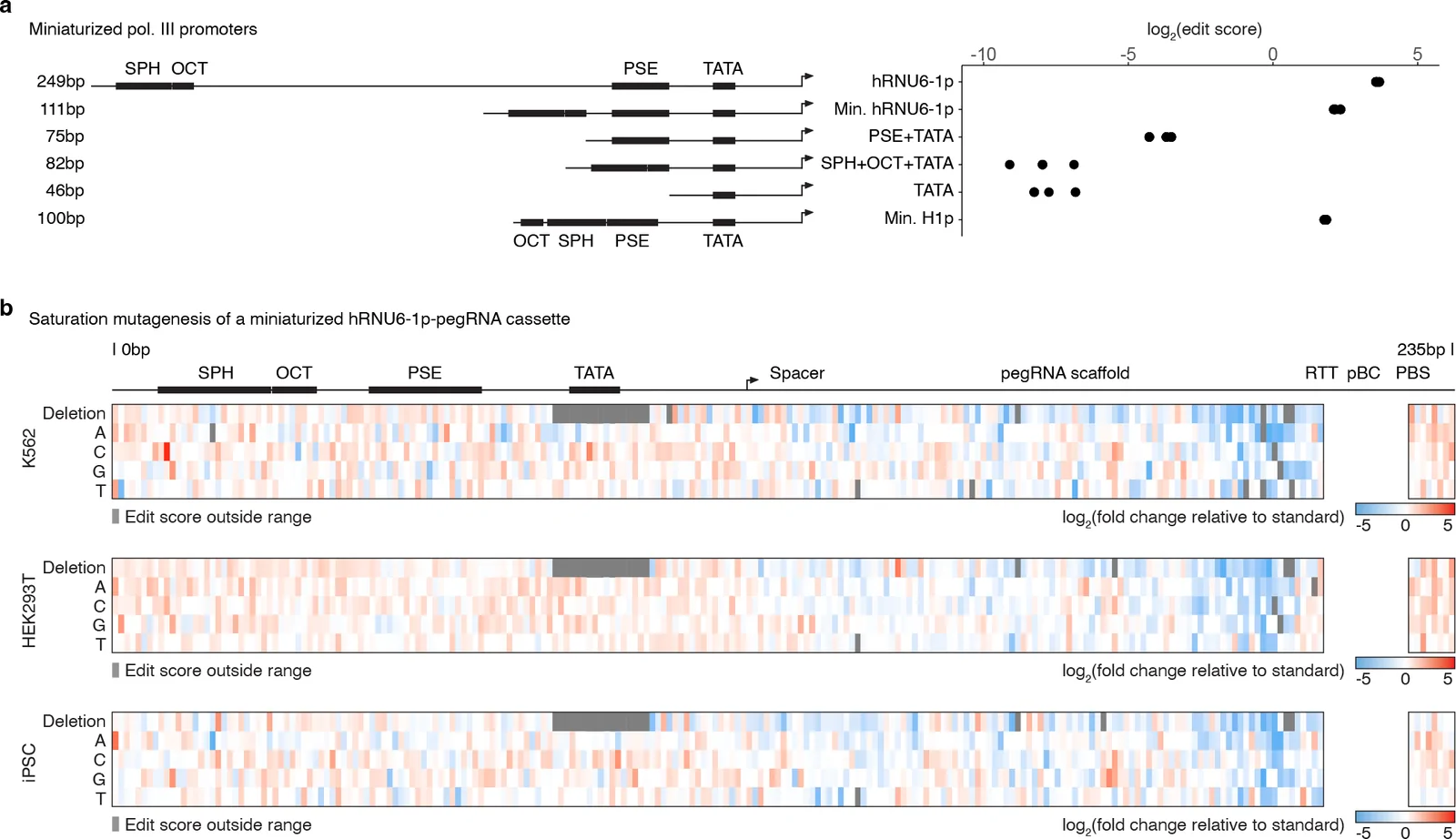
Saturation mutagenesis of a miniaturized U6p-pegRNA cassette. **a)** Left: Human Pol III promoter deletion series constructs and corresponding lengths. Locations of key TFBS are labeled. The top five rows correspond to hRNU6–1p and miniaturized variants thereof. The key TFBS are always in the same order from 5’ to 3’ (5’-SPH-OCT-PSE-TATA). The bottom row corresponds to the 100 bp human H1 promoter, in which the positions of the OCT and SPH elements are reversed relative to hRNU6–1p. Right: Log-scaled edit scores of wildtype or miniaturized Pol III promoters (n = 3 transfection replicates each with 4 iBCs per promoter, mean of the edit scores of these 4 iBCs per transfection replicate are shown). **b)** Variant effect maps of saturation mutagenesis of a miniaturized hRNU6–1p-pegRNA cassette tested across three human cellular contexts. Color-scaled, log-transformed fold-changes in median edit scores relative to minU6p-pegRNA are shown. Edit scores were not calculated for the unboxed region surrounding the pBC, as exact matches spanning this region were required for edit quantification.

To focus our efforts on the most critical sequence elements, our “wild-type” construct appends a miniaturized version of the canonical human RNU6–1 promoter^47^ that retains its four key TFBS while deleting divergent intervening regions (shortened from 249 to 111 bp; Fig. 3a; Table S5) to a standard pegRNA driving a 5 bp insertion (124 bp). We first sought to confirm that the wild-type version of this 235 bp minU6p-pegRNA cassette is functional, and found it drove editing at 38% of standard hRNU6–1p levels (Fig. 3a). In contrast, deletion of TFBS from minU6p severely diminished activity (169- to 2732-fold reduction; Fig. 3a). The H1 promoter, a naturally occurring human Pol III promoter, similarly miniaturized in the sense that the TFBS are retained, exhibited similar activity as miniaturized U6p (29% of standard hRNU6–1p; Fig. 3a). Taken together, these results confirm that retention of TFBS while deleting divergent intervening sequences is a general approach for deriving miniaturized Pol III promoters that retain function^47,48^.

With the wild-type miniaturized U6p-pegRNA as the baseline, we designed, synthesized and cloned two libraries encoding every possible single nucleotide substitution and single nucleotide deletion across its length (230 bp excluding the 5N iBC; n = 920 variants in total; a second library is identical but with a different set of iBC pairings; Table S5). We then, as above, introduced these libraries to three human cellular contexts and quantified edit scores. These experiments revealed a biologically coherent landscape of variant effects with consistent sequence-function relationships across cellular contexts (Fig. 3b; Supplementary Figs. 10–11). As expected given the flexibility of the *cis*-regulatory code, the U6 promoter region (positions 1–111) was more tolerant to variation than the pegRNA (positions 112–235; 1.6- to 1.9-fold higher median edit score across cell contexts; Fig. 3b; Supplementary Fig. 11). Single nucleotide deletions within the U6 promoter TATA box (positions 81–89: “TTTATATAT”) were not tolerated (Fig. 3b). Activity was also particularly compromised by deletions in the nucleotides forming the final pegRNA stem loop (positions 198–202: “GAGTC”; 2.1- to 5.4-fold lower edit scores than all other deletions) or PAM-proximal portion region of the spacer (positions 122–131: “GAGCACGTGA”; 1.4- to 1.6-fold lower edit scores than all other deletions; Fig. 3b; Supplementary Fig. 11). These results are consistent with the core roles of these elements in the editing cycle of a pegRNA: transcription, stability, and target nicking, respectively.

In contrast to single nucleotide deletions, many SNVs were tolerated throughout the length of the cassette, and several displayed enhanced performance compared to the miniaturized U6p-pegRNA cassette (Fig. 3b; Table S5). In particular, 16 of 920 variants, 15 of which were SNVs, displayed increased edit scores across both iBCs in all three cellular contexts (median 1.9-fold higher edit scores, max 20.8-fold; Fig. 3b; Table S5). 13/16 (81%) of these variants were in the miniaturized promoter, of which 5 introduced substitutions to a “TATT” sequence at the end of the proximal sequence element (PSE; positions 64–67), which may boost function by improving promoter conformation and/or transcription initiation from the immediately downstream TATA box. The 3/16 (19%) variants with improved function in the pegRNA region all introduced substitutions to two neighboring nucleotides near the 3’ end of the primer binding site (231G>C; 232T>C; 232T>A), suggesting these variants may yield a more optimal primer and/or more stable pegRNA. Relaxing these criteria, we identified 499 variants that functioned within 5-fold of the wild-type minU6p-pegRNA cassette across barcodes and contexts, and 764 that functioned within 50-fold. These results provide a rich set of enhancing or tolerated single nucleotide variants that can be leveraged to boost sequence diversity as needed (Fig. 3b; Table S5).

### Diversified U6 promoters exhibit consistent functional activities in mouse embryonic stem cells

To assess whether the activities of these parts are human-specific or consistent across mammalian models, we next sought to characterize them in mouse embryonic stem cells (mESCs). As mESCs lack an endogenous *HEK3* locus, we introduced synthetic human *HEK3* target sites^49^ (*synHEK3*) and PEmax via piggyBac transposition at a high multiplicity of integration, and isolated a monoclonal line with an estimated 87 *synHEK3* targets (29 integrations × 3 *synHEK3* targets per integration; Supplementary Fig. 12). We then introduced the original library of evolutionarily or synthetically diversified U6 promoters (n = 209) to this cell line and quantified edit scores as above.

As in human cells, diversified U6 promoters drove prime editing in mESCs with very high correlation between technical replicates (r > 0.99; Supplementary Figs. 12–13). We speculated that this high reproducibility was due to the much larger number of *synHEK3* sites in these engineered mouse cells than endogenous *HEK3* sites in human cell lines (~87 vs. 2–3), which is expected to decrease measurement noise. To confirm this, we generated a new monoclonal HEK293T line harboring ~146 *synHEK3* target sites and retested the library of 209 diversified U6 promoters. As in mESCs, we observed that introducing many *synHEK3* target sites resulted in much higher replicate correlations in human cells as well (*r* = 0.96–0.98; compare HEK293T results in Supplementary Figs. 13–14 to those in Supplementary Fig. 4).

Furthermore, results also correlated well between human and mouse cells (*r* = 0.73–0.80; Supplementary Figs. 12–13; Table S1). In mESCs as in human cells, evolutionarily diversified U6 promoters exhibited greater variance in activity (Supplementary Figs. 12–13). The human RNU6–1 promoter was again among the top performing promoters in mESCs, consistently outperforming a commonly used, modified mouse U6 promoter^11,50,51^ as well as another mouse U6 promoter that was part of the evolutionarily diversified set (Supplementary Figs. 12–13; Table S1). Other evolutionarily diversified promoters that were among the most highly active in the human context were similarly highly active in the mouse context (Supplementary Figs. 12–13).

Taken together, these results suggest these diversified U6 promoters can likely be used across both human and mouse model systems with the expectation that their activities will be similar to those observed in human cell lines.

### Testing thousands of ancestral, extant, and mutagenized sequences reveals highly active Pol III promoters for mammalian genome editing

**Figure 4 |:**
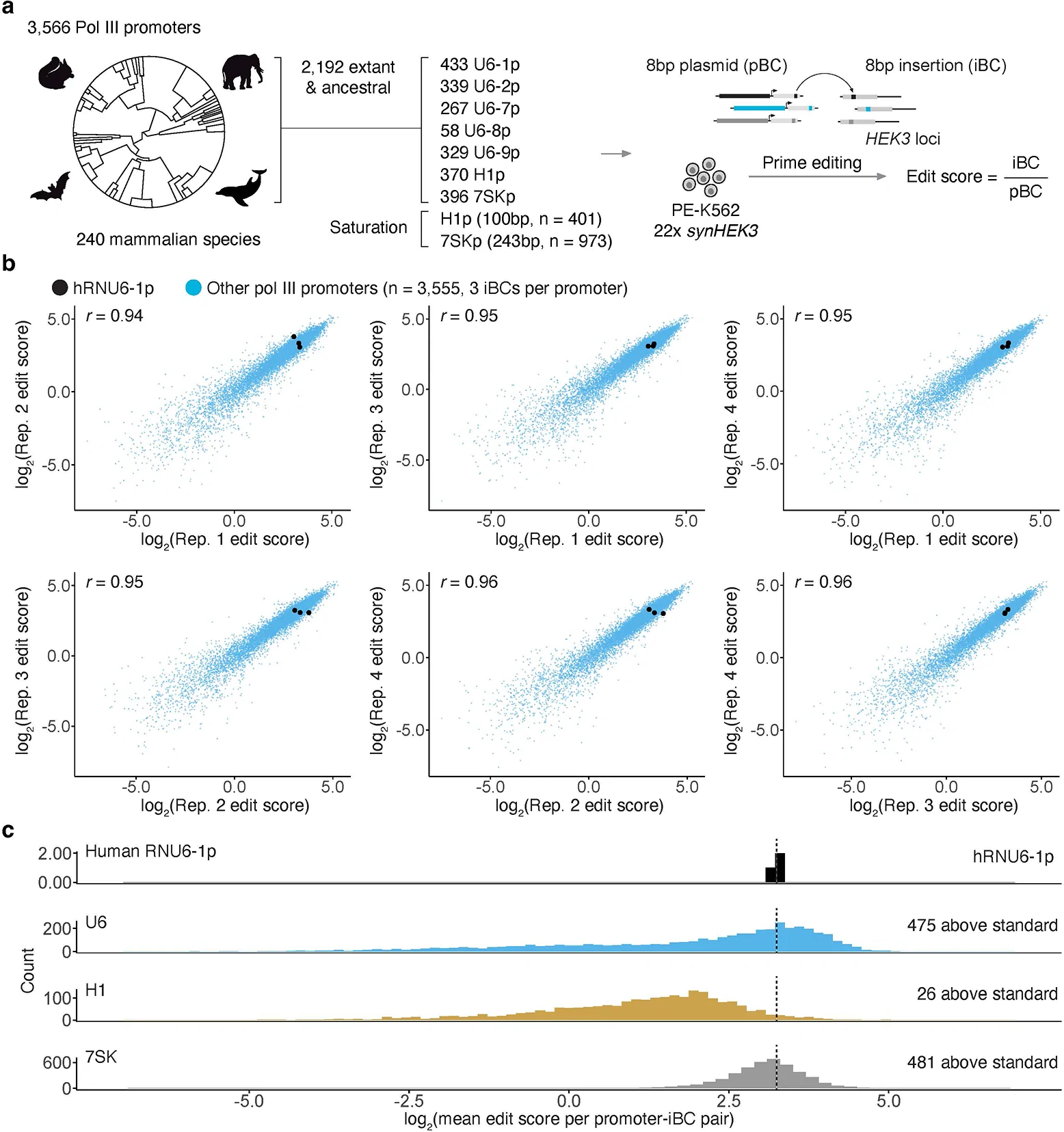
Testing thousands of ancestral, extant, and mutagenized sequences reveals highly active Pol III promoters for genome editing in mammalian cells. **a)** Library design, contents, and multiplex prime editing functional assessment workflow. **b)** Edit scores correlations across the four transfection replicates. Points represent edit scores for the 3 independent iBCs paired with each of the 3,566 promoters (10,698 constructs total). Pearson correlations, calculated on barcode-normalized edit scores prior to log transformation, are shown. **c)** Edit score distributions for the different promoter classes tested in this experiment. The standard human RNU6–1 promoter is shown in the top row, and its mean activity marked with a vertical dashed line.

We next sought to scale both our evolutionary and synthetic approaches to further expand the set of sequence- and activity-diversified Pol III promoters available for use in synthetic biology and genome engineering. Functional candidate parts for genome engineering can be mined from not only extant but also ancestral genomes, *e.g.* as has been done for cytidine deaminases^52^. We leveraged the Zoonomia Project's 240-species Cactus genome alignment^53–55^ to identify extant and ancestral orthologs of seven Pol III promoters known to be functional in mammalian cells (RNU6–1, RNU6–2, RNU6–7, RNU6–8, RNU6–9, H1, and 7SK promoters). Altogether, we extracted 2,192 unique Pol III promoter sequences, including 1,084 that exactly match at least one extant genome, and 1,108 that solely occur in inferred, ancestral genome(s). We supplemented these mammalian Pol III promoters with saturation mutagenesis libraries encompassing all single nucleotide substitutions and deletions of the human H1 (100 bp, 401 variants including wildtype) and 7SK (243 bp, 973 variants including wildtype) promoters. Altogether, this library contained 3,566 ancestral, extant or mutagenized mammalian Pol III promoters (Fig. 4a).

To facilitate accurate quantification of the relative activities of these promoters, we leveraged insights from earlier experiments. First, given the high technical reproducibility of multiplex prime editing experiments conducted in monoclonal mESCs and HEK293Ts with large numbers of *synHEK3* target sites (*r* > 0.99; Supplementary Figs. 12–14), we used a monoclonal K562 line with 22 *synHEK3* targets^49^ and PEmax^41^ as our prime editor for these experiments (Fig. 4a). Second, we paired each Pol III promoter with 3 independent iBCs (3,566 promoters × 3 iBCs = 10,698 constructs total), accommodating the larger library size by switching from a 5 to 8 bp barcode. To facilitate downstream normalization, we measured the relative insertion activity of all 65,536 possible 8N insertions when driven by the same hRNU6–1p promoter (Supplementary Fig. 15; Table S6).

Following transfection and *synHEK3* amplicon sequencing, we observed the expected insertional edits with strong concordance in edit scores derived from four transfection replicates (*r* > 0.94; Fig. 4b). We also observed strong correlation across the three independent iBCs associated with each Pol III promoter *(r* > 0.80; Supplementary Fig. 16). This correlation was markedly improved by correcting for relative barcode insertion efficiency (r = 0.48–0.51 before vs. 0.80–0.81 after barcode correction; Supplementary Fig. 16). This result reinforces the importance of having relative activity measurements for all iBCs used, particularly for longer iBCs, which exerted greater influence on raw edit scores than shorter barcodes (Supplementary Fig. 2; Supplementary Fig. 3; Supplementary Fig. 13).

Global analyses of this screen revealed a broad range of mammalian Pol III promoter activity levels, with clear differences between the activity distributions of the classes of elements tested. Evolutionary orthologs of the H1 promoter exhibited weaker activity than orthologs of U6 or 7SK promoters (Fig. 4c), consistent with our earlier comparisons of the short H1 and miniaturized U6 promoters vs. the full length U6 promoter (Fig. 3a). Also consistent with expectation, saturation mutagenesis of the human H1 and 7SK promoters highlighted the four core TFBS as particularly constrained, while also identifying numerous tolerated and activity-enhancing SNVs which could be leveraged for additional diversification (Supplementary Fig. 17). Notably, as compared with U6, the H1 and 7SK Pol III promoters were much more tolerant of single nucleotide deletions in their TATA boxes, but much less tolerant of mutations in the SPH or PSE elements (Fig. 3b; Supplementary Fig. 17).

As in earlier screens, hRNU6–1p was among the most highly active promoters (Fig. 4c). Remarkably however, we also identified 982 promoters that outperformed hRNU6–1p across all iBCs (982/3566 or 28%, including 475 U6, 26 H1, and 481 7SK promoter orthologs; median 1.3-fold increase over hRNU6–1p; Fig. 4c; Table S7). 408/982 (42%) of these hRNU6–1p outperformers were not present in any extant mammalian genome in the Zoomania Project, highlighting the potential value of inferred, ancestral genome(s) as a source of noncoding regulatory parts for synthetic biology. These included the most active Pol III promoter in this experiment, a 7SK promoter ortholog from an intermediate ancestral rodent genome that drove prime editing at *synHEK3* sites with 2.6-fold greater activity than hRNU6–1p. Other top-performers derived from saturation mutagenesis (25%) or extant genomes (33%), the latter including Pol III promoters from the genomes of the java mouse deer (*Tragulus javanicus*), long-tongued fruit bat (*Macroglossus sobrinus*), Linnaeus's two-toed sloth (*Choloepus didactylus*), and one of our closest relatives, the bonobo (*Pan paniscus*) (Table S7).

We next sought to validate results for these 3,566 promoters by conducting a full replication experiment with simultaneous genome editing and transcription measurements (Supplementary Fig. 18a). The resulting data was reproducible across transfection replicates (all *r* > 0.9 for edit scores; all *r* > 0.86 for transcription scores). Furthermore, edit scores correlated well with both edit scores from the primary screen (*r* = 0.96) and transcription scores from the validation screen (*r* = 0.74) (Supplementary Fig. 18b–g; Table S8). These results provide further confidence in the estimated activity levels of these 3,566 diversified Pol III promoters.

While our main goal was to generate diversified parts to facilitate genome engineering, synthetic biology, and molecular recording, this experiment incidentally mapped the distribution of activities of ancestral and extant orthologs of Pol III across the mammalian phylogeny (Supplementary Fig. 19). For example, at least when assayed in human cells, hRNU6–9p orthologs from primates are more active than hRNU6–9p orthologs from other orders (FDR < 0.1), while hRNU6–1p orthologs are not (Supplementary Fig. 20). Further investigation of such patterns with phylogenetic methods has the potential to shed light on the evolution of Pol III promoter sequences.

We suspect that the much higher proportion of Pol III promoters whose activities exceed hRNU6–1p in this screen, as compared with the primary screen, follows from sampling an order of magnitude more sequences from more closely related species, with less attention to ensuring their sequence divergence. Alternatively, this may stem from modest overestimation of hRNU6–1p activity in earlier, single barcode screens (see further validations below, which support this interpretation). Nonetheless, this set is sufficiently large so as to enable the selection of subsets that are highly sequence-diverse so as to facilitate yeast-based assembly. For example, of the 481,687 possible pairwise comparisons among the 982 Pol III promoters that outperformed hRNU6–1p, there exist subsets of at least 205 that satisfy *L*_*max*_ < 40 (Supplementary Fig. 21). This effectively provides a large set of yeast-assembly-compatible Pol III promoters that are as or more active than hRNU6–1p for driving genome editing.

### Validation of diversified Pol III promoters and gRNA scaffolds at additional target loci identifies parts that consistently outperform the standard components

**Figure 5 |:**
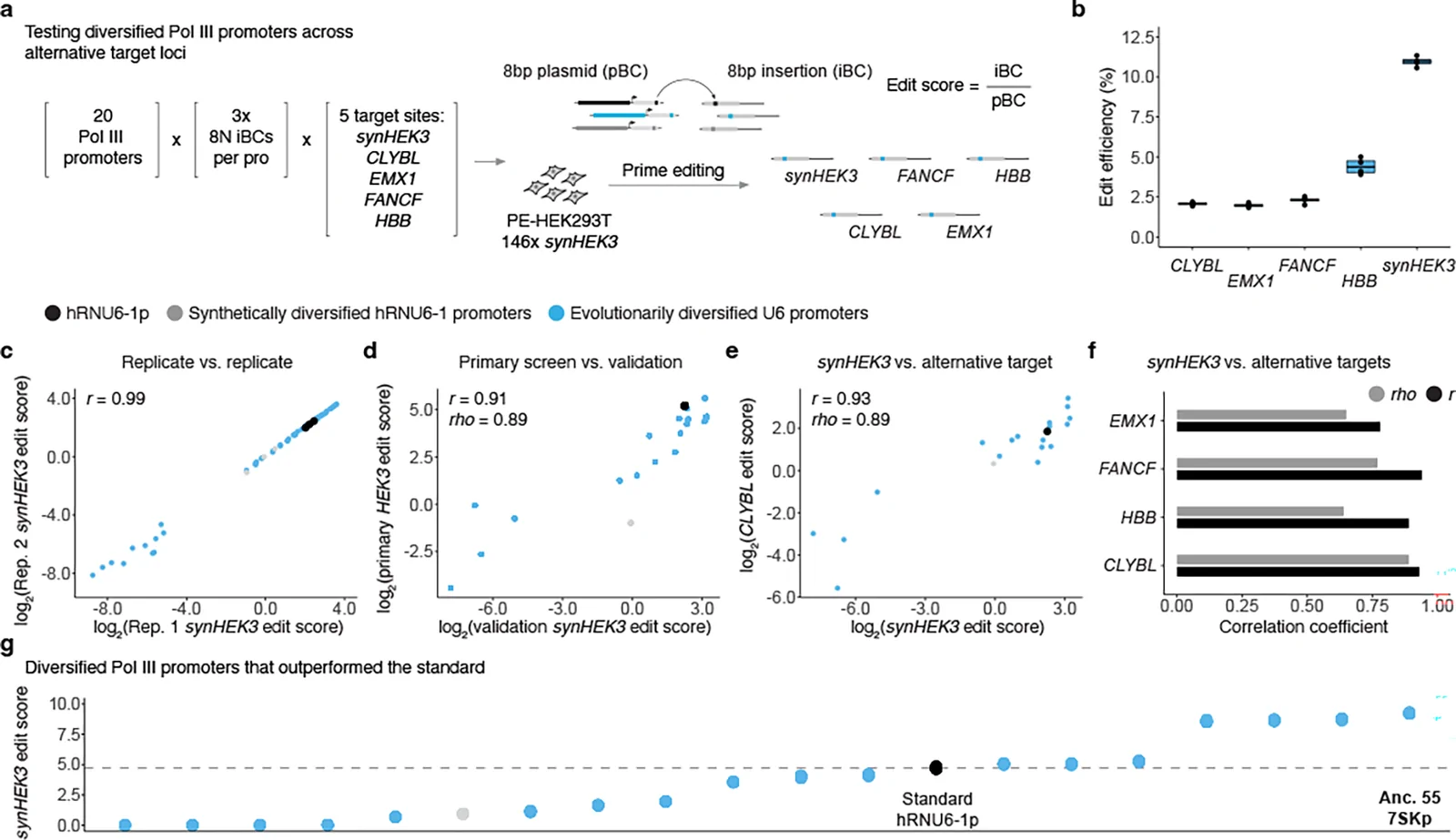
Validation of diversified Pol III promoters at additional target loci. **a)** Library design, contents, and multiplex prime editing functional assessment workflow. **b)** Diversified Pol III promoters drove editing across all tested target loci: *CLYBL*, *EMX1*, *FANCF*, H*BB*, *synHEK3*. Editing efficiencies, calculated as the percentage of reads with programmed 8 bp insertions at each locus for each transfection replicate (n = 4), are shown. Boxes represent the 25th and 75th percentiles, box centre line represents the median. Whiskers extend from hinge to 1.5 times the interquartile range. **c)** Reproducibility of edit scores between transfection replicates for *synHEK3* target sites. Pearson correlation coefficients calculated on edit scores for each construct prior to log transformation, are listed. **d)** Reproducibility of edit scores from the primary screen vs. this validation screen. Pearson and Spearman correlation coefficients, calculated on edit scores prior to log transformation, are listed. **e)** Comparison of edit scores at *synHEK3* vs. exemplary alternative target locus, *CLYBL*. Pearson and Spearman correlation coefficients, calculated between log-transformed edit scores, are listed. **f)** Barplot of Pearson and Spearman correlation coefficients, calculated between log-transformed edit scores, between *synHEK3* and alternative target loci. **g)** Diversified Pol III promoter edit scores at *synHEK3*. Four points are plotted for each of 20 promoters (*x-*axis), each representing mean promoter edit scores across 3 8N iBCs for one transfection replicate (points are overlapping due to high reproducibility, such that they are not visually distinguishable). The ancestral rodent 7SK promoter was the top-performing promoter in both the primary screen and cross-locus validations.

We next sought to validate diversified Pol III promoters and gRNA scaffolds at additional genomic target loci. First, we selected 20 diversified Pol III promoters that exhibited a broad range of activity levels in the primary (n = 209) or scaled (n = 3,566) screens, including hRNU6–1p. We paired each of these 20 promoters with 3 pegRNAs designed to install unique 8N iBCs at each of five distinct genomic target loci: *CLYBL*, *EMX1*, *FANCF*, *HBB* and *synHEK3* (20 promoters × 3 8N iBCs × 5 target loci = 300 constructs) (Fig 5a). Second, we took all 313 gRNA scaffold designs and reprogrammed them to install 3 unique 8N iBCs at the same five target loci. We supplemented these with an additional 100 new gRNA scaffold variants that preserve the transcription-enhancing A-U flip variant while introducing additional diversifying R:AR replacement variants (413 scaffolds × 3 8N iBCs × 5 target loci = 6,195 constructs; Supplementary Fig. 22a).

We introduced these libraries into a monoclonal HEK293T line expressing PEmax and bearing ~146 randomly integrated *synHEK3* target sites. After three days, we independently amplified each endogenous target locus, or all *synHEK3* sites, and quantified edit scores. Diversified promoters and scaffolds successfully drove editing at all five target loci (Fig. 5b; Supplementary Fig. 22b). As expected based on our earlier screens, edit scores at *synHEK3* correlated exceptionally well across transfection replicates for both diversified Pol III promoters (*r* > 0.99; Fig. 5c; Supplementary Fig. 23a) and gRNA scaffolds (*r* > 0.99; Supplementary Fig. 22c; Supplementary Fig. 24a). At single copy endogenous target loci, edit scores also correlated reasonably well across transfection replicates for both Pol III promoters (*CLYBL r* = 0.87–0.92; *EMX1 r* = 0.86–0.94; *HBB r* = 0.91–0.98; *FANCF r* > 0.99) and gRNA scaffolds (*CLYBL r* = 0.63–0.74; *EMX1 r* = 0.45–0.66; *HBB r* = 0.61–0.79; *FANCF r* = 0.83–0.89). The more modest reproducibility at alternative endogenous sites than we observed for endogenous *HEK3* is likely due to a combination of sparse measurements for poorly active scaffolds and target-specific differences in iBC insertion efficiencies (*i.e.* we did not measure baseline efficiencies for all 65,536 8N iBCs at these alternative endogenous loci as we did for *HEK3/synHEK3*).

Are the activities of parts at one genomic location or target site predictive of their activities at another? For the former (generalizability across genomic locations), we compared results from endogenous *HEK3* (primary screen) vs. *synHEK3* sites (validation screen) and found them to be highly correlated (promoters, *r* = 0.91; scaffolds, *r* = 0.87; Fig. 5d; Supplementary Fig. 22; Tables S9–S10). For the latter (generalizability across target sites), we compared results from *synHEK3 (*validation screen) vs. alternative endogenous loci (validation screen) and also found them to be reasonably well correlated (promoters, *r* = 0.79–0.93; scaffolds, *r* = 0.43–0.60; Fig. 5e–f; Supplementary Fig. 22e–f; Tables S9–S10), despite the lack of target site-specific iBC edit score normalization at alternative targets. Once again, these correlations were more modest for diversified gRNA scaffolds, plausibly due to the greater opportunity for interaction between the iBC and/or target sequence with variable scaffold sequences (*i.e.* spacer, primer binding site (PBS), and reverse-transcription template (RTT)). Nonetheless, classes of gRNA scaffolds exhibited consistent patterns of activity across target loci, *e.g.* extensions exhibiting lower activity than both replacements and A-U flip variants (Supplementary Fig. 22g).

This screen also revealed promoters and gRNA scaffolds that consistently outperformed the standard components. For scaffolds, this included 17 designs that outperformed the standard across all target genomic loci, all of which were replacement or A-U flip variants (Supplementary Fig. 22; Table S10). Notably, these included 6/7 of the scaffolds that outperformed the standard scaffold in the primary screen at endogenous *HEK3* (Fig. 2d). The sole exception was scaffold 285, which outperformed the standard scaffold at all loci except *HBB* (Table S10).

For promoters, even though in our validation experiments we focused on a few Pol III promoters exhibiting a broad range of activities in the primary screen, 7/20 outperformed the standard at *synHEK3* (Fig. 5g), and 4/20 across all five target loci (Table S9). Notably, these included the ancestral rodent 7SKp promoter that was the top-performing promoter both here as well as in our scaled screen of 3,566 promoters (Fig. 4; Fig. 5g).

Taken together, these results show that the activities of diversified Pol III promoters and gRNA scaffold parts at *HEK3* are predictive of their activities at other target sequences and genomic locations. Furthermore, they highlight several Pol III promoters and gRNA scaffolds that consistently exhibit higher levels of activity than the standard parts.

### Single-step assembly and deployment of a “ten key” diversified molecular recording array

**Figure 6 |:**
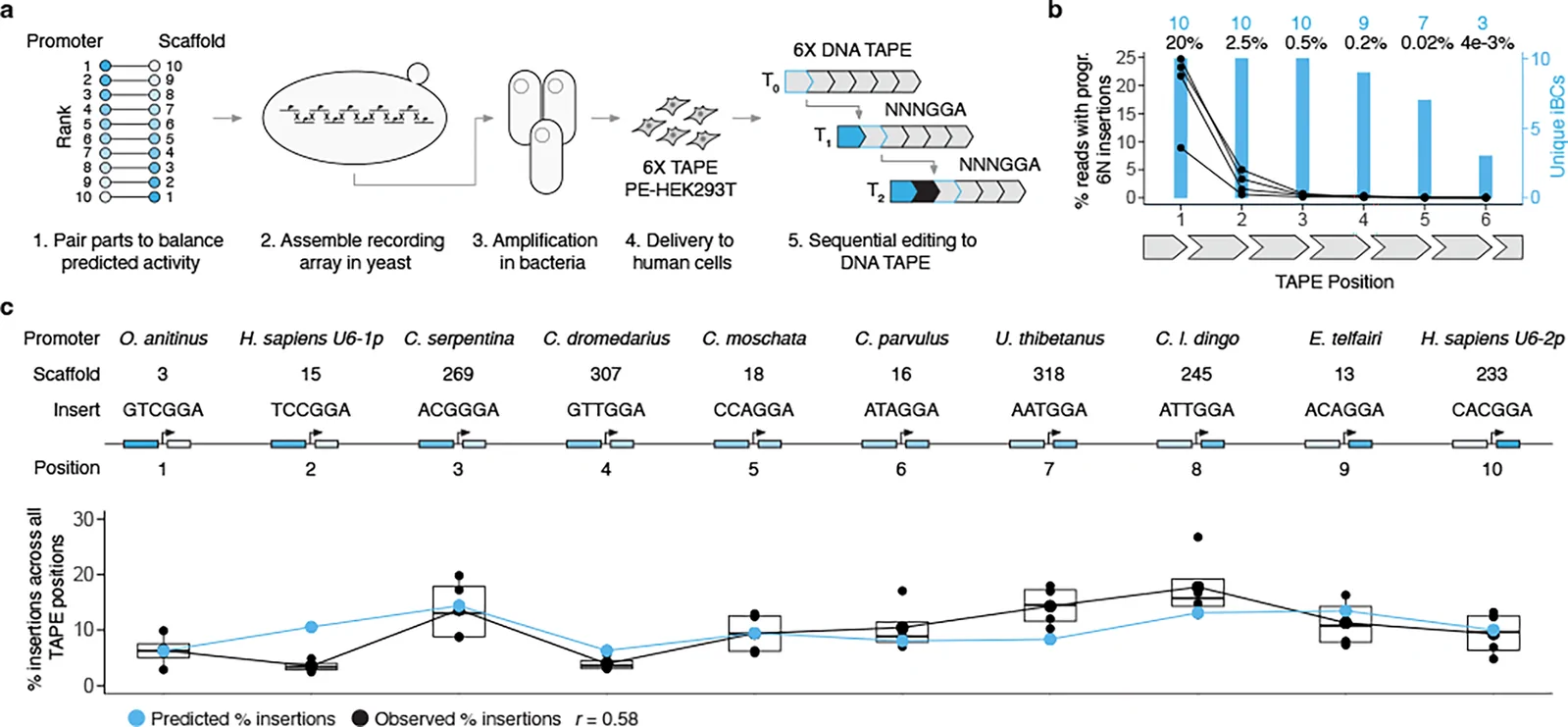
Single-step assembly and functional testing of a “ten key” diversified molecular recording array. **a)** Schematic of workflow. 1) Diversified parts were paired in reverse rank order based on individual part activity measurements to balance predicted activity levels. 2) Diversified U6p-pegRNA-iBC units were one-step assembled in yeast. 3) The assembly was recovered, sequence validated and amplified in bacteria. 4) The assembly was delivered to mammalian cells for sequential recording with DNA Typewriter. 5) Each insertion of an NNNGGA barcode (*i.e.* NNN as iBC; GGA to complete the next target site for sequential editing). **b)** Editing efficiency and number of unique iBCs recovered at each of the six sequential sites in DNA Tape. Each dot represents an individual transfection replicate (n = 4). Higher editing rates in earlier sites are expected due to sequential editing by DNA Typewriter. **c)** Proportion of insertions derived from each of the 10 units of the diversified recording array across all DNA Tape sites. Observed proportions are correlated with predicted editing rates for each U6p-pegRNA-iBC unit. Smaller dots represent individual transfection replicates (n = 4), larger dots represent mean of transfection replicates or predicted editing rates. Boxes represent the 25th and 75th percentiles, box centre line represents the median. Whiskers extend from hinge to 1.5 times the interquartile range.

With functional parts in hand, we sought to test whether these parts were sufficiently sequence-diverse to enable their one-step assembly in yeast, and to then deploy this assembly in mammalian cells. In addition, we sought to assess whether activity measurements for isolated Pol III promoters, scaffolds, and iBCs could be used to predict the activity of U6p-pegRNA-iBC combinations, as well as the relative activity of multiple U6p-pegRNA-iBC units assembled into a large array. For this, we designed a ten-unit array of “keys” based on our diversified parts and DNA Typewriter^31^, a time-resolved, multi-symbol molecular recording system that relies on sequential prime editing (Fig. 6a). In brief, DNA Typewriter leverages a “Tape” composed of a tandem array of prime editing target sites, most of which lack the first 3 bp of the spacer targeted by corresponding pegRNAs, with the exception of the 5’-most site, which is complete. Each sequential round of prime editing inserts a barcode that both records information and completes the next spacer along the tandem array, enabling it to be written to during the next round of prime editing (Fig. 6a). Sequential records generated with DNA Typewriter can be used to reconstruct cellular event histories, *e.g.* of cell lineage^31,39^. In this analogy, pegRNAs encoding different barcodes are analogous to keys on a typewriter, encoding symbols that are written sequentially to media.

In designing this diversified molecular recording array, we sought to balance the activity levels of individual U6p-pegRNA-iBC units, as this is expected to yield a greater diversity of sequential editing patterns and thereby maximize the information content of any resulting recordings. Specifically, we paired 10 of our top promoters with 10 of our top scaffolds (Fig. 6a). Further, we paired each U6p-pegRNA unit with specific “NNNGGA” DNA Typewriter barcodes with similar activity levels^31^. We ordered 494–573 bp sequences corresponding to these ten U6p-pegRNA-iBC units flanked by Versatile Genetic Assembly System (VEGAS) adapters^56^ to facilitate their assembly in yeast (Supplementary Fig. 25). Additional components of the overall design included piggyBac inverted terminal repeats (for random integration), *Bxb1* attB sites (for site-specific integration), orthogonal restriction enzymes sites (for isolation of individual units or the entire array), and flanking anti-repressor-elements (for insulation)^57,58^ (Supplementary Fig. 25). Following the pooled transformation of 14 fragments to yeast (10 U6p-pegRNA-iBC units, four auxiliary and backbone components), we successfully recovered the complete 15.8 kb ten-unit assembly (Supplementary Fig. 25). Whole-construct sequencing revealed only one single nucleotide substitution error that fell at the 5’ end of one of the U6 promoters, upstream of the four core TFBS.

To more formally assess the value of diversified parts in this context, we attempted to construct a similar 10-key recording loci using fully repetitive standard parts - specifically 10 repeats of the standard hRNU61-p and gRNA scaffold (each driving 10 different iBCs). We transformed either the diversified fragments or repetitive fragments into yeast in parallel, using the same set of VEGAS adaptors. We then performed shotgun genomic long-read sequencing on a pool of transformed yeast. Focusing alignments to the intended assembly, the number of successfully assembled junctions per read was markedly higher for assembly with diversified parts than repetitive parts, consistent with expectation (Supplementary Fig. 26a). Furthermore, we only identified reads harboring all 9 assembly junctions when using diversified parts (5 / 346 reads with diversified parts (1.5%) vs. 0 / 430 reads with repetitive parts (0%)) (Supplementary Fig. 26b). These results confirm and quantify the necessity of diversified parts for enabling the yeast-based assembly of arrays of Pol III-driven guide RNAs.

Next, we delivered the diversified “ten key” DNA Typewriter construct to a HEK293T cell line expressing PEmax and multiple integrated copies of a synthetic DNA Tape construct, each with six editable sites for sequential recording (Fig. 6a). After 72 hrs, we observed all or a subset of the ten expected NNNGGA barcodes at each of the six sites, at rates that progressively decreased from the first to sixth unit, consistent with sequential editing (Fig. 6b). Notably, we observed insertions corresponding to all 10 U6p-pegRNA-iBC units, and the proportion of edited reads corresponding to each unit was balanced within a few fold at each DNA Tape site where all 10 iBCs were observed (4.7-fold range; Fig. 6c; Table S11). Further, the proportion of edited reads for each unit predicted by a simple Pol III × scaffold × iBC model based on our individual part measurements mirrored their observed activities throughout the length of the tandem array, with no obvious systematic bias attributable to the 5’ → 3’ position of the U6p-pegRNA-iBC units (Fig. 6c; *r* = 0.58; Table S11). Of note, unit 2, which is an outlier in this correlation, has hRNU6–1p as its promoter, which is consistent with a modest overestimation of the hRNU6–1p in the primary, single barcode screen (see above). Taken together, these experiments confirm that our diversified parts are amenable to large-scale assembly in yeast, and that we can predict the activity of Pol III promoter-gRNA scaffold-iBC combinations (and tandem arrays thereof) based on the measured activities of individual parts.

## Discussion

Here we report sequence-diversified and miniaturized parts for multiplex CRISPR-based genome engineering in mammalian cells. These parts exhibit consistent performance across multiple cell contexts, including the workhorses of functional genomics technology development (HEK293T, K562) and the starting points for diverse organoid and *in vivo* models (human iPSCs, mouse ESCs). Parts in each class (Pol III promoters, guide RNA scaffolds) exhibit reproducible activity spanning over three orders of magnitude (and applied together potentially over six orders of magnitude). Many of these parts outperform the widely used standard parts, and may be useful simply for maximizing genome editing rates in routine experiments.

More sophisticated applications may include any genome engineering or synthetic biology project in which simplified assembly, miniaturization and/or activity titration would be beneficial. Although we focused on simplified assembly for molecular recording in the follow-up experiments reported here, other applications which will benefit from both simplified assembly and miniaturized parts include packaging multiple U6p-gRNA cassettes into recombination-prone viral vectors commonly used in CRISPR screens^11,34,59–62^ or for gene therapy, while applications which will benefit from activity titration include the design and implementation of complex genetic circuits.

Although genome editing activity can also be titrated via spacer mismatches as demonstrated by Jost et al.^42^, titrating activity via the Pol III promoter or gRNA scaffold may have the advantage of being more generic across targets. In particular, diversified Pol III promoters offer a more general solution, as they are not directly impacted by changes in spacer/PBS/RTT sequences like spacer mismatches or diversified scaffolds, and achieve titration at the level of transcription. In support of this viewpoint, relative Pol III promoter activity levels were more consistent across alternate targets. Pol III promoter parts may also be useful for non-CRISPR synthetic biology applications relying on quantitative control of short RNA expression.

We based our multiplex functional assay on prime editing because this permitted the use of part-specific insertional barcodes, facilitating straightforward quantitation of the relative activity of thousands of parts in a single experiment. Quantifying genome editing in addition to RNA abundance was critical, as diversified Pol III promoters can have variable amounts of Pol II activity, which can produce alternative transcripts that are abundant yet fail to drive genome editing^39,48,63,64^. We initially elected to target endogenous *HEK3* because of its well-documented efficiency for insertional prime editing^38,31,39^. However, we found the resulting activity measurements to generalize across human and mouse cellular contexts, as well as across endogenous genomic loci. Indeed, while both diversified Pol III promoters and gRNA scaffolds exhibited reasonably consistent activities across five endogenous target loci, Pol III promoters exhibited stronger reproducibility in this regard, presumably because in contrast with scaffold sequences, they titrate gRNA levels at the earlier step of transcription and have no opportunity to directly interact with the target sequence^14,46,65^.

For similar reasons, we predict that diversified promoters and scaffolds will also be combinable with other variations on Cas9-mediated genome editing both at the protein (*e.g.* nuclease editing, CRISPR i/a editing, etc.) and guide (*e.g.* epegRNAs with structured motifs at their 3’ end^46^) levels. Indeed, the A-U flip design has recently been used successfully with epegRNAs for improved performance^46^, and we expect the same will be possible with other high-performance scaffold alternatives identified here. Further, the advent of PE7, which fuses an endogenous human RNA-binding domain to PEmax, offers performance on par with epegRNAs while enabling use of shorter, less repetitive standard pegRNAs such as the ones diversified here (probably by conferring pegRNA stability through protein-binding rather than secondary structure)^66^. Similarly, the parts described here may be synergistic with Cas12a arrays and related approaches to multiplex gRNAs in a single transcript, *e.g.* by enabling ‘nested multiplexing’ through assembly and delivery of multiple independent gRNA arrays on a single construct with multiple diversified and/or miniaturized U6 promoters^67–69^.

In our view, among the most exciting use-cases for this parts list lie in the field of molecular recording^4,8,70^. Following up on our goals in setting out in this direction, we demonstrated that these sequence-diversified parts are amenable to single step assembly in yeast and deployment in mammalian cells as a single-locus, ten-key DNA Typewriter. Further, these experiments revealed that the activity of Pol III promoter-pegRNA-iBC combinations (and arrays thereof) can be predicted based on individual part activity measurements, something that was unclear at the outset of this work. Using these parts and following the strategy we have demonstrated here, one could imagine assembling, in yeast and as a single locus, many more iterations and combinations of multi-unit arrayed CRISPR clocks^71^, transcriptional^39^ or lineage recorders^19,22,31,72,73^ that write to their DNA recording medium at different rates in parallel, to concurrently access different temporal resolutions and time scales.

We envision that the strategy taken here, namely combining evolutionary mining with rational design and multiplex functional assays, will advance the realization of a long-standing goal of synthetic biology -- the delineation of sequence-diversified, functionally-diversified, cross-compatible “parts”, that can be routinely and cost-effectively assembled to build complex genetic circuits that will behave in a predictable manner^9,10^. A further vision is that the quantitative characterization of these parts will essentially serve as “pre-training” for generative models that can *de novo* design circuits that function as predicted and allow us to access a vast range of possibilities within the space of intracellular circuits.

## Methods

### Library design and cloning

#### U6 promoter libraries

Promoter sequences of vertebrate orthologs of known transcriptionally active human U6 small nuclear RNA genes were obtained from the ENSEMBL database^74,75^. Sequences were selected for diversity initially using the distance metric from hierarchical clustering (Clustal Omega multiple sequence alignment)^76^ followed by *L*_*max*_ calculations (detailed below) to ensure all promoters satisfied *L*_*max*_ < 40. Additional, sufficiently diverse U6 promoters from vertebrate species (n=4)^11^, transcriptionally active human U6 promoters (n=3)^33^, as well as the canonical human RNU6–1 promoter were also included. Synthetically diversified hRNU6–1 promoters were generated by shuffling nucleotides between the core TFBSs (OCT, SPH, PSE and TATA box) using custom R scripts^77–81^. In a subset of cases, we consulted TFBS profiles from the JASPAR database^82,83^ and further introduced putatively tolerated variants into TFBSs and/or random 3bp spacers sequences between sites (spacers are present in other transcriptional active human U6 promoters) to further increase diversity (n = 52 variants via non-TFBS sequence permutation, n = 30 variants via non-TFBS sequence permutation and SPH TFBS mutation, n = 30 variants via non-TFBS sequence permutation and introduction of random 3bp spacer sequence between the OCT and SPH TFBSs). A modestly larger set of diversified parts were first isolated / designed using both evolutionary and synthetic approaches, then a subset that were amenable to commercial synthesis and which also satisfied *L*_*max*_ < 40 as a combined library were ordered and assessed. This workflow resulted in the combined library of 209 diversified U6 promoters. U6 promoter-pegRNA-pBC cassettes were ordered as eBlocks (IDT) with flanking BsaI golden gate assembly sites^84^. BsaI restriction sites were removed from promoter sequences where required to enable cloning. Also where required, 5bp buffer sequences were inserted flanking the 5’ restriction site to enable commercial synthesis. U6p-pegRNA-pBC eBlocks were then pooled, BsaI digested and ligated (NEB, Cat. No. R3733L) at a 2:1 insert:vector ratio into a minimal backbone^39^ (Twist Bioscience). Cloned libraries were then electroporated into NEB 10-beta electrocompetent E. coli (NEB, Cat. No. C3020K), cultured at 30°C overnight and prepared using a Zymo Pure II (Cat. No. D4200) kit following manufacturer protocols. Singleton validation constructs were confirmed via whole-plasmid sequencing (Primordium Labs). Primer sequences are provided in Table S12. U6p-pegRNA-pBC sequences are provided in Table S1.

#### pegRNA scaffold libraries

Diversified pegRNA sequences containing complementary R:AR replacement and extension variants (Fig. 2a) were generated and paired with respective 5N pBCs using custom R scripts^77–81^. Diversified pegRNA-pBC cassettes were ordered as oligo pools (IDT) with flanking BsaI sites. Oligos were double stranded across multiple low cycle PCR reactions using Q5 polymerase (NEB, Cat. No. M0492L; cycling conditions: 98°C for 30 seconds, 5 cycles of 98°C × 10 seconds, 65°C × 15 seconds and 72°C × 30 seconds). PCR products were then pooled and purified using 2.0x AMPure XP beads (Beckman Coulter, Cat. No. A63880), then BsaI digested and ligated (NEB, Cat. No. R3733L) into a backbone with the standard hRNU6–1 promoter for expression. Plasmid library DNA was prepared as above. Primer sequences are provided in Table S12. Diversified pegRNA-pBC sequences are provided in Table S4.

#### Miniaturized hRNU6–1p saturation mutagenesis libraries

Saturation mutagenesis variant sequences of the miniaturized hRNU6–1p cassette (Fig. 3) were generated and paired with respective 5N pBCs using custom R scripts^77–81^. For the initial deletion series experiments (Fig. 3a,b), miniaturized U6p-pegRNA cassettes were ordered as eBlocks with four independent iBCs and cloned as described above for the diversified U6 promoter libraries. Saturation mutagenesis pBC cassettes were ordered as oligo pools (IDT) with flanking BsaI sites. Oligos were double stranded across multiple low cycle PCRs using Q5 polymerase (NEB, Cat. No. M0492L; cycling conditions: 98°C for 30 seconds, 5 cycles of 98°C × 10 seconds, 65°C × 15 seconds and 72°C × 30 seconds). PCR products were then pooled and purified using 2.0x AMPure XP beads (Beckman Coulter, Cat. No. A63880), then BsaI digested and ligated (NEB, Cat. No. R3733L) into a minimal backbone. Plasmid library DNA was prepared as above. Primer sequences are provided in Table S12. Diversified min. hRNU6–1p-pegRNA-BC sequences are provided in Table S5.

### Orthologous Pol III promoters (Zoonomia), H1 and 7SK saturation mutagenesis libraries

To select orthologous sequences, we leveraged the cactus alignment (2020v2) from the Zoonomia consortium^54^, relying on the Hal suite of tools^85^.

Briefly, HalLiftover (cactus-bin-v2.7.1) was used with the human interval (hg38) of the Pol 3 promoter as query sequence to all 241 extant mammalian genomes and their reconstructed ancestral sequences (options: --bedType 4 --noDupes 241-mammalian-2020v2.hal). The resulting possibly discontiguous output orthologous intervals were then merged with stitchHalFrags_v2^86^ (modified), requiring that the final interval was within 0.5 to 1.5 fold in length compared to the original query interval size. Sequences not meeting this size threshold were discarded from downstream analysis. In cases for which the intervals spanned different contigs, the sequences were also discarded to avoid complications.

The nucleotide sequences were then obtained from the merged bed file using bedtools (version 2.29.2) getfasta with options -s -fi using the hal genome of the corresponding target species. All resulting sequences with one or more undetermined bases (N) within the queried orthologous region were discarded. Both orientations of remaining orthologous sequences were then pairwise aligned (Biostrings 2.62.0 , pairwiseAlignment, options: type=“global”, gapOpening = −2, gapExtension = −8) to their human counterpart to determine correct orientation. The final orientation considered was the one with the largest alignment score to the starting human sequence. After the various filters, out of 481 possible extant and ancestral reconstructed genomes, 437 H1, 426 7SK, 454 U6, 358 RNU6_2, 285 RNU6_7, 286 RN6_8, and 340 RNU6_9 promoter sequences were obtained. Resulting promoters were paired with respective 8N pBCs using custom R scripts^77–81^.

Saturation mutagenesis variant sequences of the human H1 and 7SK promoters were generated and paired with respective 8N pBCs using custom R scripts^77–81^.

Pol III promoter - scaffold - iBC cassettes were ordered as 500bp oligos from Twist with flanking dial out PCR primers^87^ for double stranding and isolation as well as BsaI restriction sites for cloning. BsaI restriction sites were removed from promoter sequences where required to enable cloning. Buffer sequence was added 5’ to the first BsaI site for shorter promoter sequences to assure equivalent size during low-cycle double stranding and subpool isolation PCR (prior to removal following BsaI digestion/cloning). Oligos were double stranded across multiple low cycle PCRs using Q5 polymerase (NEB, Cat. No. M0492L; cycling conditions: 98°C for 30 seconds, 5 cycles of 98°C × 10 seconds, 65°C × 15 seconds and 72°C × 30 seconds). PCR products were then pooled and purified using 2.0x AMPure XP beads (Beckman Coulter, Cat. No. A63880), then BsaI digested and ligated (NEB, Cat. No. R3733L) into a minimal backbone. Plasmid library DNA was prepared as above. Primer sequences are provided in Table S12. Pol III promoter and pBC sequences are provided in Table S7.

#### Alternative target loci validation libraries

20 Pol III promoters (19 diversified and the standard hRNU6–1p) and all 313 scaffolds (312 diversified and the standard) were selected for alternative target loci validations. 100 additional scaffold variants that preserve the transcription-enhancing A-U flip variant while introducing additional diversifying R:AR replacement variants were also included (variants introduced as described above). Each of the two sets of parts (20 Pol III promoters and 413 scaffolds) were targeted to 5 different target loci (*synHEK3*, *HBB*, *EMX1*, *CLYBL*, and *FANCF*) by pairing them with the corresponding spacers, PBSs, and RTTs using custom R scripts. Spacer, RTT, and PBS sequences were either selected from the literature^45^ or designed with PRIDICT2.0^88^. Each of these designs were then assigned 3 unique 8N iBCs for a total of 300 promoter designs (20 promoters *x* 3 8N iBCs *x* 5 target loci = 300 constructs) and 6,195 gRNA scaffold designs (413 scaffolds *x* 3 8N iBCs *x* 5 target loci). The promoter library was ordered as double-stranded DNA fragments (Twist Bioscience) with flanking BsaI golden gate assembly sites^84^. BsaI restriction sites were removed from promoter sequences where required to enable cloning. U6p-pegRNA-pBC fragments were then pooled, BsaI digested and ligated (NEB, Cat. No. R3733L) at a 2:1 insert:vector ratio into a minimal backbone^39^ (Twist Bioscience). Plasmid library DNA was prepared as above. Primer sequences are provided in Table S12. U6p sequences are provided in Table S9. Diversified pegRNA-pBC cassettes were ordered as an oligo pool (Twist Bioscience) with flanking BsaI sites. Oligos were double stranded across multiple low cycle PCR reactions using Q5 polymerase (NEB, Cat. No. M0492L; cycling conditions: 98°C for 30 seconds, 5 cycles of 98°C × 10 seconds, 65°C × 15 seconds and 72°C × 30 seconds). PCR products were then pooled and purified using 2.0x AMPure XP beads (Beckman Coulter, Cat. No. A63880), then BsaI digested and ligated (NEB, Cat. No. R3733L) into a backbone with the standard hRNU6–1 promoter for expression. Plasmid library DNA was prepared as above. Primer sequences are provided in Table S12. Diversified scaffold sequences are provided in Table S10.

#### 10-unit diversified molecular recording array design and assembly

Ten of the top diversified U6 promoter and pegRNA scaffold sequences were paired to balance activity levels based on primary part activity measurements . Top parts were selected based on the highest median edit score across cellular contexts. For diversified scaffolds the edit scores from the second barcode pool were used. These Top 10 promoter-scaffold pairings were further assigned specific “GGANNN” DNA Typewriter iBCs^31^ using custom R scripts (Fig. 5). The resulting diversified U6p-pegRNA-iBC units were paired with flanking VEGAS adapters^56^ (10 units, 11 VEGAS adapters) and ordered as sequence-verified double-stranded fragments from STOMICS (Supplementary Fig. 12). Additional segments containing auxiliary sequences (ARE, ITRs, etc.) and left/right backbone linkers were also ordered as sequence-verified double-stranded fragments from STOMICS (Supplementary Fig. 12). Upon arrival, fragments were amplified using Q5 polymerase, size-verified on a 1% agarose gel, and purified using a Zymo clean and concentrate kit (Cat. No. D4013). Primer sequences are provided in Table S12. To generate the backbone fragment, 1 μg of the vector backbone (pSP0769) was linearized using PmeI (NEB, Cat. No. R0560S) for 1h and gel purified.

All resulting fragments were transformed into yeast (*Saccharomyces cerevisiae*) for single-step assembly using the following protocol: 1. The yeast strain BY4741 was grown overnight in 5 mL of 2% YPD media (1% yeast extract, 2% peptone, and 2% dextrose). 2. 1 mL of the overnight yeast culture was transferred to 20 mL of 2% YPD and cultivated for 4 hours at 30°C and 200 rpm. 3. The cells were harvested at 300g for 3 minutes and washed with 20 mL of water. 4. The cells were harvested again and washed with 0.1M Lithium Acetate (LiAc). 5. The cells were harvested, and the supernatant was removed. The cell pellet was then resuspended in 0.1M LiAc that remained in the tube. 6. The cells were transferred to a 1.5 mL tube, harvested at 300g for 3 minutes, resuspended in 200 μL of 0.1M LiAc, and kept on ice. 7. The segments and linearized vector were pooled together at a concentration of approximately 0.5 pmol each. 8. The transformation mix was prepared by combining 240 μL of 44% polyethylene glycol (PEG) solution, 36 μL of 1M LiAc, and 25 μL of herring sperm DNA. 9. 20 μL of cells were transferred to the tube containing the pooled DNA and vortexed briefly. 10. The transformation mix was added to the DNA + cells solution, and the mixture was vortexed at high speed for 10 seconds. 11. The mixture was transferred to a 30°C incubator with rotation and left for 30 minutes. 12. 36 μL of DMSO was added to the tube, followed by a 15-minute incubation at 42°C using a water bath. 13. Cells were harvested and resuspended in 200 μL of 5 mM CaCl₂ before being plated onto SC -LEU plates. 14. Plates were incubated at 30°C, and the presence of colonies was checked after 2–3 days.

Candidates were initially checked through junction PCR, where the presence of each junction between all the transformed segments was verified using segment-specific primer pairs (Supplementary Fig. 12). Primer sequences are provided in Table S12. Yeast cells that passed this initial check were grown in 5 mL of SC -LEU media overnight, and the plasmids were extracted using the yeast miniprep I kit from Zymo Research (Cat. No. D2001). The plasmids were then transformed into E. coli (EPI300 cells) through electroporation. E. coli cells were subjected to a miniprep (Zymo kit), and the 10-unit assembly construct was sequence-verified through commercial long-read sequencing (Plasmidsaurus-nanopore). The final construct presented only a single SNP at the first base of U6 promoter number 5 (Supplementary Fig. 12).

For the repetitive vs. diversified part assemblies, we PCR amplified either the 10 existing diversified U6p-pegRNA-iBC units or 10 newly synthesized repetitive U6p-pegRNA-iBC units (repeats of the standard hRNU6–1p and gRNA scaffolds with 10 unique iBCs) with requisite VEGAS adapters. We then transformed these pieces into yeast in two separate reactions with the other auxiliary and backbone fragments for assembly as described above. Resulting colonies were then scraped off culture plates, washed twice and grown out in liquid culture for ~6 hours before pelleting and gDNA extraction. We then submitted the gDNA for commercial long-read sequencing (Plasmidsaurus-nanopore). For analysis, we created a custom reference sequence consisting of the VEGAS adapters, with each adapter as a separate contig. We then aligned reads from both samples to this reference using Minimap2^89^, parsed reads to identify those mapping to adapters as well as the position of adapters within those mapped reads using custom scripts. We then quantified how many of the 9 assembly junctions were present in each read in each of the two conditions.

#### *L*_*max*_ calculations

To calculate Lmax, we wrote a pipeline that takes as input a list of sequences and first generates a dataframe containing all possible pairs of sequences in the forward and reverse orientation: n possible pairs = (n × n−1)/2 in each orientation. The pipeline iterates through each row of the sequence-pair dataframe applying a longest common substring function^90^ to return the length and identity of the longest shared sequence repeat in each pair of sequences in a given set (Supplementary Fig. 1). The resulting *L*_*max*_ distributions can be filtered to select sets of sequences that satisfy any *L*_*max*_ threshold (e.g. *L*_*max*_ < 40 used here) (Supplementary Fig. 1).

### Cell Lines and Culture

#### K562 cell culture

K562 cells (ATCC Cat. No. CCL-243)^91^ were grown with 5% CO_2_ at 37°C and cultured in RPMI 1640 + L-Glutamine (GIBCO, Cat. No. 11-875-093) supplemented with 10% fetal bovine serum (Rocky Mountain Biologicals, Cat No. FBS-BSC) and 1% penicillin-streptomycin (Thermo Fisher Scientific, Cat. No. 15070063).

#### HEK293T cell culture

HEK293T cells (ATCC Cat. No. CRL-11268) were grown with 5% CO_2_ at 37°C and cultured in high glucose DMEM (GIBCO, Cat. No. 11965092) supplemented with 10% fetal bovine serum (Rocky Mountain Biologicals, Cat No. FBS-BSC) and 1% penicillin-streptomycin (Thermo Fisher Scientific, Cat. No. 15070063).

#### iPSC culture

WTC11 iPSCs^92^ were grown with 5% CO_2_ at 37°C cultured in mTeSR Plus Basal Medium (Stemcell technologies; Cat. No. 100–0276) on Greiner Cellstar plates (Sigma-Aldrich; assorted Cat. Nos.) coated with Geltrex^™^ LDEV-Free, hESC-Qualified, Reduced Growth Factor Basement Membrane Matrix (Gibco; Cat. No. A1413302) diluted 1:100 in Knockout DMEM (GIBCO/Thermo Fisher Scientific; Cat. No. 10829018). Cells were passaged by washing cells with PBS (GIBCO/Thermo Fisher Scientific; Cat. No. 10010023), dissociating with StemPro Accutase Cell Dissociation Reagent (GIBCO/Thermo Fisher Scientific; Cat. No. A1110501) and resuspending cell pellets in mTeSR Plus Basal Medium supplemented with 0.1% dihydrochloride ROCK Inhibitor (Stemcell technologies; Cat. No. Y-27632). mTeSR plus media was replaced every other day.

#### mESC culture

E14 mESCs were grown with 5% CO2 at 37°C cultured in media composed of Advanced DMEM (Gibco, cat. no. 11965118) supplemented with 15% KSR (Gibco, cat. no. 10828028), 1X NEAA (Gibco, cat. no. 11140050), 1X Glutamax (Gibco, cat. no. 35050061), 1 mM sodium pyruvate (Gibco), 0.5 μM 2-Mercaptoethanol (ThermoFisher, cat. no. 31350010), and 1000 U/ml LIF (ESGRO) on 6cm dishes that had been pre-coated with 0.2% gelatin (Millipore Sigma, cat. no. G1890). For passaging, cells were dissociated with 0.05% Trypsin-EDTA (Gibco, cat. no. 25300120), pipetted gently to generate a single-cell suspension, then the trypsinization reaction was quenched with a wash medium composed of Advanced DMEM/F-12 (Gibco, cat. no. 12634010) supplemented with 5% FBS (Cytiva, cat. no. SH30071.03HI) before resuspending in culture media. Culture media was replaced every day.

### Cell line generation

#### K562

The monoclonal PE2-K562 cell line was generated using piggyBac transposition. Specifically, 500ng of a PE2 cargo construct^93^ and 100ng of a super piggyBac transposase expression vector (System Biosciences, Cat. No. PB210PA-1) were mixed and transfected using lipofectamine 3000 (Thermo Fisher Scientific; Cat. No. L3000015) following manufacturer protocol. PE2 expressing cells were then selected by antibiotic resistance (puromycin), single cell-sorted into 96-well plates using a flow sorter and cultured for 2–3 weeks until confluency. Multiple lines were then tested for prime editing insertion efficiency using a pegRNA expression construct programmed to insert “CTT” at the *HEK3* locus (Addgene #132778)^38^ and the line with the highest editing efficiency was selected for use. The K562 line with 22 *synHEK3* sites was generated using piggyBac transposition and mapped with a T7-promoter strategy as previously described^49^.

#### HEK293T

The polyclonal PEmax-HEK293T cell line was generated using piggyBac transposition. Specifically, a PEmax cargo construct and super piggyBac transposase expression vector (System Biosciences, Cat. No. PB210PA-1) were mixed and transfected using lipofectamine 3000 (Thermo Fisher Scientific; Cat. No. L3000015) at a 5:1 molar ratio following manufacturer protocol. PEmax expressing cells were then selected by antibiotic resistance (blasticidin) and PEmax expression was confirmed by fluorescence. The polyclonal 6xTAPE-PEmax-HEK293T line was generated using piggyBac transposition into the previously generated HEK293T-PEmax line. Specifically, 2,160ng of 6xTAPE construct, and 240 ng of a super piggyBac transposase expression vector (System Biosciences, Cat. No. PB210PA-1) were mixed and transfected using lipofectamine 3000 (Thermo Fisher Scientific; Cat. No. L3000015) following manufacturer protocol and then selected with 400 ug/mL of hygromycin for 1 week.

The monoclonal PEmax-*synHEK3*-HEK293T line was generated by two steps of piggyBac transposition. First, a PEmax cargo construct and a super piggyBac transposase expression vector (System Biosciences, Cat. No. PB210PA-1) were mixed at a 1:1 molar ratio and transfected using lipofectamine 3000 (Thermo Fisher Scientific; Cat. No. L3000015) following manufacturer protocol. PEmax-expressing cells were then selected by antibiotic resistance (blasticidin) for 14 days. From this monoclonal line, a second piggyBac transposition was performed with a *synHEK3* cargo construct, a super piggyBac transposase expression vector, and a GFP expression vector. These were mixed at an approximate 5:1 molar ratio of cargo to piggybac with a small fraction of the GFP expression vector (83%, 12%, 5%) and transfected using lipofectamine 3000 (Thermo Fisher Scientific; Cat. No. L3000015) following manufacturer protocol. The cells were passaged for 10 days to allow the plasmid to dilute out, then the top 5% of cells by GFP expression were sorted into a 96 well plate at a single cell per well. Monoclonal colonies were grown in these wells and then frozen for future use. The number of integrated *synHEK3* target sites was estimated using diverse barcodes paired with each *synHEK3* target construct that were prepared and sequenced as part of the target amplicon library (see below). Following expansion and sequencing, the HEK293T line with the highest estimated number of *synHEK3* target sites was used for library screening.

#### iPSC

The monoclonal PEmax WTC11 iPSC line was generated by piggyBac transposition. Specifically, a PEmax cargo construct and a super piggyBac transposase expression vector (System Biosciences, Cat. No. PB210PA-1) were mixed at a 5:1 molar ratio and nucleofected using the CB-150 program and P3 primary reagents (Lonza, Cat. No. V4XP-3032) on a Lonza 4D nucleofector following manufacturer protocol. PEmax expressing cells were then selected by antibiotic resistance (blasticidin), single cell-sorted using limiting dilution, and cultured for 2–3 weeks until confluent. Multiple lines were tested for 5N prime editing insertion efficiency at the *HEK3* locus and the line with the highest editing efficiency was selected for use.

#### mESC

The monoclonal PEmax-*synHEK3* E14 mESC line was generated by piggyBac transposition. Specifically, a PEmax cargo construct, a *synHEK3* cargo construct, and a super piggyBac transposase expression vector (System Biosciences, Cat. No. PB210PA-1) were mixed at a 17:2:1 molar ratio (85%, 10%, 5%) and transfected using lipofectamine 2000 (Thermo Fisher Scientific; Cat. No. 11668027) following manufacturer protocol. PEmax-expressing cells were then selected by antibiotic resistance (puromycin) for seven days, then the top 10% of GFP+ cells were sorted into a single-cell suspension. These sorted cells were plated on a feeder layer of mitotically inactive mouse embryo fibroblasts (MEFs) to grow into colonies. Monoclonal colonies were then handpicked and further expanded and frozen for future use. The number of integrated *synHEK3* target sites was estimated using diverse barcodes paired with each *synHEK3* target construct that were prepared sequenced as part of the target amplicon library (see below). All clones sorted in this manner had high copy numbers of *synHEK3* target sites. We then chose one of these clones at random and used it for library screening.

### Transfection

#### K562

All libraries were transfected using lipofectamine 3000 (Thermo Fisher Scientific; Cat. No. L3000015) following manufacturer's specifications. 1.5 × 10^5^ cells were seeded the day prior to transfection. 500 ng of each library was then mixed with 100 ng of a GFP co-transformation marker (pmaxGFP, Lonza) and transfected in triplicate or quadruplicate in 24 well plates. Genomic DNA was harvested from cells 3–4 days after transfection. For the 8N iBC and Zoonomia Pol III promoter libraries, 1 × 10^6^ cells were nucleofected with 1000 ng library, 1000 ng of a PEmax construct, and 250 ng of a GFP co-transformation marker (pmaxGFP, Lonza) using a Lonza 4D nucleofector (Lonza; Cat. No. V4SC-2096) in quadruplicate following manufacturer's specifications. For the combined transcription score and edit score experiments for the 1024 5N iBC library, 209 diversified U6 promoter library, and 3,566 diversified Pol III promoter library 1 × 10^6^ cells were nucleofected with 1000 ng library, 1000 ng of a PEmax construct, and 250 ng of a GFP co-transformation marker (pmaxGFP, Lonza) using a Lonza 4D nucleofector (Lonza; Cat. No. V4SC-2096) in quadruplicate following manufacturer's specifications. 4 days after transfection, cell were split and genomic DNA was harvested using freshly prepared lysis buffer (described below) and total RNA was harvested using a Zymo Direct-zol kit (Cat. No. R2050).

#### HEK293T

All libraries were transfected using lipofectamine 3000 (Thermo Fisher Scientific; Cat. No. L3000015) following manufacturer's specifications. 3 × 10^5^ cells were seeded the day prior to transfection. For the primary 209 diversified U6 promoter library, 1000 ng of each library was then mixed with 250 ng of a GFP co-transformation marker (pmaxGFP, Lonza) and transfected across 8 wells of a 12 well plate. For the 1024 5N iBC library, 125 ng of the library was mixed with 500 ng of a PE2 construct and transfected in triplicate in a 24 well plate. For the primary diversified pegRNA and saturation mutagenesis libraries, 500 ng of each library was then mixed with 100 ng of a GFP co-transformation marker (pmaxGFP, Lonza) and transfected in triplicate or quadruplicate in 24 well plates. For the 209 diversified U6 promoter library, 20 diversified Pol III promoter alternative target loci library, and 413 diversified scaffold alternative target loci library tested in the monoclonal HEK293T-*synHEK3*-PEmax line, 625 ng of each library was mixed with 125 ng of a PEmax-mCherry construct and transfected in quadruplicate in 24 well plates. Genomic DNA was harvested from cells 3–4 days after transfection. The 10-unit assembly was delivered via transfection with lipofectamine 3000 (Thermo Fisher Scientific; Cat. No. L3000015) following manufacturer's specifications. 3 × 10^5^ cells were seeded the day prior to transfection. 500 ng of the 10-unit assembly was then mixed with 300 ng of a PEmax construct, 125 ng of a GFP co-transformation marker (pmaxGFP, Lonza), and 100 ng of a super piggyBac transposase expression vector (System Biosciences, Cat. No. PB210PA-1) and transfected across 4 wells of a 24 well plate.

#### iPSCs

All libraries were nucleofected using a Lonza 4D nucleofector following manufacturer's specifications. iPSCs were dissociated and resuspended in mTeSR plus basal media supplemented with ROCKi. 2.2 × 10^5^ cells were nucleofected with 2000 ng of each library, 1000 ng of PEmax construct, and 500 ng of pmaxGFP co-transformation marker (Lonza) using P3 reagents and the CB-150 program on the Lonza 4D nucleofector. Four replicate nucleofections per library were then plated into separate Geltrex coated wells of a 24 well plate. Genomic DNA was harvested from cells 3–4 days after nucleofection.

#### mESCs

All libraries were transfected using lipofectamine 2000 (Thermo Fisher Scientific; Cat. No. 11668019) following manufacturer's specifications. Transfection reagents were mixed with DNA (1300 ng of library and 145 ng of PEmax per replicate) and allowed to incubate for 20 minutes. During this time, 3.6 × 10^5^ freshly dissociated cells were plated into each of four gelatin-coated wells of a 12 well plate. Transfection reagents were then added to the cells while still in suspension. The plate was then placed in the incubator at 37°C at 5% CO2 and gently rocked across its horizontal and vertical axis to evenly plate the cells. Media was changed the day after transfection. Genomic DNA was harvested from cells 3 days after transfection.

### Genomic DNA extraction

Genomic DNA was extracted as follows: Harvested cells were washed with PBS, then 200 μl of freshly prepared lysis buffer (10 mM Tris-HCl, pH 7.5; 0.05% SDS; 25 μg/ml protease (Thermo Fisher Scientific, Cat. No. EO0491)) per 0.5–1M cells was added directly into each well of the tissue culture plate. The genomic DNA mixture was then incubated at 50°C for 1 h, followed by a 30 min 80°C enzyme inactivation step.

### Library preparation and sequencing

#### Plasmid barcode amplicon sequencing library preparation

pBC amplicon sequencing libraries were generated using a two step PCR process to amplify barcodes then append sequencing adapters and sample indices. pBCs were amplified using a forward primer that binds the gRNA scaffold (U6 promoters), the hRNU6–1 promoter (pegRNA libraries), or upstream of the miniaturized U6 promoter (saturation mutagenesis miniaturized hRNU6–1p-pegRNA libraries) along with a universal reverse primer that binds the plasmid backbone. Plasmid libraries were amplified using Q5 polymerase in quadruplicate (NEB, Cat. No. M0492L; cycling conditions: 98°C for 30 seconds, 15 cycles of 98°C × 10 seconds, 65°C × 15 seconds and 72°C × 40 seconds). SYBR Green (Thermo Fisher Scientific, Cat. No. S7567) was added to track the amplification curve. PCR products were pooled and purified using 1.2x AMPure XP beads (Beckman Coulter, Cat. No. A63880). Sequence flow cell adapters and dual sample indices were then appended in the second PCR reaction using Q5 polymerase (NEB, Cat. No. M0492L; cycling conditions: 98°C for 30 seconds, 5 cycles of 98°C × 10 seconds, 65°C × 15 seconds and 72°C × 30 seconds). PCR products were purified using 0.9x AMPure XP beads (Beckman Coulter, Cat. No. A63880) and assessed on an Agilent 4200 TapeStation before sequencing. Primer sequences are provided in Table S12.

#### *HEK3* locus, *synHEK3*, and alternative target loci amplicon sequencing library preparation

The *HEK3*, *synHEK3, HBB, EMX1, FANCF, and CLYBL* target loci amplicon sequencing libraries were generated using a similar two step PCR process to amplify targets then append sequencing adapters and sample indices. 2ul of cell lysate was used as input to a 50ul PCR reaction using KAPA Robust polymerase (KAPA Biosystems, Cat. No. 2GRHSRMKB; cycling conditions: 95°C for 3 min, 22–29 cycles of 95°C × 15 seconds, 65°C × 15 seconds and 72°C × 30 seconds). SYBR Green (Thermo Fisher Scientific, Cat. No. S7567) was added to track the amplification curve. PCR products were pooled and purified using 1.2x AMPure XP beads (Beckman Coulter, Cat. No. A63880). Sequence flow cell adapters and dual sample indices were then appended in a second PCR reaction (cycling conditions: 98°C for 30 seconds, 5 cycles of 98°C × 10 seconds, 65°C × 15 seconds and 72°C × 30 seconds). PCR products were purified using 0.9x AMPure XP beads (Beckman Coulter, Cat. No. A63880) and assessed on an Agilent 4200 TapeStation before sequencing. Primer sequences are provided in Table S12.

#### pegRNA reverse transcription and amplicon sequencing library preparation

cDNA was generated using SuperScript IV reverse transcriptase with a primer targeted to the 3’ end of the pegRNA following manufacturer's specifications for gene-specific primers. The pegRNA cDNA amplicon sequencing libraries were generated using a similar two step PCR process to amplify targets then append sequencing adapters and sample indices. 4ul of cDNA was used as input to a 50ul PCR reaction using KAPA Robust polymerase (KAPA Biosystems, Cat. No. 2GRHSRMKB; cycling conditions: 95°C for 3 min, 22–29 cycles of 95°C × 15 seconds, 65°C × 15 seconds and 72°C × 30 seconds). SYBR Green (Thermo Fisher Scientific, Cat. No. S7567) was added to track the amplification curve. PCR products were pooled and purified using 1.2x AMPure XP beads (Beckman Coulter, Cat. No. A63880). Sequence flow cell adapters and dual sample indices were then appended in a second PCR reaction (cycling conditions: 98°C for 30 seconds, 5 cycles of 98°C × 10 seconds, 65°C × 15 seconds and 72°C × 30 seconds). PCR products were purified using 1.0x AMPure XP beads (Beckman Coulter, Cat. No. A63880) and assessed on an Agilent 4200 TapeStation before sequencing. Primer sequences are provided in Table S12.

Libraries were sequenced on an Illumina MiSeq sequencer, lllumina NextSeq500 sequencer, or Illumina NextSeq2000 sequencer following manufacturer's protocol. FASTQ files were demultiplexed with bcl2fastq (v.2.20, Illumina).

#### Edit score calculations and insertion barcode normalization

pBC and iBC counts were extracted from plasmid library and *HEK3* locus sequencing reads using pattern matching functions. Specifically, we required a perfect match to the 15bp spanning the intended 5N barcode and sequences flanking the edit site in the RTT and PBS in plasmid and edited read datasets to count a barcode. For 8N barcodes this was extended to 18bp. pBC and iBC frequencies were then calculated for each library, and the raw edit score was calculated as iBC freq. / pBC freq. for each replicate. Raw edit scores were divided by the normalized insertion efficiency of the paired barcode to correct for insertion barcode efficiency (Supplementary Figs. 2 and 13). Correlations between cellular contexts were calculated on the barcode-normalized edit scores. Note we initially selected and tested an additional four promoters and two scaffolds with hierarchical clustering as the diversity metric that ultimately did not satisfy the more stringent criterion of *L*_*max*_ < 40 and so were removed from analysis and final reported functional part sets. Any part with an edit score below 0.005 was assigned an edit score of zero in final results tables (Tables S1–S3).

## Supplementary Material

FigS1-26_SI_Promoter_And_Scaffold_Parts_McDiarmid

TablesS1-S12_Promoter_And_Scaffold_Parts_McDiarmidpdf

## Data Availability

Raw sequencing data have been uploaded on Sequencing Read Archive (SRA) with associated BioProject ID PRJNA1161643 (https://www.ncbi.nlm.nih.gov/bioproject/PRJNA1161643)^94^.

## Code availability

Analysis and visualization code are available at GitHub (https://github.com/shendurelab/Diversified_Parts/), together with construct maps and custom sequencing amplicons used in this work^95^.
