# Supplementary material for: A diversified parts list for mammalian genome engineering and molecular recording
Source: Nat Biotechnol. Author manuscript; Available in PMC 2026 Jul 11. (PMC13355316; doi:10.1038/s41587-025-02896-2)
Supplement: FigS1-26_SI_Promoter_And_Scaffold_Parts_McDiarmid [file NIHMS2185944-supplement-FigS1-26_SI_Promoter_And_Scaffold_Parts_McDiarmid.pdf]

Supplementary Figures

a

1. Input sequence list

ID	Sequence
1	TCTTGGCCTGCTTGGTGTCT
2	CTCACCTGTGGGAGTAACGG
3	ACCGCTGCTGGGAGTAACGG
4	CCACATACGAAATGCCAACG

2. Generate all possible pairs

ID	ID	Seq_A	Seq_B
1	2	TCTTGGCCTGCTTGGTGTCT	CTCACCTGTGGGAGTAACGG
1	3	TCTTGGCCTGCTTGGTGTCT	ACCGCTGCTGGGAGTAACGG
1	4	TCTTGGCCTGCTTGGTGTCT	CCACATACGAAATGCCAACG
2	3	CTCACCTGTGGGAGTAACGG	ACCGCTGCTGGGAGTAACGG
2	4	CTCACCTGTGGGAGTAACGG	CCACATACGAAATGCCAACG
3	4	ACCGCTGCTGGGAGTAACGG	CCACATACGAAATGCCAACG

3. Identify longest shared repeat (both strands) and quantify L_{max}

ID	ID	Seq_A	Reverse_Complement_Seq_A	Seq_B	Lmax_Seq_A_B	Lmax	Lmax_Seq_RCA_B	Lmax
1	2	TCTTGGCCTGCTTGGTGTCT	AGACACCAAGCAGGCCAAGA	CTCACCTGTGGGAGTAACGG	CCTG	4	CACC	4
1	3	TCTTGGCCTGCTTGGTGTCT	AGACACCAAGCAGGCCAAGA	ACCGCTGCTGGGAGTAACGG	CTGCT	5	ACC	3
1	4	TCTTGGCCTGCTTGGTGTCT	AGACACCAAGCAGGCCAAGA	CCACATACGAAATGCCAACG	TGC	3	GCCAA	5
2	3	CTCACCTGTGGGAGTAACGG	CCGTTACTCCCACAGGTGAG	ACCGCTGCTGGGAGTAACGG	TGGGAGTAACGG	12	CCG	3
2	4	CTCACCTGTGGGAGTAACGG	CCGTTACTCCCACAGGTGAG	CCACATACGAAATGCCAACG	AACG	4	CCACA	5
3	4	ACCGCTGCTGGGAGTAACGG	CCGTTACTCCCAGCAGCGGT	CCACATACGAAATGCCAACG	AACG	4	TAC	3

b

Second strand diversified U6 promoter L_{max} distributions

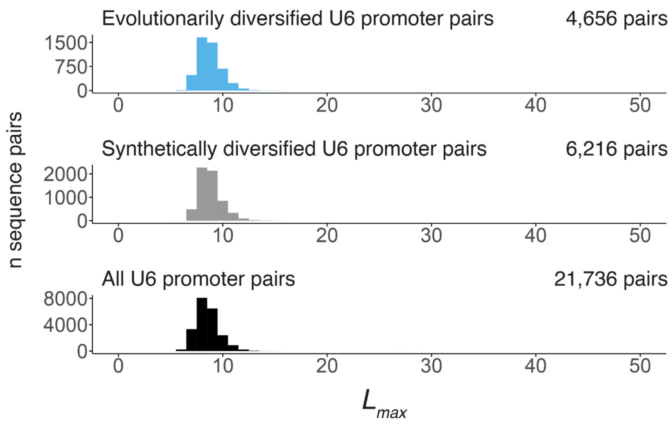

Figure S1 | L_{max} calculation example and second strand diversified U6 promoter library L_{max} distributions. a) Illustrative example of L_{max} calculations using a set of four 4 gRNA sequences (gRNAs are unrelated to the present work). A set of sequences is input, all possible pairs are generated, the longest shared repeat between each pair is identified (considering both strands), and L_{max} is reported. **b)** L_{max} distributions quantifying the maximal shared repeated length between all possible pairs of sequences for the diverse species U6 promoter library (n=97; 4,656 pairs), synthetic hRNU6-1p library (n=112; 6,216 pairs) and combined set (n=209; 21,736 pairs) for reverse complement comparisons. See **Fig. 1b** for L_{max} distributions for same strand comparisons. Note that in practice the longest shared repeat among sequence pairs in the present libraries were overwhelmingly between pairs in the same orientation due to shared functional elements.

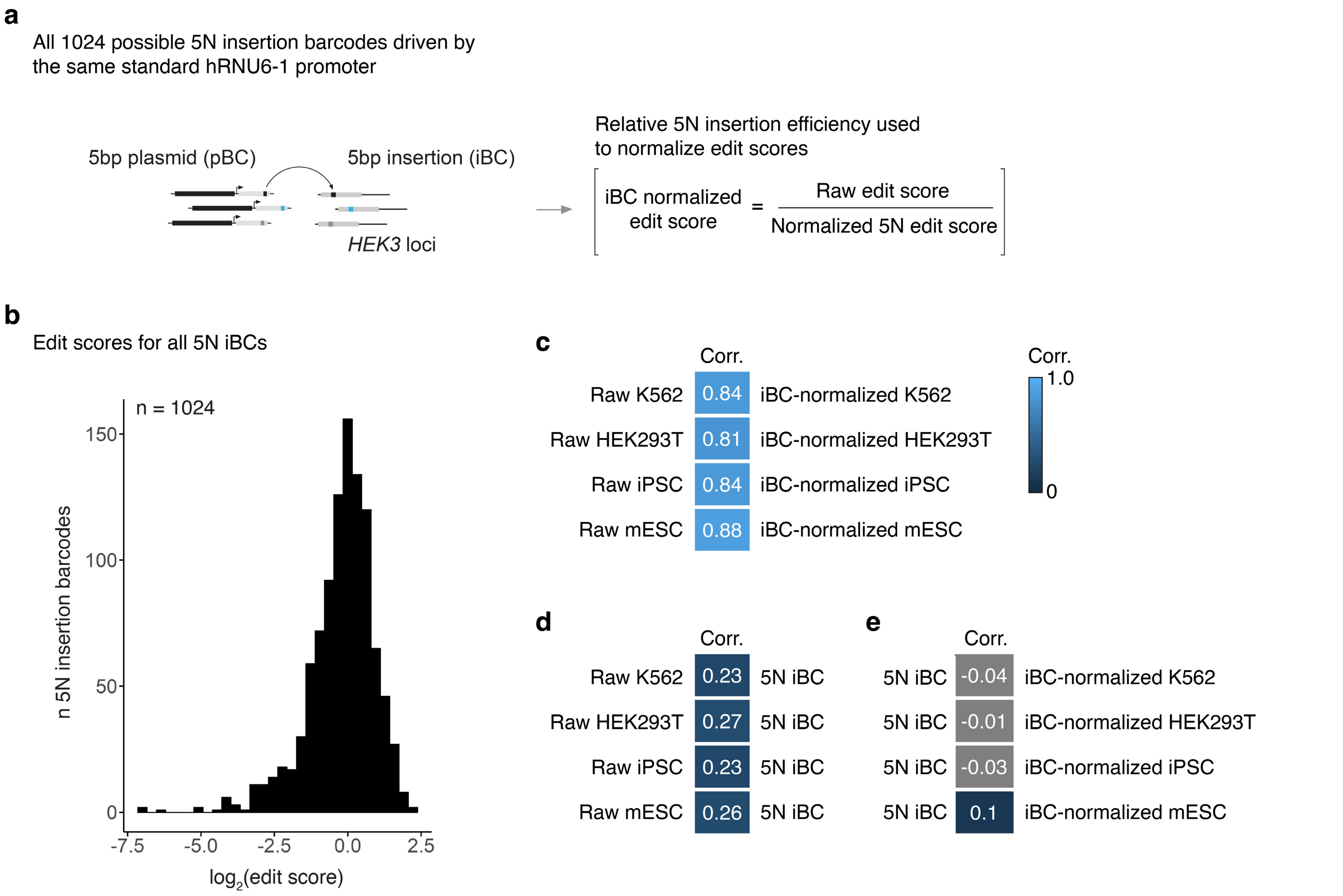

Figure S2 | Relative edit scores of all possible 5N insertion barcodes and strategy for edit score normalization. a) A library of pegRNAs programmed to insert all 1024 possible 5N iBCs was driven by the standard human RNU6-1 promoter to assess their relative insertion efficiencies. Data represent the average edit scores for each iBC across three transfection replicates in HEK293T cells. The resulting iBC edit scores, calculated by dividing each 5 bp sequence's insertion frequency at *HEK3* by its frequency in the pegRNA library, was used to normalize raw edit scores for diversified U6 promoters paired with a given 5N iBC. **b)** Distribution of edit scores for all 5N iBCs driven by the standard human RNU6-1 promoter. Edit scores for 5N iBCs were generally very similar (all 1024 barcodes drove detectable editing, and 905/1024 (88%) fell within 3-fold of the median score). **c)** Correlation between raw edit scores for diversified U6 promoters paired with different 5N iBCs and iBC-normalized edit scores. **d)** Correlation between raw edit edit scores and relative 5N insertion efficiencies. **e)** Correlation between relative 5N iBC efficiencies and iBC-normalized edit scores for diversified U6 promoters. **c-e)** Pearson correlations, calculated on barcode-normalized edit scores prior to log transformation, are shown. Accounting for relative 5N iBC insertion efficiency effectively corrected for their relatively minor influence on diversified U6 promoter edit scores.

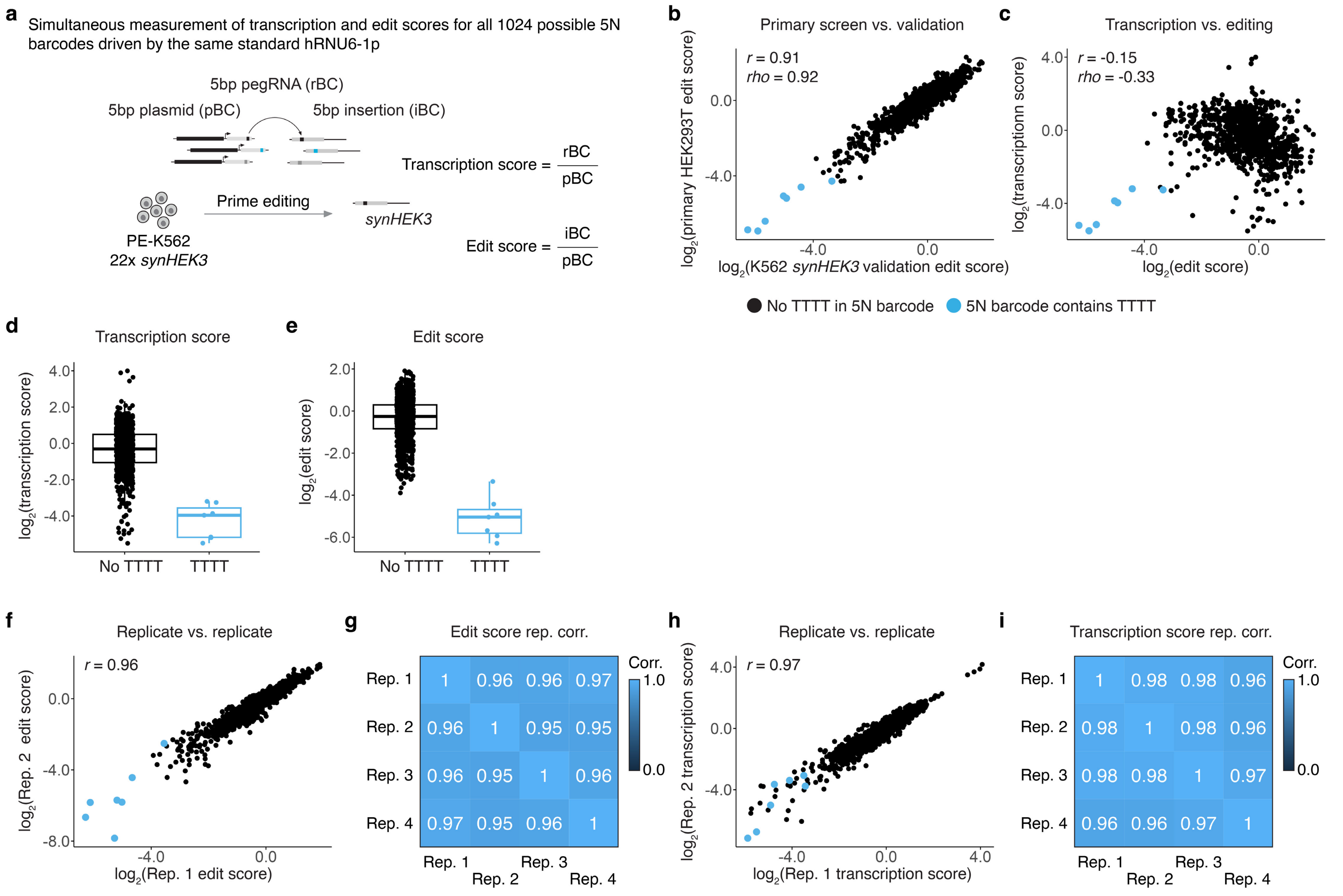

	Rep. 1	Rep. 2	Rep. 3	Rep. 4
Rep. 1	1	0.96	0.96	0.97
Rep. 2	0.96	1	0.95	0.95
Rep. 3	0.96	0.95	1	0.96
Rep. 4	0.97	0.95	0.96	1

	Rep. 1	Rep. 2	Rep. 3	Rep. 4
Rep. 1	1	0.98	0.98	0.96
Rep. 2	0.98	1	0.98	0.96
Rep. 3	0.98	0.98	1	0.97
Rep. 4	0.96	0.96	0.97	1

Figure S3 | Simultaneous measurement of pBC, rBC and iBC frequencies for all possible 1,024 5N barcodes driven by the same standard hNRU6-1p promoter, and validation of 5N edit scores in an additional cell context. a) Workflow of experiment to compare edit scores vs. transcriptional scores for all 5N barcodes. A library containing all possible 1,024 5N barcodes driven by the same standard human hRNU6-1p promoter was delivered to K562 cells engineered to harbor a constitutively expressed prime editor and ~22 *synHEK3* target sites. DNA and RNA were harvested, and then plasmid barcodes (pBC; DNA), pegRNA barcodes (rBC; RNA), and insertion barcodes (iBCs; DNA) were amplified and sequenced. Edit scores were defined as the frequency of an insertional barcode (iBC) at the genomic target site divided by the frequency of the same barcode in the plasmid library (pBC). Transcription scores were defined as the frequency of a transcribed pegRNA barcode (rBC) divided by the frequency of the same barcode in the plasmid library (pBC), further normalized by independently measured barcode transcriptional efficiencies. **b)** Reproducibility of edit scores in the primary screen (HE293T, *HEK3*) vs. independent validation experiment (*K562*, *synHEK3*) depicted in panel **a**. Pearson and Spearman correlation coefficients, calculated between edit scores prior to log transformation, are listed. **c)** Comparison of transcription scores vs. edit scores. Pearson and Spearman correlation coefficients, calculated between log-transformed transcription scores and edit scores, are listed. **d-e)** Boxplots of log-transformed transcription scores **(d)** and log-transformed edit scores **(e)** for 5N barcodes that do not contain (left) or do contain (right) a “TTTT” polyT termination tract. **d-e)** Boxes represent the 25th and 75th percentiles, box centre line represents the median. Whiskers extend from hinge to 1.5 times the interquartile range. **f-i)** Pearson correlation coefficients, calculated between edit scores prior to log transformation, are listed. **f)** Reproducibility of edit scores between exemplary replicates. **g)** Heatmap of Pearson correlation coefficients between edit scores for pairs of transfection replicates. **h)** Reproducibility of transcription scores between exemplary replicates. **i)** Heatmap of Pearson correlation coefficients between transcription scores for pairs of transfection replicates.

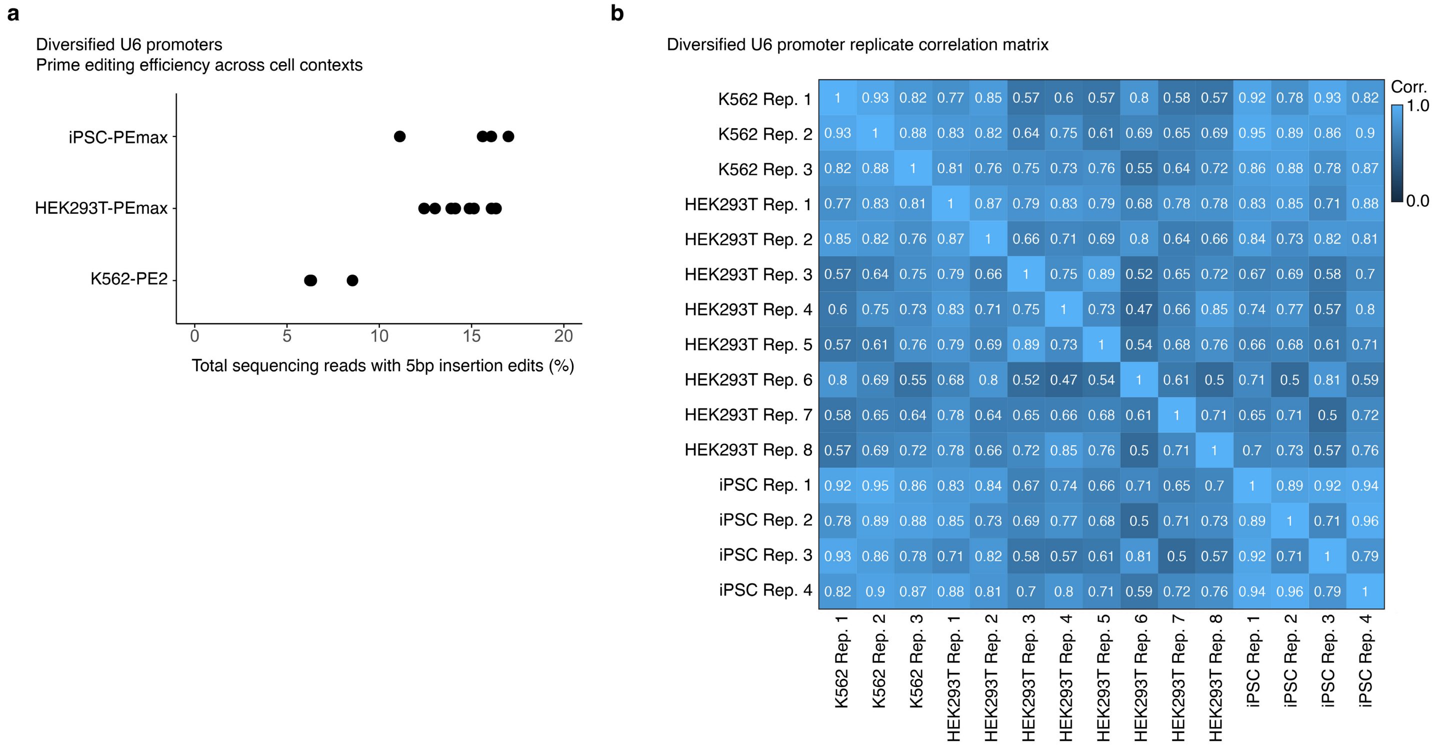

Figure S4 | U6 promoter diversification experiment: prime editing efficiencies and edit score replicate correlations across cellular contexts. a) Prime editing efficiency of the diversified U6 promoter library across human cellular contexts. Cell lines expressing an optimized PEmax construct displayed higher editing scores than the K562 line expressing the original PE2 construct, as expected. **b)** Correlation of diversified U6 promoter edit scores across cellular contexts for individual transfection replicates. Pearson correlations, calculated on barcode-normalized edit scores prior to log transformation, are shown.

a

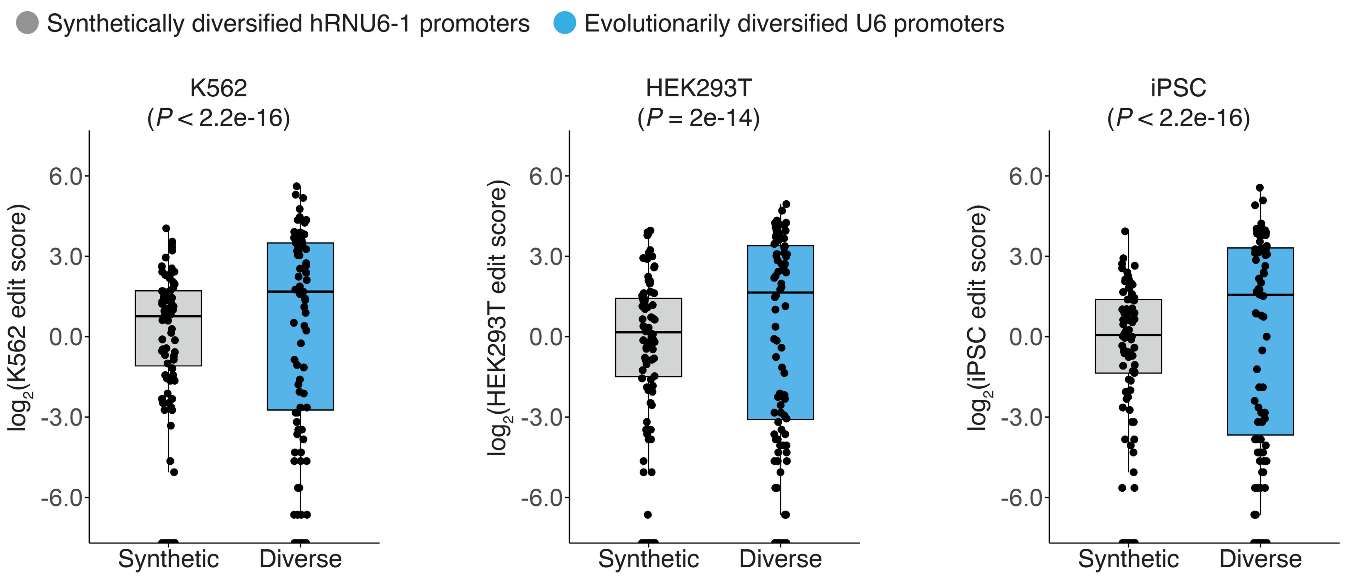

Figure S5 | Comparison of synthetically and evolutionarily diversified U6 promoters. a) Comparison of edit score distributions for synthetically and evolutionarily diversified U6 promoters in three human cellular contexts. Evolutionarily diversified U6 promoters exhibited a greater diversity of activity levels. *P*-values from an *F*-test for equality of variance are shown above. Boxes represent the 25th and 75th percentiles, box centre line represents the median. Whiskers extend from hinge to 1.5 times the interquartile range.

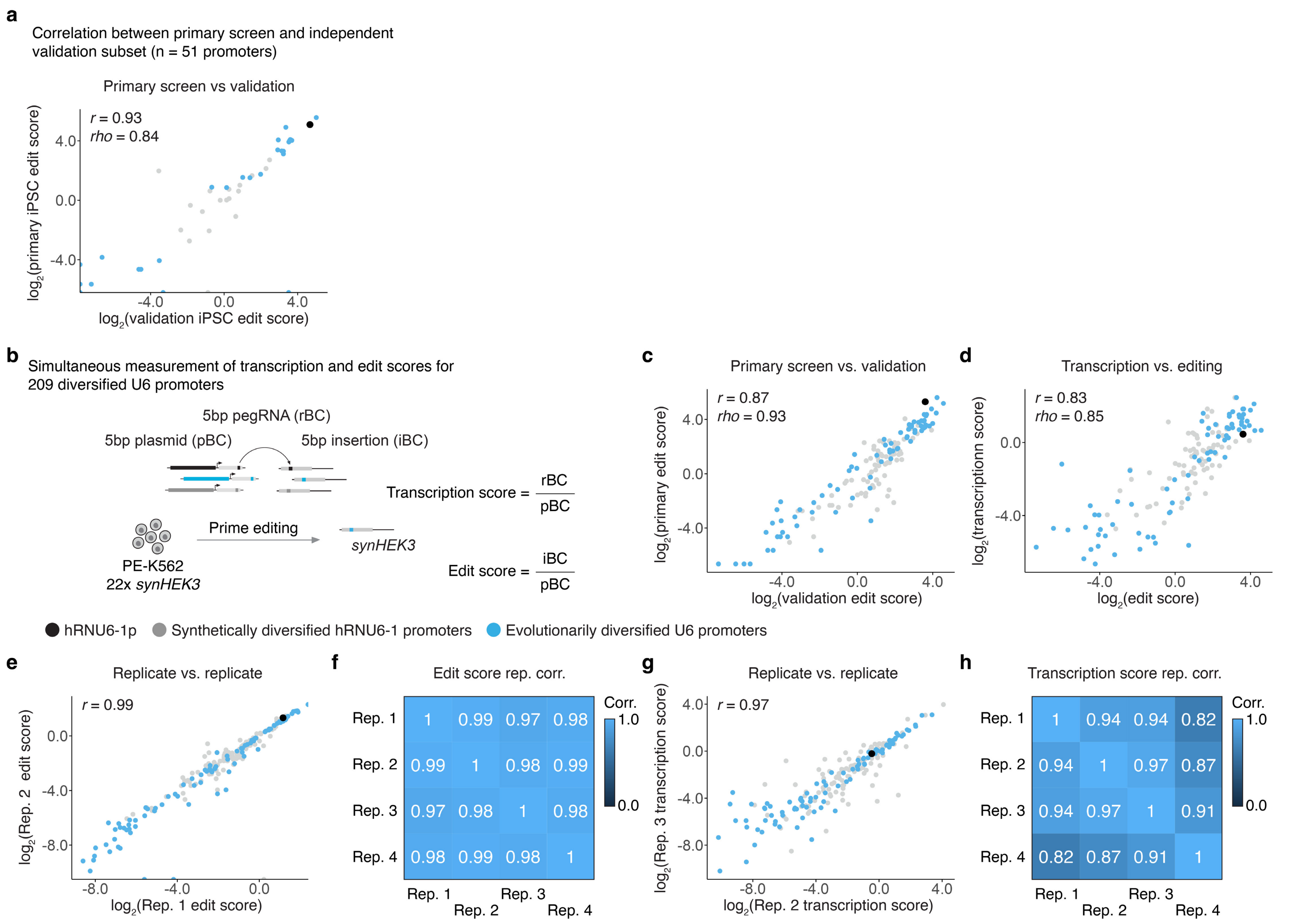

Figure S6 | Independent validation of diversified U6 promoters, either in a subpool or via direct comparison of editing to transcriptional output. a) Reproducibility of edit scores in PEmax-iPSCs for 51 diversified U6 promoters representing a range of activity levels. Edit scores were highly correlated for the primary screen vs. a validation experiment in which these 51 diversified U6 promoters were independently cloned and tested as a subpool. Pearson correlation coefficients, calculated between barcode-normalized edit scores prior to log transformation, are listed. **b)** Workflow of experiment to compare edit scores vs. transcriptional scores. 209 diversified Pol III promoters were delivered to K562 cells engineered to harbor a constitutively expressed prime editor and ~22 *synHEK3* target sites. DNA and RNA were harvested, and then plasmid barcodes (pBC; DNA), pegRNA barcodes (rBC; RNA), and insertion barcodes (iBCs; DNA) were amplified and sequenced. Edit scores were defined as the frequency of an insertional barcode (iBC) at the genomic target site divided by the frequency of the same barcode in the plasmid library (pBC), further normalized by independently measured barcode insertional efficiencies. Transcription scores were defined as the frequency of a transcribed pegRNA barcode (rBC) divided by the frequency of the same barcode in the plasmid library (pBC), further normalized by independently measured barcode transcriptional efficiencies. **c)** Reproducibility of edit scores in the primary screen vs. independent validation experiment depicted in panel **b**. Pearson and Spearman correlation coefficients, calculated between barcode-normalized edit scores prior to log transformation, are listed. **d)** Comparison of transcription scores vs. edit scores. Pearson and Spearman correlation coefficients, calculated between log-transformed transcription scores and edit scores, are listed. **e-h)** Pearson correlation coefficients, calculated between edit scores prior to log transformation, are listed. **e)** Reproducibility of edit scores between exemplary replicates. **f)** Heatmap of Pearson correlation coefficients between edit scores for pairs of transfection replicates. **g)** Reproducibility of transcription scores between exemplary replicates. **h)** Heatmap of Pearson correlation coefficients between transcription scores for pairs of transfection replicates.

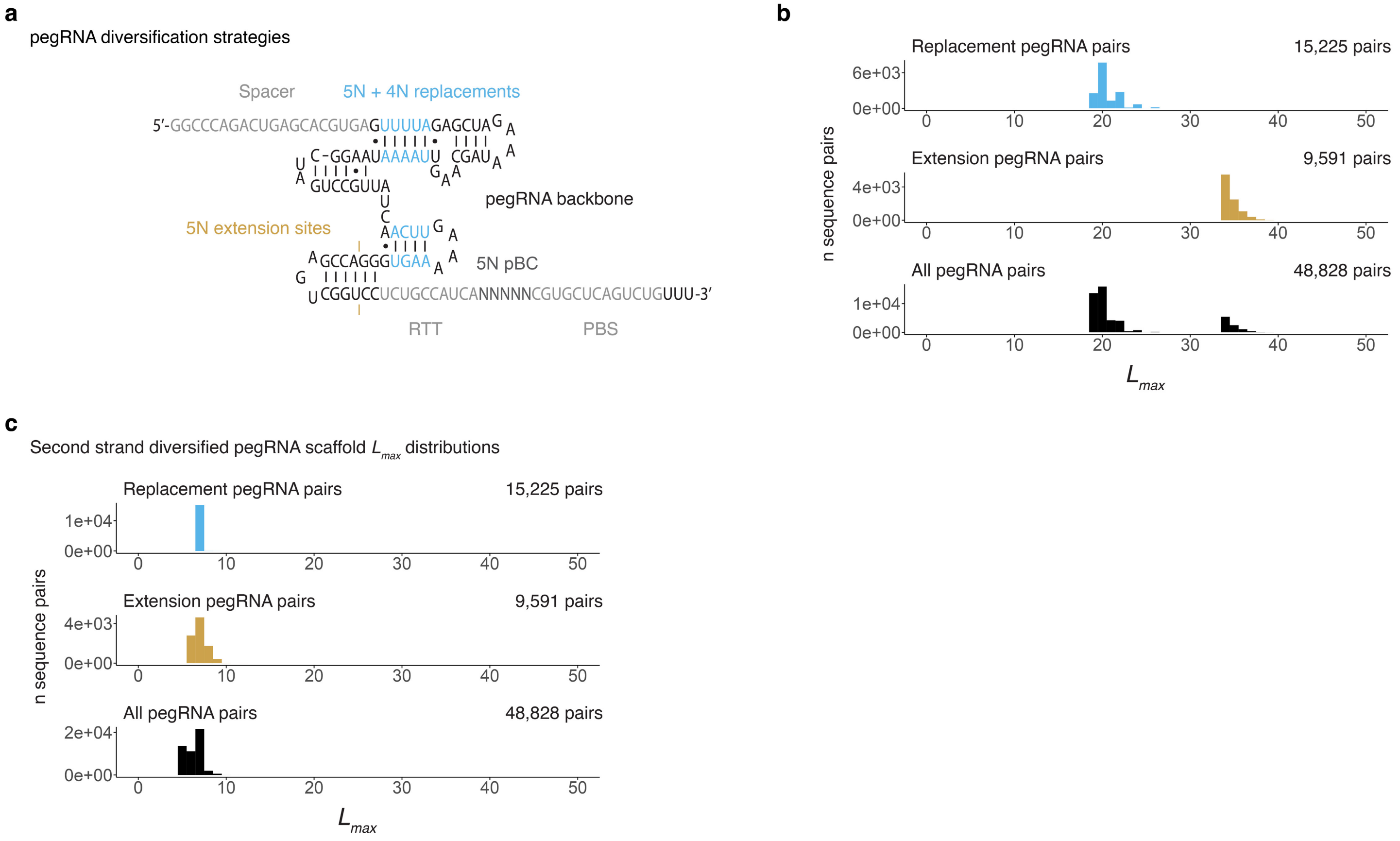

Figure S7 | Diversified pegRNA library design and L_{max} distributions. a) Predicted structure of a *HEK3* targeting pegRNA with a standard scaffold sequence programmed to insert a 5N iBC at the *HEK3* locus. Sites where random, complementary 5N and 4N replacements and extensions were introduced to diversify scaffold sequences are shown. **b)** L_{max} distributions quantifying the maximal shared repeated length between all possible pairs of sequences for the replacement scaffolds (n=174, 15,225 pairs), and extension scaffolds (n=138; 9,591 pairs) and combined set including the standard sequence (n=313, 48,828 pairs), in the same orientation. **c)** L_{max} distributions as in panel **b** but for reverse complement comparisons.

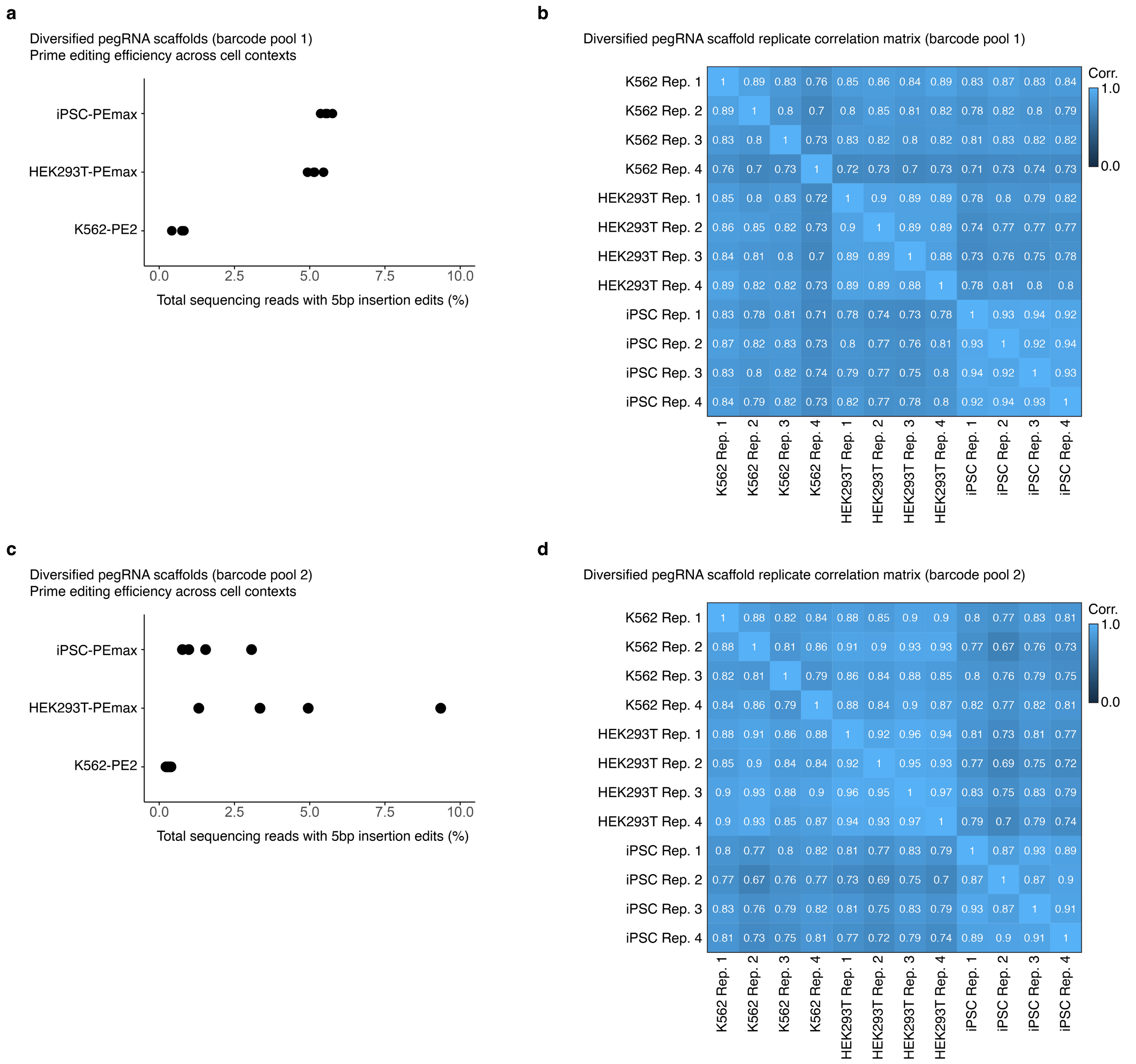

Figure S8 | Scaffold diversification experiment: prime editing efficiencies and edit score replicate correlations across cellular contexts. a) Prime editing efficiencies of the diversified pegRNA scaffold library across cellular contexts for the first independent barcode pool. Cell lines expressing an optimized PEmax construct displayed higher editing scores than the K562 line expressing the original PE2 construct, as expected. **b)** Correlation of diversified pegRNA scaffold edit scores across cell contexts for individual transfection replicates of the first independent barcode pool. Pearson correlations, calculated on barcode-normalized edit scores prior to log transformation, are shown. **c)** Same as panel **a** but for the second independent barcode pool. **d)** Same as panel **b** but for the second independent barcode pool.

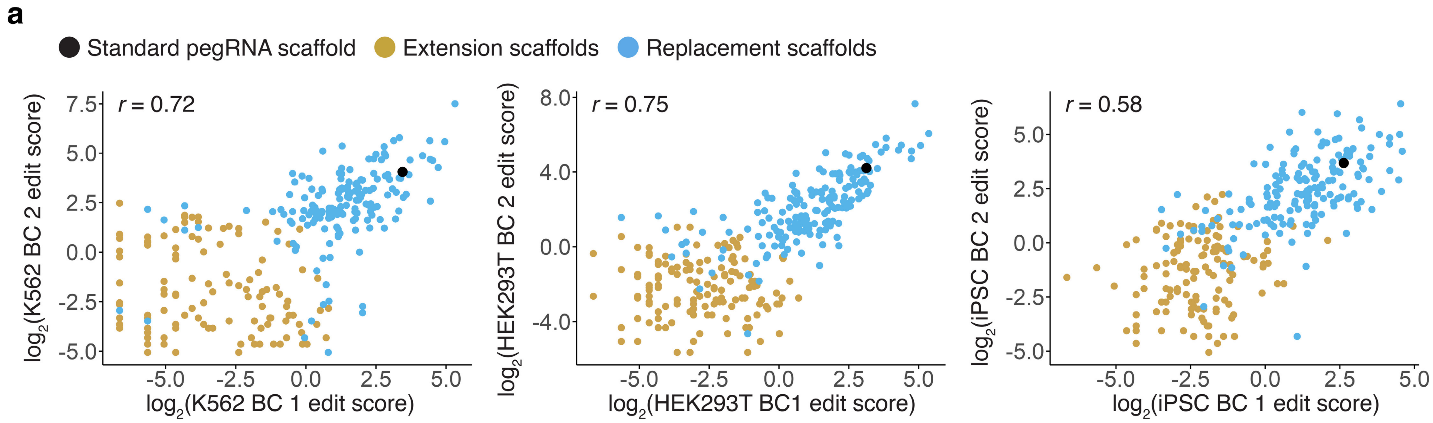

Figure S9 | Diversified pegRNA edit score correlations between independent barcode pools. a) Reproducibility of diversified pegRNA scaffold edit scores generated from two independent barcode pools, in each of three human cellular contexts. Pearson correlations, calculated on barcode-normalized edit scores prior to log-transformation, are listed.

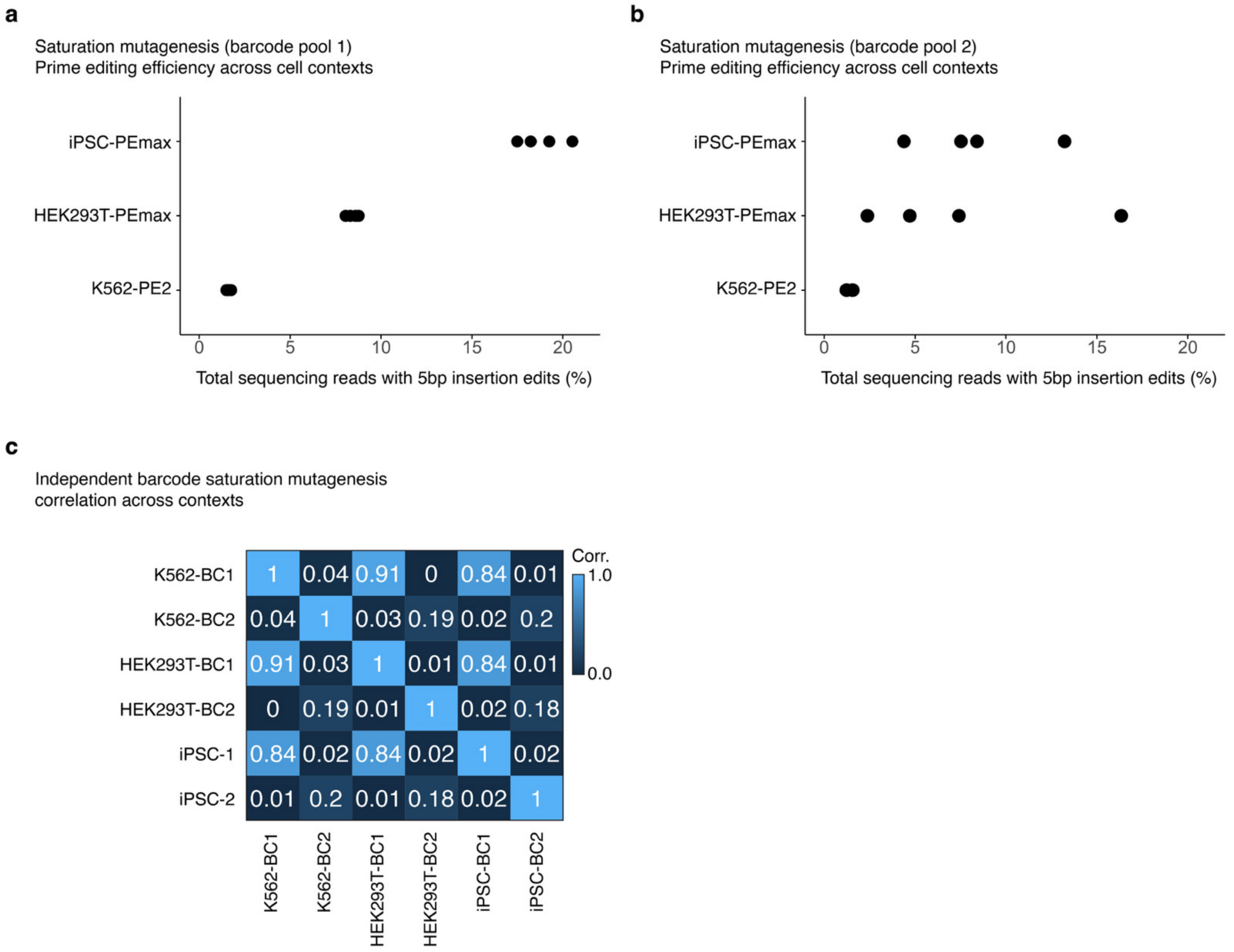

Figure S10 | Saturation mutagenesis experiment: prime editing efficiencies and edit score correlations across cellular contexts. a) Prime editing efficiencies of the miniaturized hRNU6-1p-pegRNA cassette saturation mutagenesis library across cellular contexts for the first independent barcode pool. Cell lines expressing an optimized PEmax construct displayed higher editing scores than the K562 line expressing the original PE2 construct, as expected. **b)** Same as panel **a** but for the second independent barcode pool. **c)** Independent barcode correlation of saturation mutagenesis variant edit scores across cellular contexts. Pearson correlations based on edit scores for each independent barcode and context prior to log transformation, are shown.

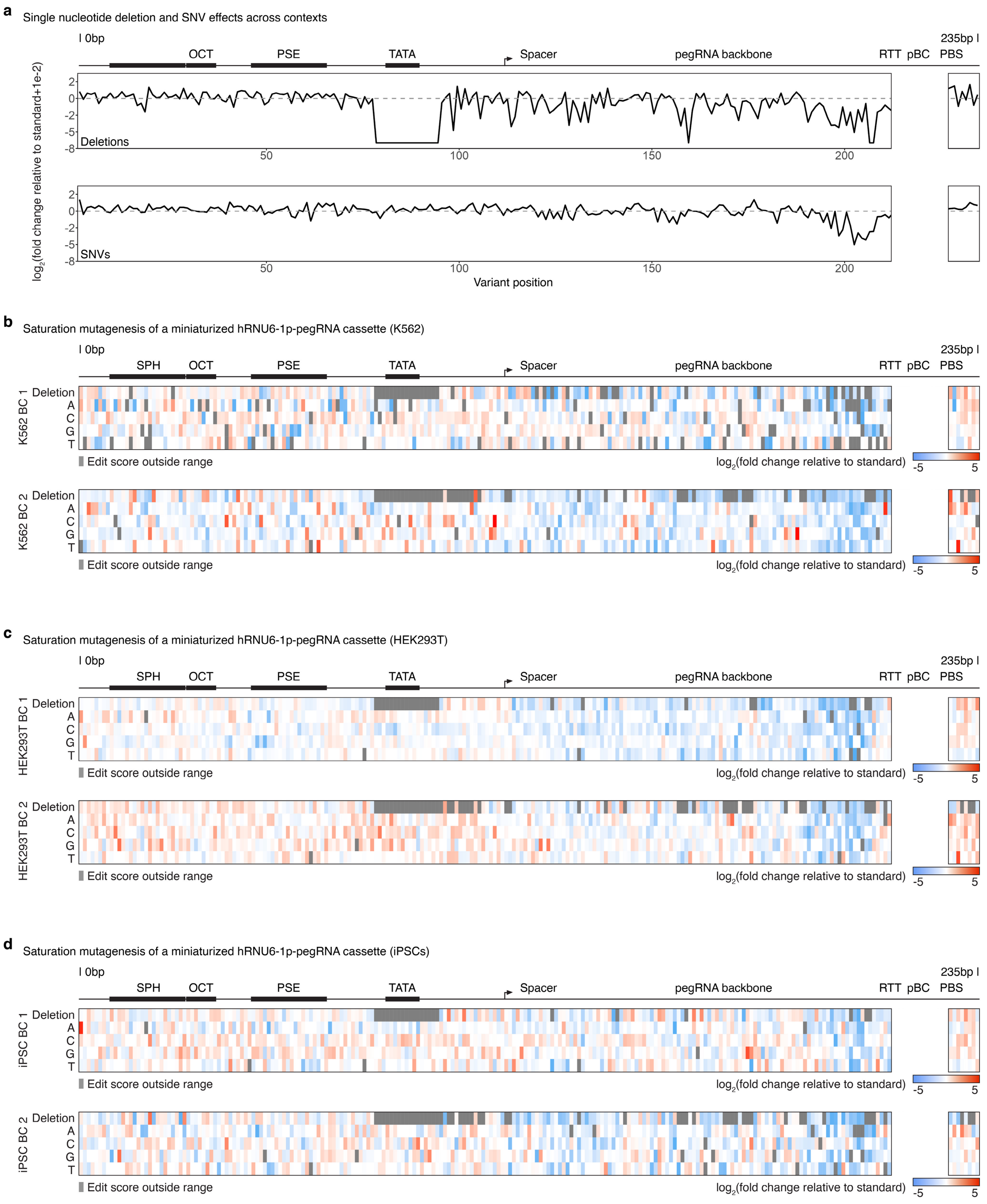

Figure S11 | Saturation mutagenesis variant effect maps of miniaturized hRNU6-1p-pegRNA cassettes across cellular contexts. a) Effects of single nucleotide deletions and SNVs across cellular contexts. Median log-transformed fold-change in edit scores relative to the wildtype cassette across cellular contexts for single nucleotide deletions (top) and average across all SNVs (bottom) are shown. **b)** Log-transformed fold-change in edit scores relative to the wildtype cassette for the first (top) and second (bottom) independent barcode pools are shown. **c)** Same as panel **b** but experiment conducted in HEK293T cells. **d)** Same as panel **b** but experiment conducted in iPSCs. Edit scores were not calculated for the unboxed region surrounding the pBC, as exact matches spanning this region were required for edit quantification.

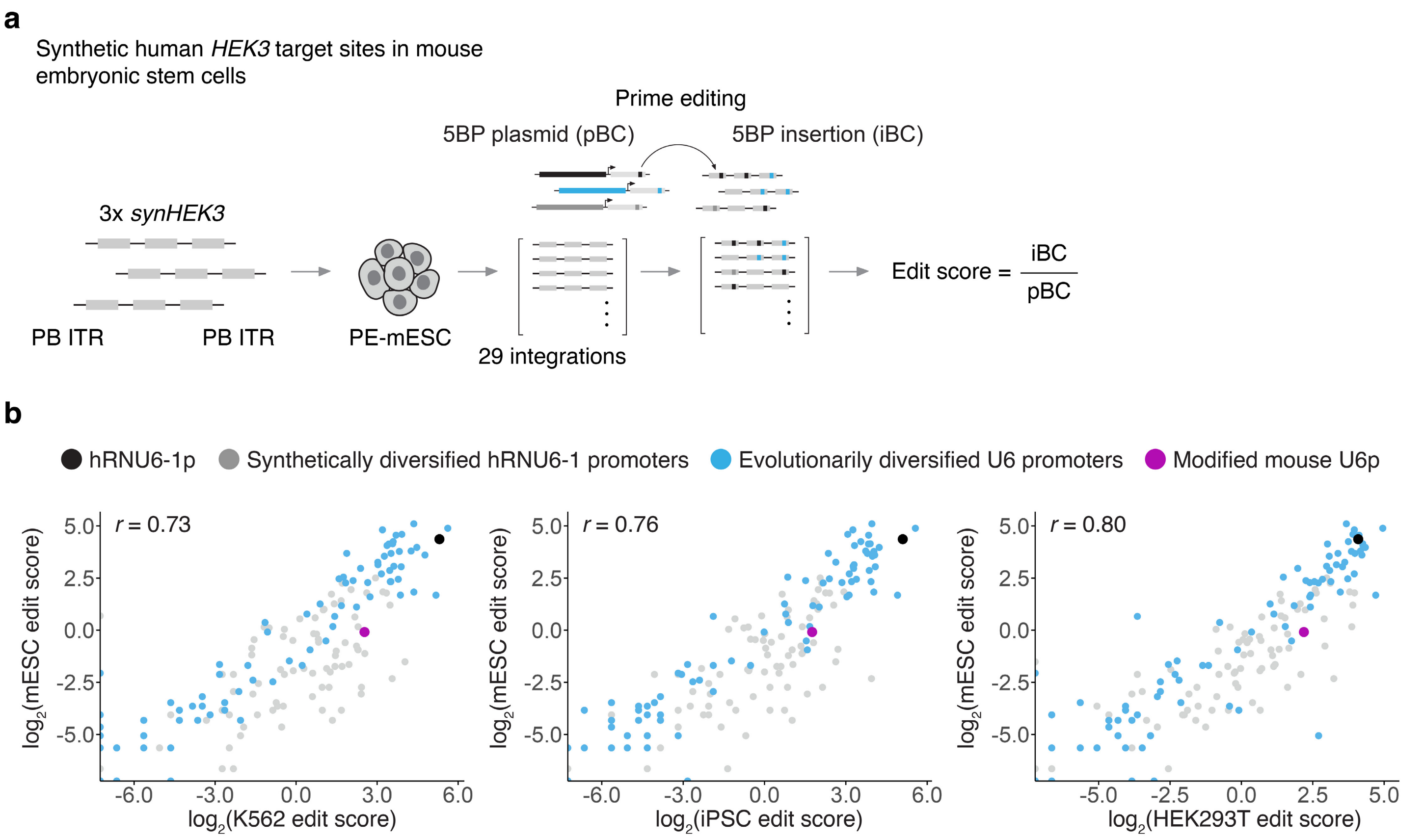

Figure S12 | Diversified U6 promoters exhibit consistent activities in human vs. mouse contexts. a) PEmax and synthetic human *HEK3* target sites (*synHEK3*) were introduced to mESCs via piggyBac transposition. A monoclonal line with PEmax and an estimated 87 *synHEK3* targets was isolated (29 integrations x 3 *synHEK3* targets per integration). Diversified U6 promoters were assessed for their ability to drive genome editing in mESCs, once again using a multiplex prime editing functional assay. **b)** Pairwise comparison of log-transformed edit scores between three human (x-axes) and one mouse (y-axis) cellular contexts. Pearson correlations, calculated on barcode-normalized edit scores prior to log transformation, are listed.

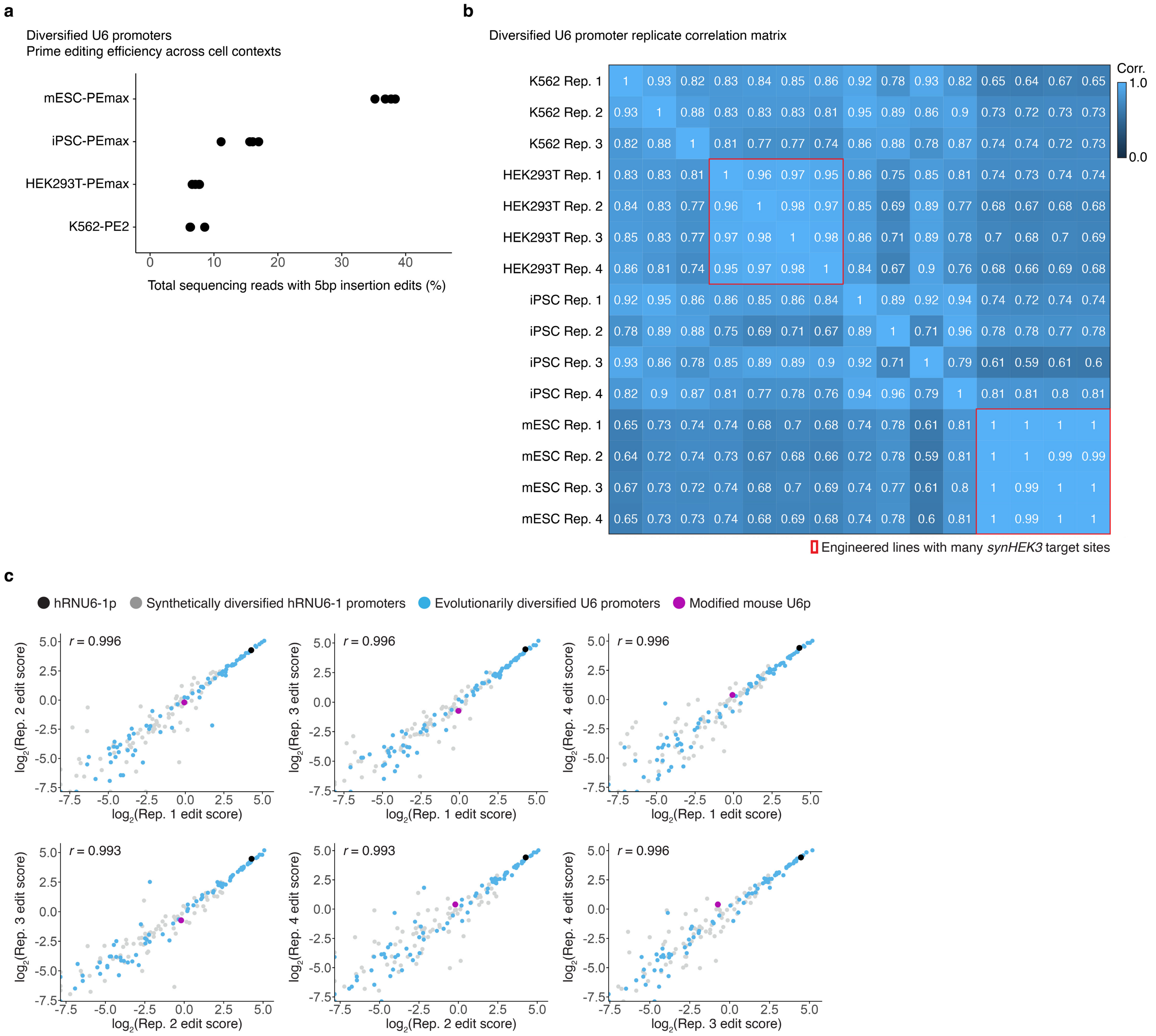

Figure S13 | Extension of the U6 promoter diversification experiment to mESCs: prime editing efficiencies and edit score replicate correlations. a) Prime editing efficiencies of the diversified U6 promoter library in mESCs vs. human cell contexts. The editing efficiencies were considerably higher in the mESC line, possibly because it was engineered to harbor many *synHEK3* target sites per cell, or possibly for other reasons. **b)** Heatmaps of Pearson correlation coefficients between pairs of transfection replicates, calculated between edit scores prior to log transformation, within and across cellular contexts. Red squares highlight high Pearson correlation coefficients between technical replicates of experiments conducted in HEK293T or mESC cell lines bearing many *synHEK3* target sites per cell. Technical replicate comparisons with Pearson correlation coefficients >0.995 are rounded to 1 in the visualization. **c)** Reproducibility of edit scores between pairs of transfection replicates for experiments conducted in mESCs. Pearson correlation coefficients, calculated on barcode-normalized edit scores prior to log transformation, are listed.

a

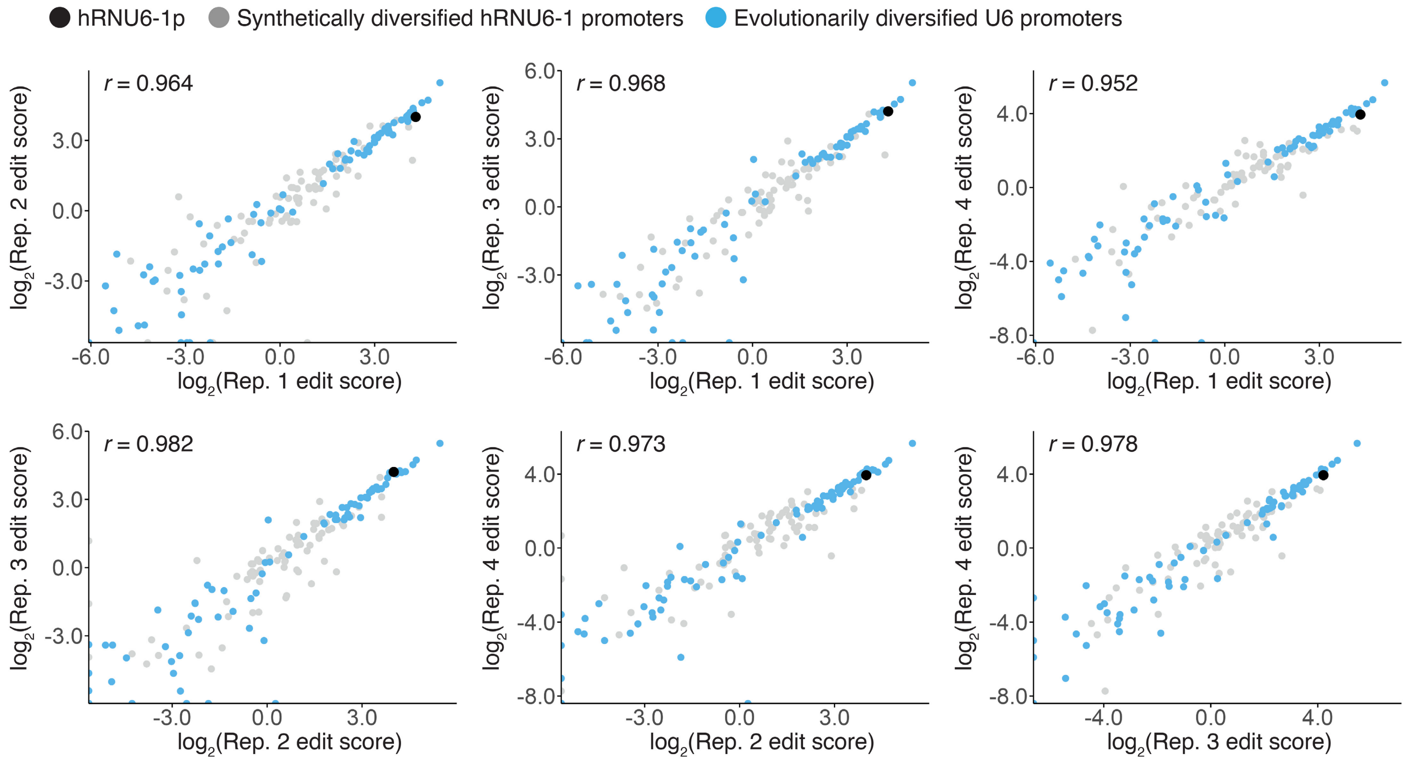

Figure S14 | Reproducibility of U6 promoter diversification experiment conducted in HEK239T cell line harboring many *synHEK3* target sites. a) Reproducibility of edit scores between pairs of transfection replicates for experiments conducted in a new monoclonal HEK293T line harboring ~146 *synHEK3* target sites. Pearson correlation coefficients, calculated on barcode-normalized edit scores prior to log transformation, are listed.

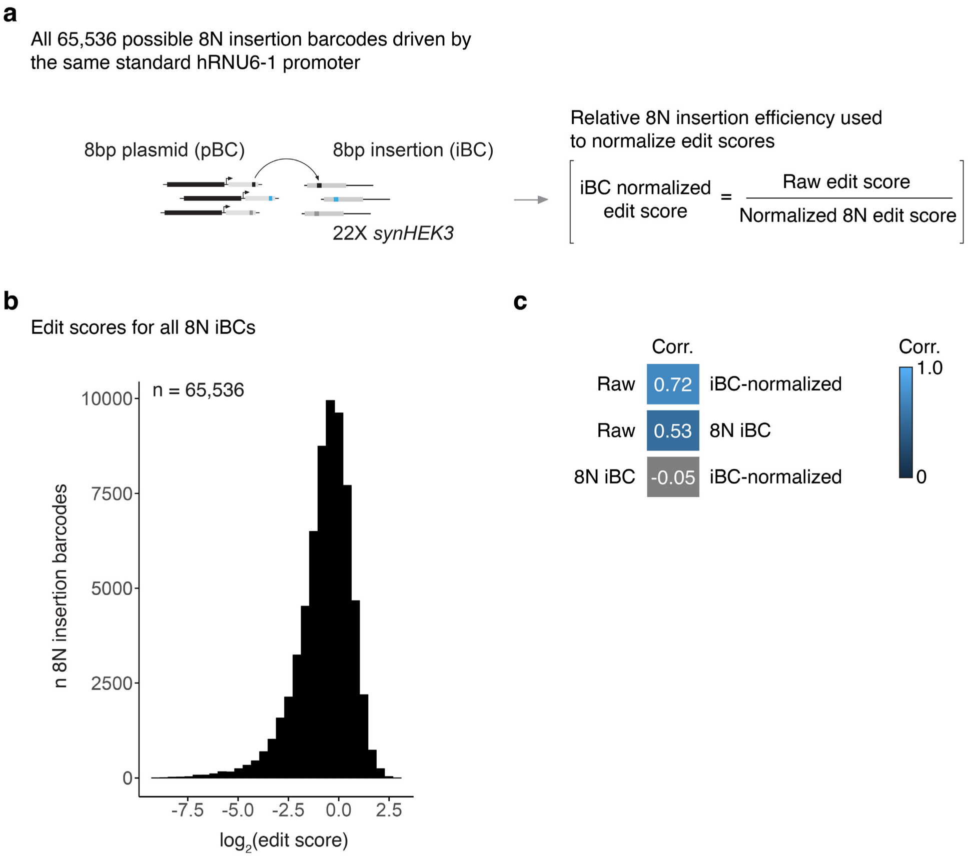

Figure S15 | Relative edit scores of all possible 8N insertion barcodes and strategy for edit score normalization. a) A library of pegRNAs programmed to insert all 65,536 possible 8N iBCs was driven by the standard human RNU6-1 promoter to assess their relative insertion efficiencies. The resulting iBC edit scores, calculated by dividing each 8 bp sequence's insertion frequency at *HEK3* by its frequency in the pegRNA library, was used to normalize raw edit scores for diversified U6 promoters paired with a given 8N iBC. **b)** Distribution of edit scores for all 8N iBCs driven by the standard human RNU6-1 promoter. **c)** Correlation between raw edit scores for diversified U6 promoters paired with different 8N iBCs and iBC-normalized edit scores. Pearson correlation coefficients, calculated on edit scores prior to log transformation, are listed. Accounting for relative 8N iBC insertion efficiency effectively corrected for their influence on diversified U6 promoter edit scores.

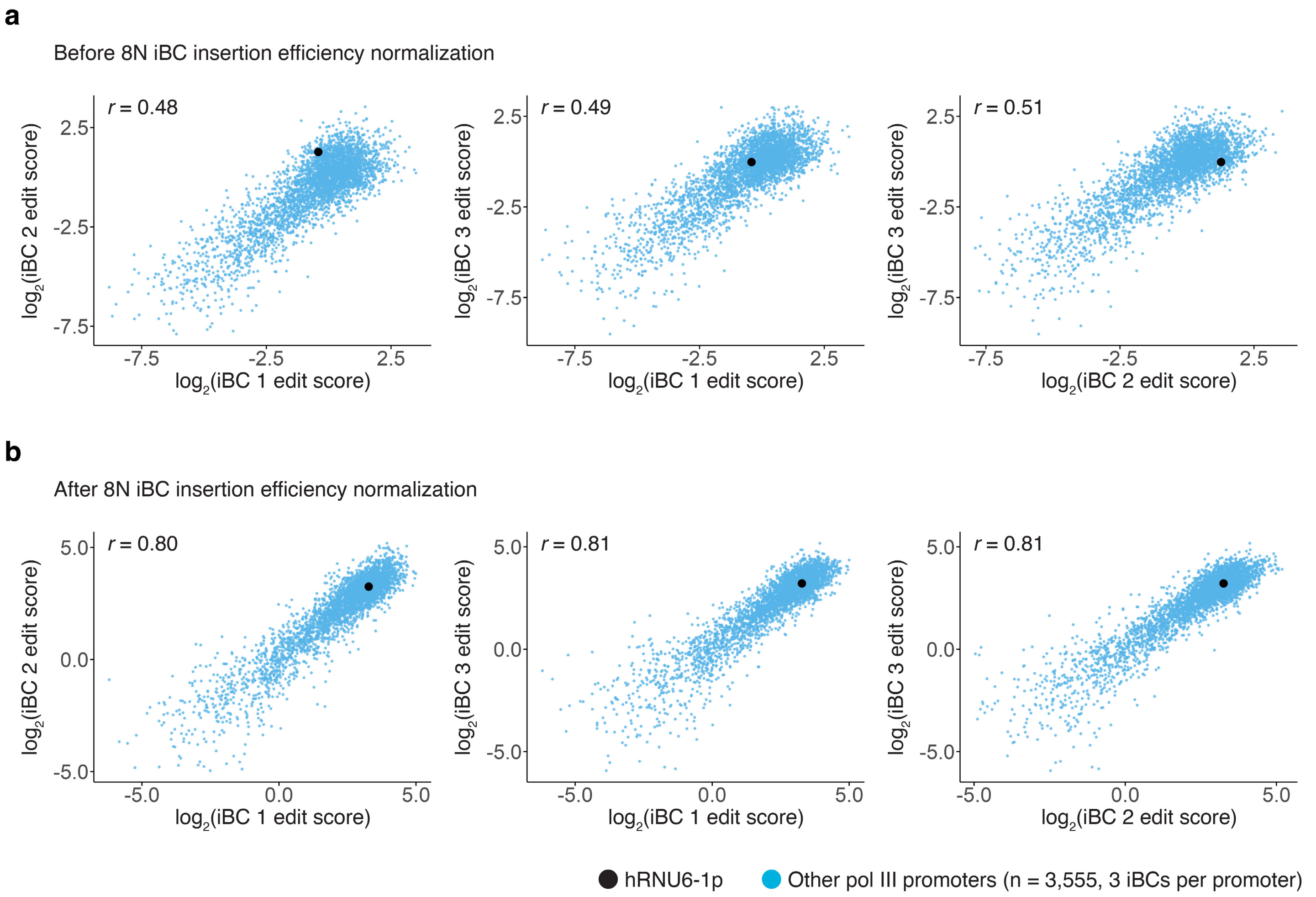

Figure S16 | Edit score correlations for independent iBCs across the 3,566 evolutionarily diversified Pol III promoters. a-b) Reproducibility of raw edit scores across iBCs either before (panel **a**) or after (panel **b**) correction for relative barcode insertion efficiencies. For all plots, Pearson correlations, calculated on barcode-normalized edit scores prior to log transformation, are listed. Accounting for barcode insertion efficiency substantially improved concordance.

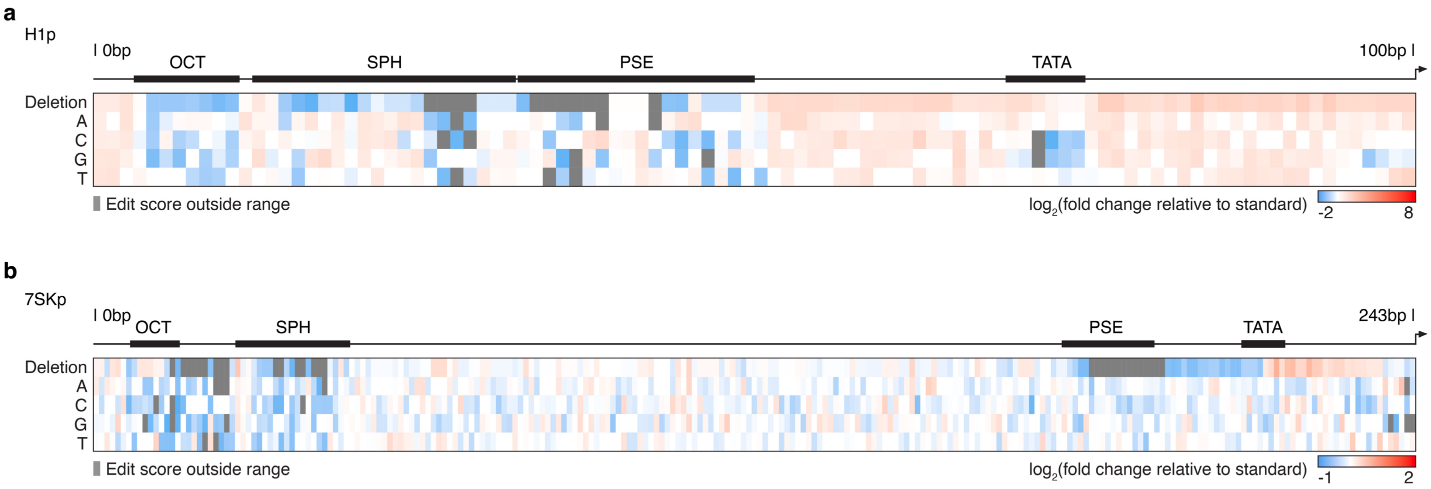

Figure S17 | Variant effect maps for human H1 and 7SK promoters. a) Variant effect map for the human H1 promoter. **b)** Variant effect map for the human 7SK promoter. Log-transformed fold-changes in edit scores relative to the wildtype human H1 or 7SK promoter are shown. Human H1 and 7SK promoters are relatively tolerant to single nucleotide deletions in the TATA box.

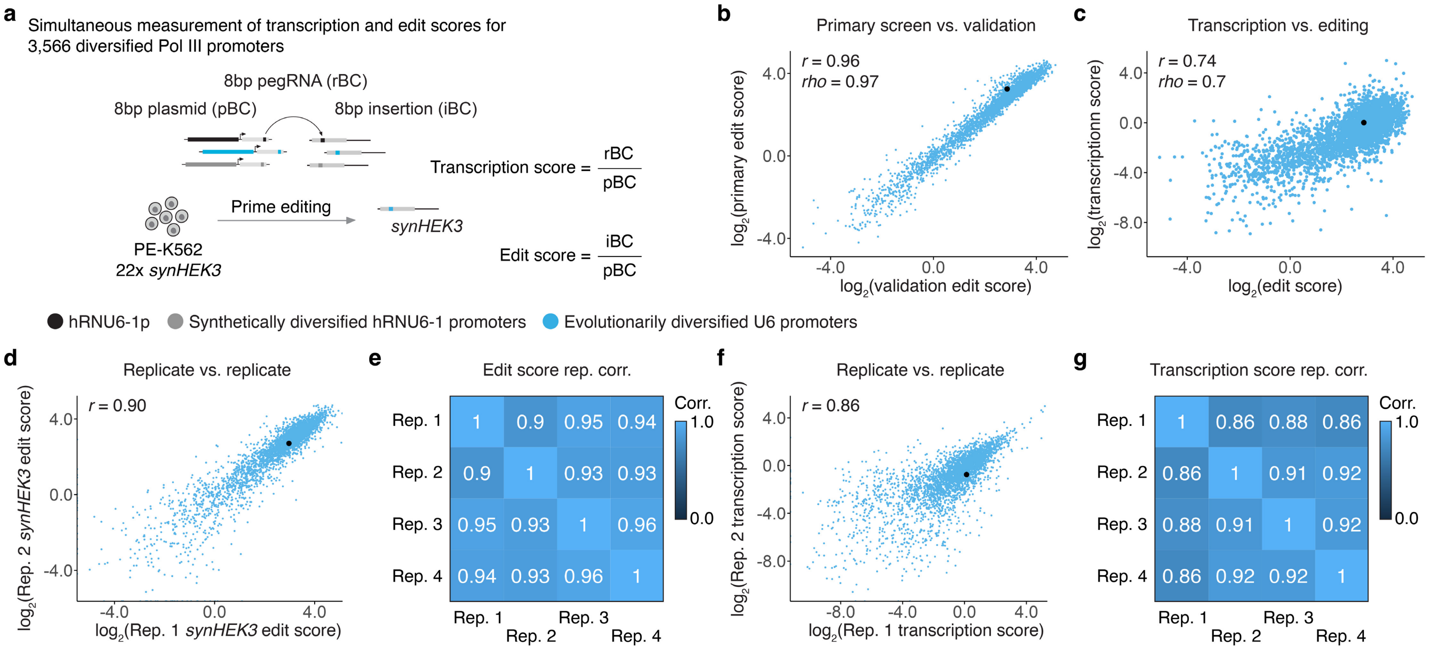

Figure S18 | Independent validation of diversified Pol III promoters via direct comparison of editing to transcriptional output. a) Workflow of experiment to compare edit scores vs. transcriptional scores. 3,566 diversified Pol III promoters were delivered to K562 cells engineered to harbor a constitutively expressed prime editor and ~22 *synHEK3* target sites. DNA and RNA were harvested, and then plasmid barcodes (pBC; DNA), pegRNA barcodes (rBC; RNA), and insertion barcodes (iBCs; DNA) were amplified and sequenced. Edit scores were defined as the frequency of an insertional barcode (iBC) at the genomic target site divided by the frequency of the same barcode in the plasmid library (pBC), further normalized by independently measured barcode insertional efficiencies. Transcription scores were defined as the frequency of a transcribed pegRNA barcode (rBC) divided by the frequency of the same barcode in the plasmid library (pBC), further normalized by independently measured barcode transcriptional efficiencies. **c)** Reproducibility of edit scores in the primary screen vs. independent validation experiment depicted in panel **b**. Pearson and Spearman correlation coefficients, calculated between barcode-normalized edit scores prior to log transformation, are listed. **d)** Comparison of transcription scores vs. edit scores. Pearson and Spearman correlation coefficients, calculated between log-transformed transcription scores and edit scores, are listed. **e-h)** Pearson correlation coefficients, calculated between edit scores prior to log transformation, are listed. **e)** Reproducibility of edit scores between exemplary replicates. **f)** Heatmap of Pearson correlation coefficients between edit scores for pairs of transfection replicates. **g)** Reproducibility of transcription scores between exemplary replicates. **h)** Heatmap of Pearson correlation coefficients between transcription scores for pairs of transfection replicates.

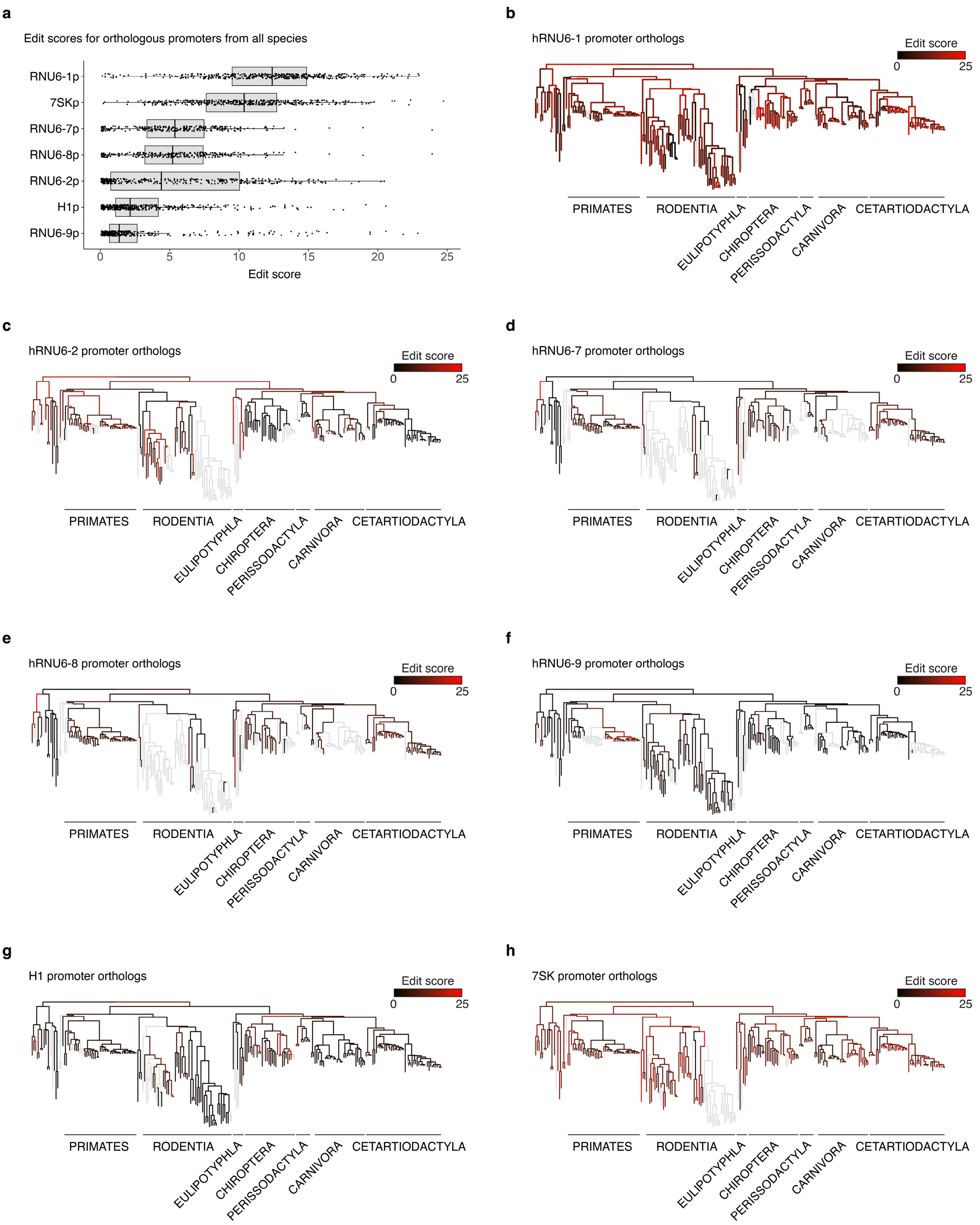

Figure S19 | The functional landscape of ancestral and extant mammalian Pol III promoters in human cells evolution **a)** Edit scores distributions for ancestral and extant mammalian orthologs of various human Pol III promoters. Boxes represent the 25th and 75th percentiles, box centre line represents the median. Whiskers extend from hinge to 1.5 times the interquartile range. **b-h)** Edit scores from panel **a** plotted onto phylogenetic trees to visualize the evolution of functional activity of for orthologs of hRNU6-1p (**b**), hRNU6-2p (**c**), hRNU6-7p (**d**), hRNU6-8p (**e**), hRNU6-9p (**f**), H1p (**g**) and 7SKp (**h**). Gray lines represent ancestrally reconstructed or extant sequenced genomes for which there was no orthologous Pol III promoter identified.

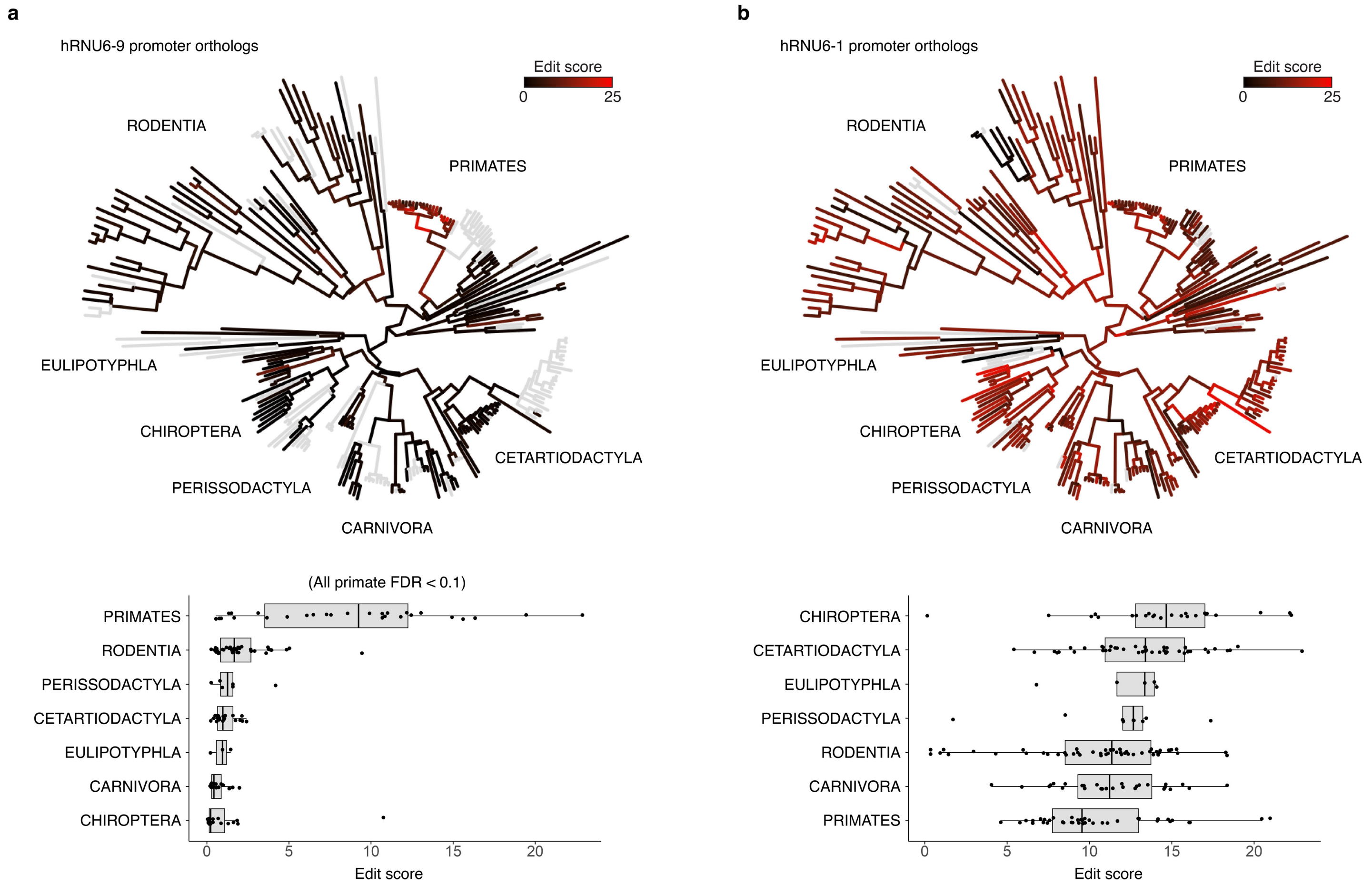

Figure S20 | Ancestral and extant mammalian Pol III promoters that are highly active in human cells. a) (top) Phylogenetic tree depicting the evolutionary relationships and edit scores of extant and ancestral hRNU6-9p orthologs. (bottom) Comparison of edit scores for hRNU6-9p orthologs from major orders with more than five extant species. Boxes represent the 25th and 75th percentiles, box centre line represents the median. Whiskers extend from hinge to 1.5 times the interquartile range. *P*-values from a Wilcoxon rank-sum test with a Benjamini-Hochberg false-discovery rate (FDR) control. Gray lines represent ancestrally reconstructed or extant sequenced genomes for which there was no orthologous Pol III promoter identified. **b)** Same as panel **a** but for extant and ancestral hRNU6-1p orthologs.

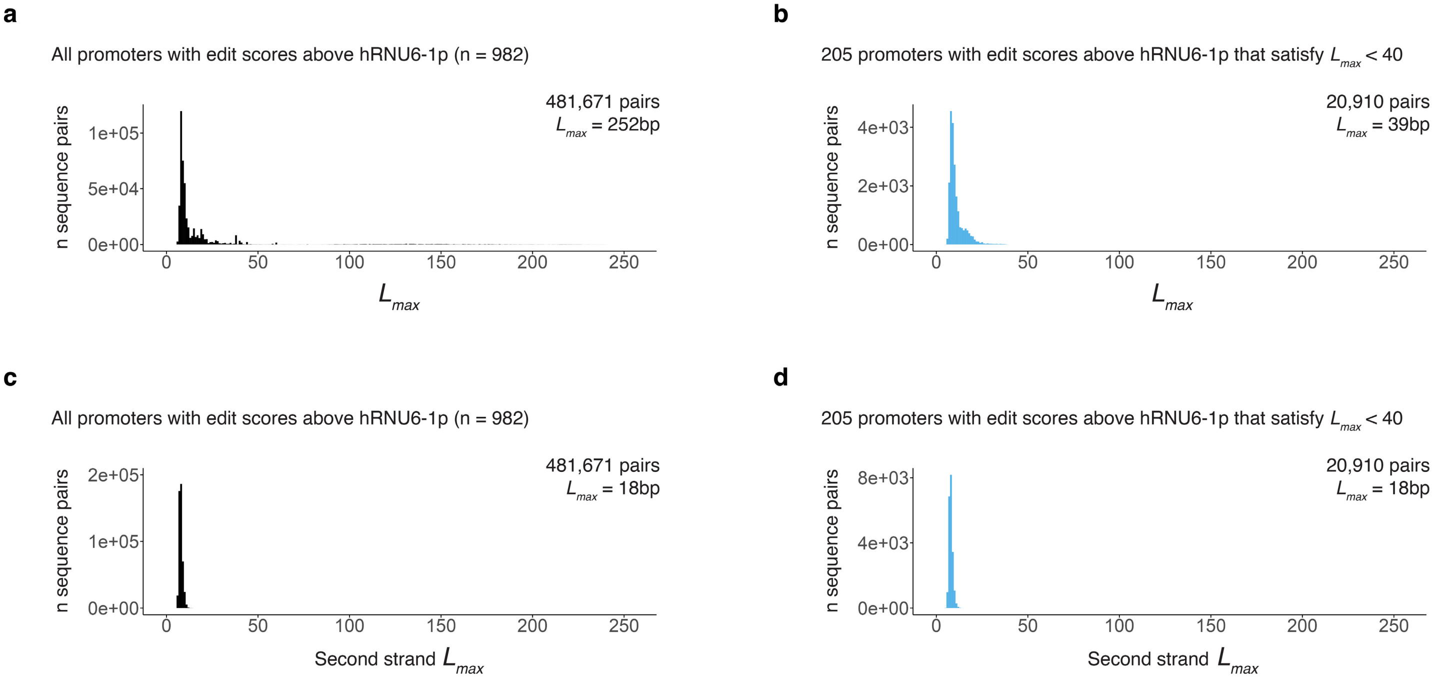

Figure S21 | L_{max} distributions for Pol III promoters with edit scores above standard. a) L_{max} distributions quantifying the maximal shared repeated length between all possible pairs of sequences, in the same orientation, for all Pol III promoters with edit scores above standard (n=982, 481,687 pairs) **b)** L_{max} distributions depicting how subsets of up to 205 Pol III promoters, in the same orientation, can be used and satisfy L_{max} < 40, enabling assembly (n=982, 20,910 pairs). **c-d)** L_{max} distributions as in **a-b**, but for reverse complement comparisons.

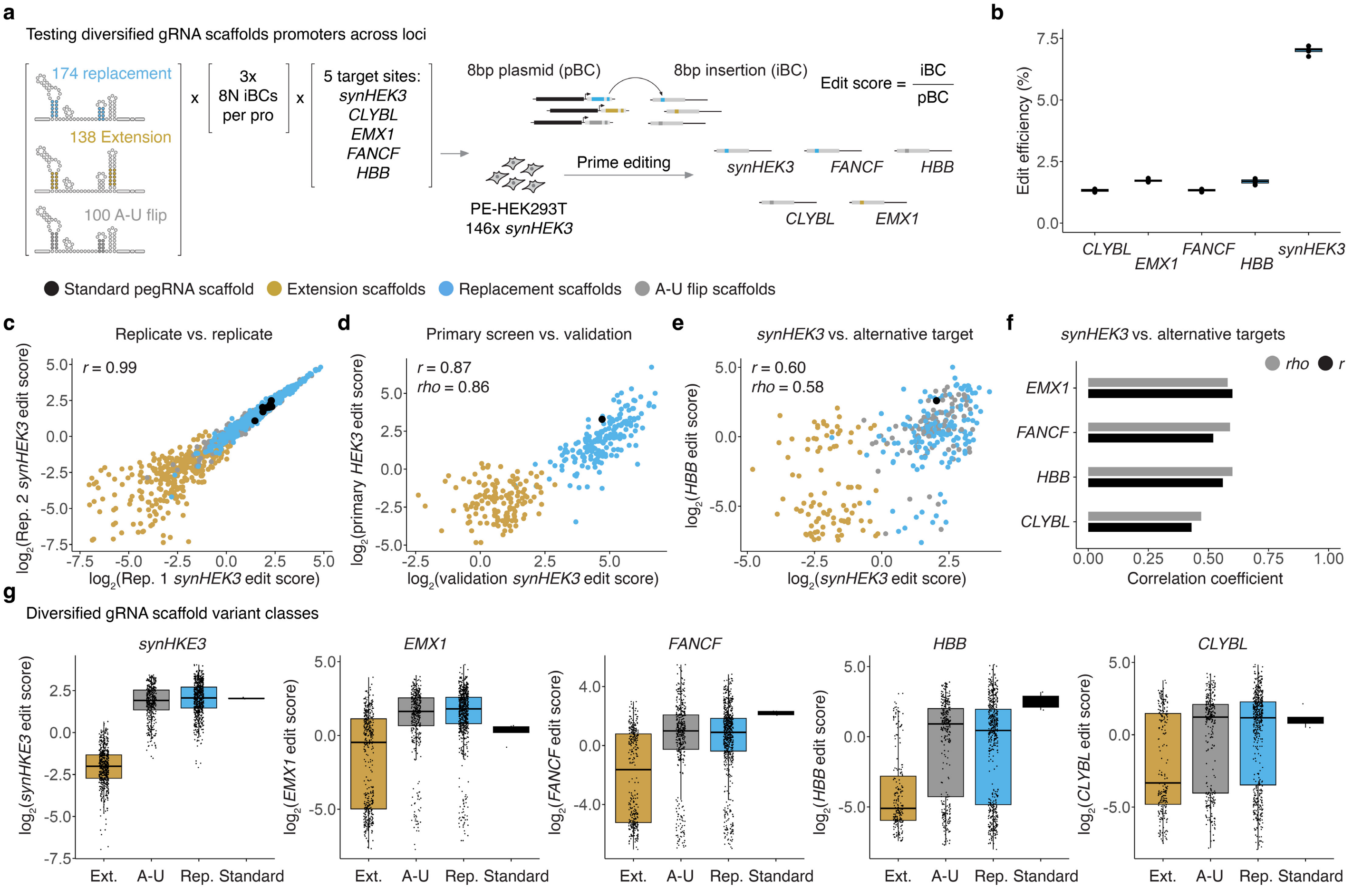

Figure S22 | Validation of diversified gRNA scaffolds across additional target loci. a) Library design, contents, and multiplex prime editing functional assessment workflow. **b)** Diversified scaffolds drove editing across all tested target loci: *CLYBL*, *EMX1*, *FANCF*, *HBB*, *synHEK3*. Editing efficiencies, calculated as the percentage of reads with programmed 8 bp insertions at each locus for each transfection replicate, are shown. **c)** Reproducibility of edit scores between transfection replicates for *synHEK3* target sites. Pearson correlation coefficients, calculated between edit scores for each construct prior to log transformation, are listed. **d)** Reproducibility of edit scores from the primary screen vs. this validation screen. Pearson and Spearman correlation coefficients, calculated between edit scores prior to log transformation, are listed. **e)** Comparison of edit scores at *synHEK3* vs. exemplary alternative target locus, *HBB*. Pearson and Spearman correlation coefficients, calculated between log-transformed edit scores, are listed. **f)** Barplot of Pearson and Spearman correlation coefficients, calculated between log-transformed edit scores, between *synHEK3* and alternative target loci. **g)** Edit scores for different classes of diversified scaffolds across target loci.

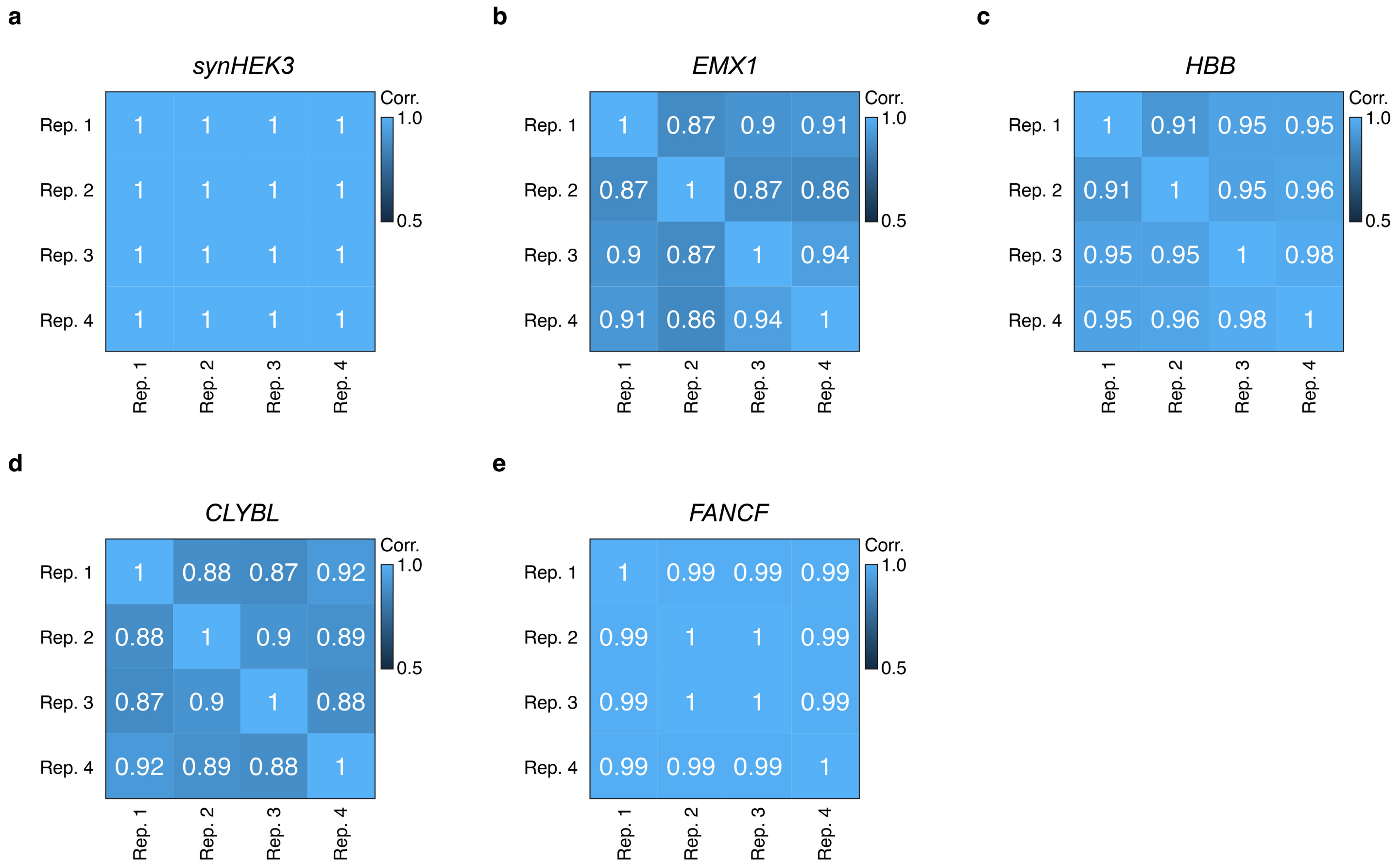

Figure S23 | Replicate correlations for diversified Pol III promoters across alternative target loci. Heatmaps of Pearson correlation coefficients between pairs of transfection replicates, calculated between edit scores prior to log transformation, for diversified Pol III promoters. Technical replicate comparisons with Pearson correlation coefficients >0.995 are rounded to 1 in the visualization. **a)** Correlation of edit scores among four transfection replicates at *synHEK3* target sites. **b)** Correlation of edit scores among four transfection replicates at the *EMX1* locus. **c)** Correlation of edit scores among four transfection replicates at the *HBB* locus. **d)** Correlation of edit scores among four transfection replicates at the *CLYBL* locus. **e)** Correlation of edit scores among four transfection replicates at the *FANCF* locus.

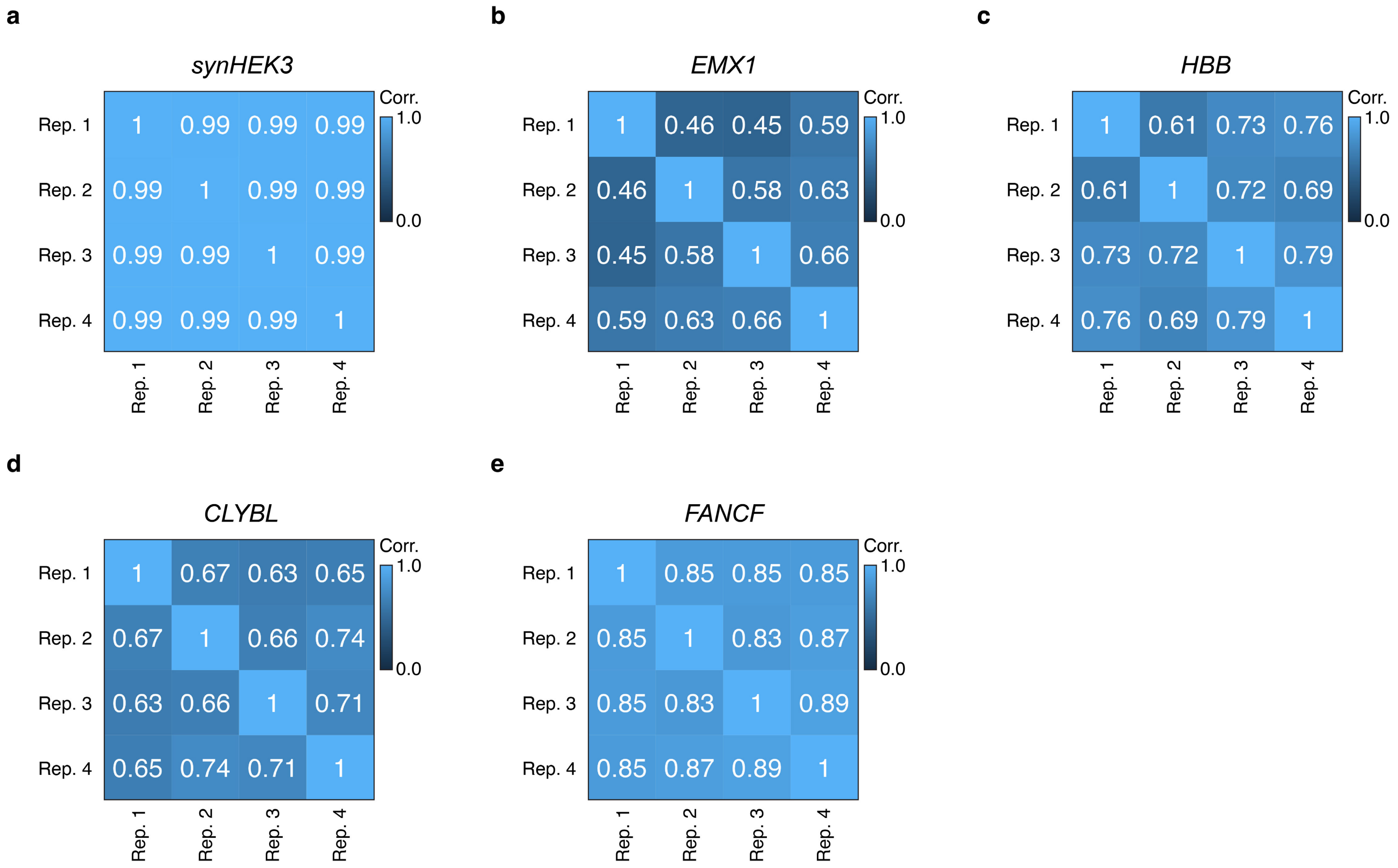

Figure S24 | Replicate correlations for diversified gRNA scaffolds across alternative target loci. Heatmaps of Pearson correlation coefficients between pairs of transfection replicates, calculated between edit scores prior to log transformation, for diversified gRNA scaffolds. **a)** Correlation of edit scores among four transfection replicates at *synHEK3* target sites. **b)** Correlation of edit scores among four transfection replicates at the *EMX1* locus. **c)** Correlation of edit scores among four transfection replicates at the *HBB* locus. **d)** Correlation of edit scores among four transfection replicates at the *CLYBL* locus. **e)** Correlation of edit scores among four transfection replicates at the *FANCF* locus.

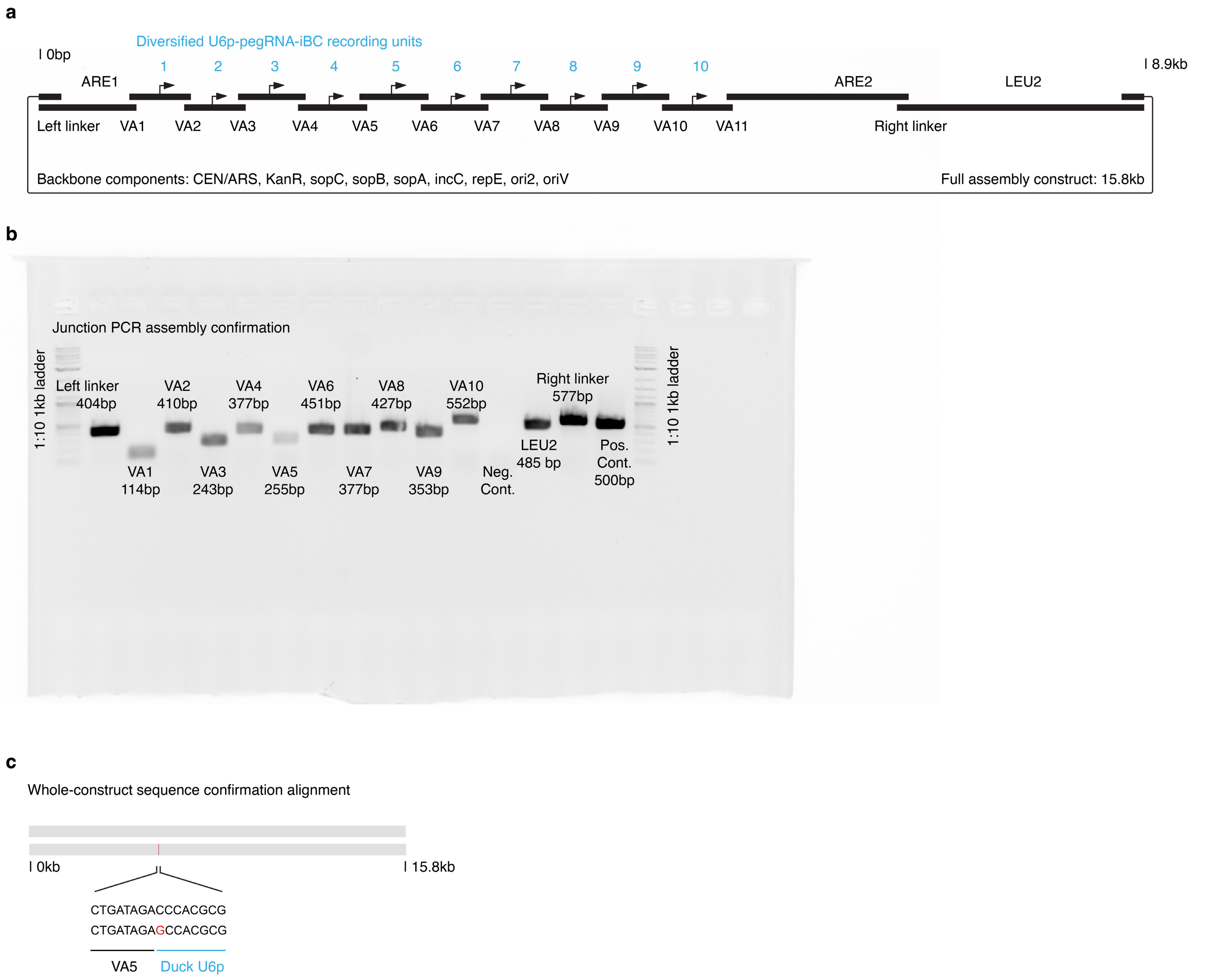

Figure S25 | Design and assembly of a 10-unit diversified molecular recording array. a) Assembly design showing the 14 fragments flanked with overlapping VEGAS adapters and/or linkers to enable single-step assembly in yeast. VA: vegas adapter, ARE: anti-repressor element, ORI: origin of replication. b) Junction PCR amplicons confirmed effective assembly. **c)** Long-read, whole-plasmid/construct sequencing further confirmed correct assembly. Alignment revealed only a single nucleotide substitution error in the first base pair of the 5th U6 promoter from the Domestic Muscovy Duck *Cairina moschata domestica*. This substitution falls upstream of the four core TFBSs and is not predicted to impact function.

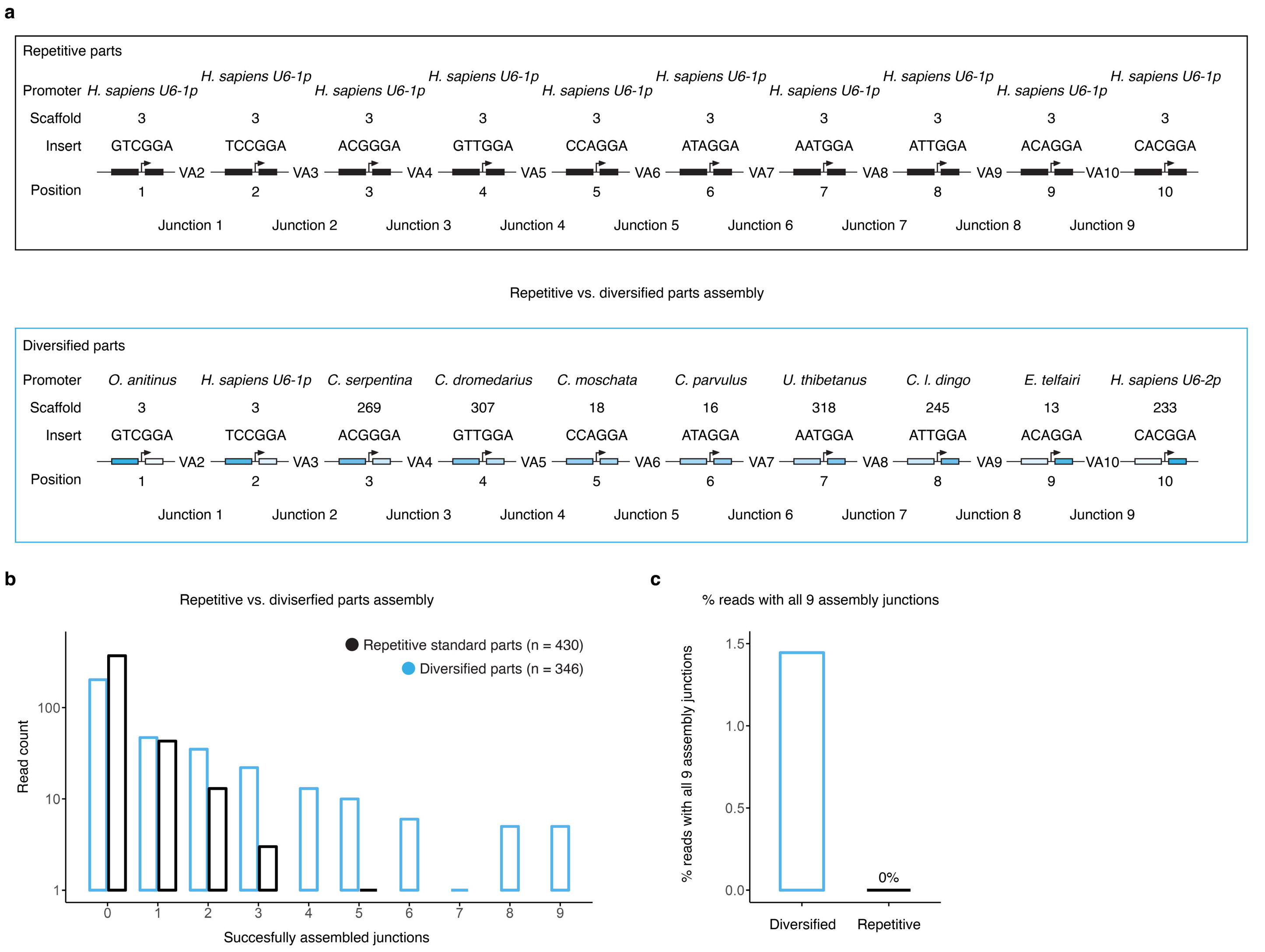

Figure S26 | Diversified parts enable efficient, single-step assembly of the 10-key Pol III promoter-gRNA array. a) Experimental overview. We attempted to construct a 10-key molecular recording array using either repetitive parts (repeats of the standard human hRNU6-1p and gRNA scaffold each paired with different iBCs) or diversified parts, using the same sets of VEGAS adapters (VA) for assembly in both cases. Correct full-length assemblies contain all nine assembly junctions spanning the 10-key Pol III promoter-gRNA array. **b)** The number of long reads bearing the indicated number of successfully assembled junctions recovered when using repetitive parts or diversified parts. **c)** The number of reads bearing all nine assembly junctions recovered when either diversified or repetitive parts were used.
